# DCAF11-dependent molecular glue degrader activated by glutathionylation

**DOI:** 10.1038/s41586-026-10873-1

**Published:** 2026-08-05

**Authors:** Hojong Yoon, Franziska Wachter, Katharine A. Barrett, Cyrus Jin, Anna Rodríguez-Pöhnlein, Justine C. Rutter, Ryan J. Lumpkin, Rebecca J. Metivier, Katherine A. Donovan, Kheewoong Baek, Yongying Jiang, Minwoo Lee, Robert W. Kalis, Jianwei Che, Yuan Xiong, Eric S. Fischer, Benjamin L. Ebert

**Affiliations:** 1 Department of Medical Oncology, Dana-Farber Cancer Institute, Boston, MA USA; 2 Broad Institute of MIT and Harvard, Cambridge, MA USA; 3 Dana-Farber/Boston Children’s Cancer and Blood Disorders Center, Harvard Medical School, Boston, MA USA; 4 Department of Cancer Biology, Dana-Farber Cancer Institute, Boston, MA USA; 5 Department of Biological Chemistry and Molecular Pharmacology, Harvard Medical School, Boston, MA USA; 6 Institute for Applied Cancer Science (IACS), The University of Texas MD Anderson Cancer Center, Houston, TX USA; 7 Present Address: Department of Experimental Therapeutics, The University of Texas MD Anderson Cancer Center, Houston, TX USA; 8 Present Address: James P. Allison Institute, The University of Texas MD Anderson Cancer Center, Houston, TX USA

**Keywords:** Mechanism of action, Enzyme mechanisms, Cryoelectron microscopy, Small molecules

## Abstract

Targeted protein degradation is a powerful pharmacological strategy that harnesses the ubiquitin proteasome system to eliminate disease-relevant proteins, including otherwise undruggable proteins^1^. Here we report an unbiased and broadly applicable platform for the systematic discovery of molecular glues across diverse E3 ligases. Using multiplexed mass spectrometry-based chemical screening, we identified M12, a molecular glue that reprogrammes the E3 ligase DCAF11 to degrade DDX18. Mechanistically, M12 functions as a prodrug that is activated through glutathione *S*-transferase-mediated glutathionylation. The glutathione moiety binds to an evolutionary conserved glutathione-binding site on DCAF11, and the exposed M12 moiety facilitates neo-substrate recruitment. We demonstrate that this glutathione-dependent mechanism readily enables targeted degradation of a range of proteins. Collectively, these findings establish that metabolically activated compounds can redirect E3 ligase function, thereby expanding the scope of targeted protein degradation and chemically induced proximity.

## Abstract (web summary)

M12 is a metabolically activated molecular glue that recruits diverse proteins to DCAF11 for ubiquitin-mediated degradation, revealing a glutathione-dependent mechanism that broadens targeted protein degradation across multiple substrates.

## Main

Molecular glues represent a distinct class of small molecules that promote or stabilize protein–protein interactions through modulation of the contact surfaces^2^. A subset of molecular glues induces protein degradation through recruitment of a neo-substrate to an E3 ubiquitin ligase, causing drug-induced ubiquitylation and subsequent degradation^3^. This mechanism of drug action was first observed in studies of thalidomide and related drugs, including lenalidomide and pomalidomide, which function as molecular glue degraders for the Ikaros family transcription factors^4,5^. Degradation of these targets, which were previously considered undruggable, results in clinical efficacy for the treatment of multiple myeloma and other haematologic malignancies^4^. Mechanistically, these degraders bind to CRBN, a substrate receptor of the CUL4–RBX1–DDB1–CRBN (CRL4^CRBN^) ubiquitin ligase^6^. When bound to CRBN, these drugs remodel the surface of CRBN to promote neo-substrate recruitment for targeted degradation^7,8^. Chemical diversification of the glutarimide-containing compounds that bind CRBN has led to the discovery of dozens of CRBN neo-substrates and led to several new clinical programmes^9^, highlighting the potential of molecular glues to target a wide range of proteins. Several investigational agents, including aryl sulfonamides and UM171, exhibit a mechanism of action analogous to that of thalidomide by inducing neo-substrate degradation, targeting RBM39 and the CoREST (corepressor of RE1-silencing transcription factor) complex, respectively^10–13^.

Motivated by the therapeutic potential, several rational approaches have been pursued to discover molecular glue degraders. By integrating cytotoxicity data with E3 ligase expression profiles across cancer cell lines, CR8 was revealed as a molecular glue degrader for cyclin K^14^. Through an orthogonal approach, a chemical screen in hypo-neddylated cells uncovered molecular glue degraders for RBM39 and cyclin K^15^. In addition, diversifying ligands targeting CUL2–ELOB–ELOC–VHL (CRL2^VHL^) ubiquitin ligase has facilitated the identification of molecular glues targeting CDO1 and GEMIN3 (refs. ^16,17^). Despite these advances, only a small subset of E3 ligases have been successfully co-opted for neo-substrate degradation. With more than 600 E3 ligases encoded in the human proteome, expanding the range of exploitable ligases will broaden the target space of molecular glue degraders. The principal bottleneck, however, is the lack of unbiased, scalable approaches to systematically identify molecular glue degraders across diverse E3 ligases without relying on ligase-specific compound libraries or prior knowledge of ligase–substrate interactions.

In this study, we developed a high-throughput workflow for the unbiased discovery of molecular glues by mass spectrometry. Multiplexing of ligases in combination with pooled compounds and cell lysates enables unbiased screening across diverse chemical libraries, facilitating the identification of new E3 ligases that are exploitable for targeted protein degradation. The use of cell lysates enables the screen to retain endogenous enzymatic and metabolic activities that may be required for compound activation or target engagement. This approach led to the identification of a novel molecular glue degrader that reprogrammes the CUL4–RBX1–DDB1–DCAF11 (CRL4^DCAF11^) ubiquitin ligase. Notably, the identified molecular glue requires an enzyme-mediated gain-of-function activity, which is unlikely to be identified by other approaches.

## Identification of a DCAF11–DDX18 glue

**Fig. 1:**
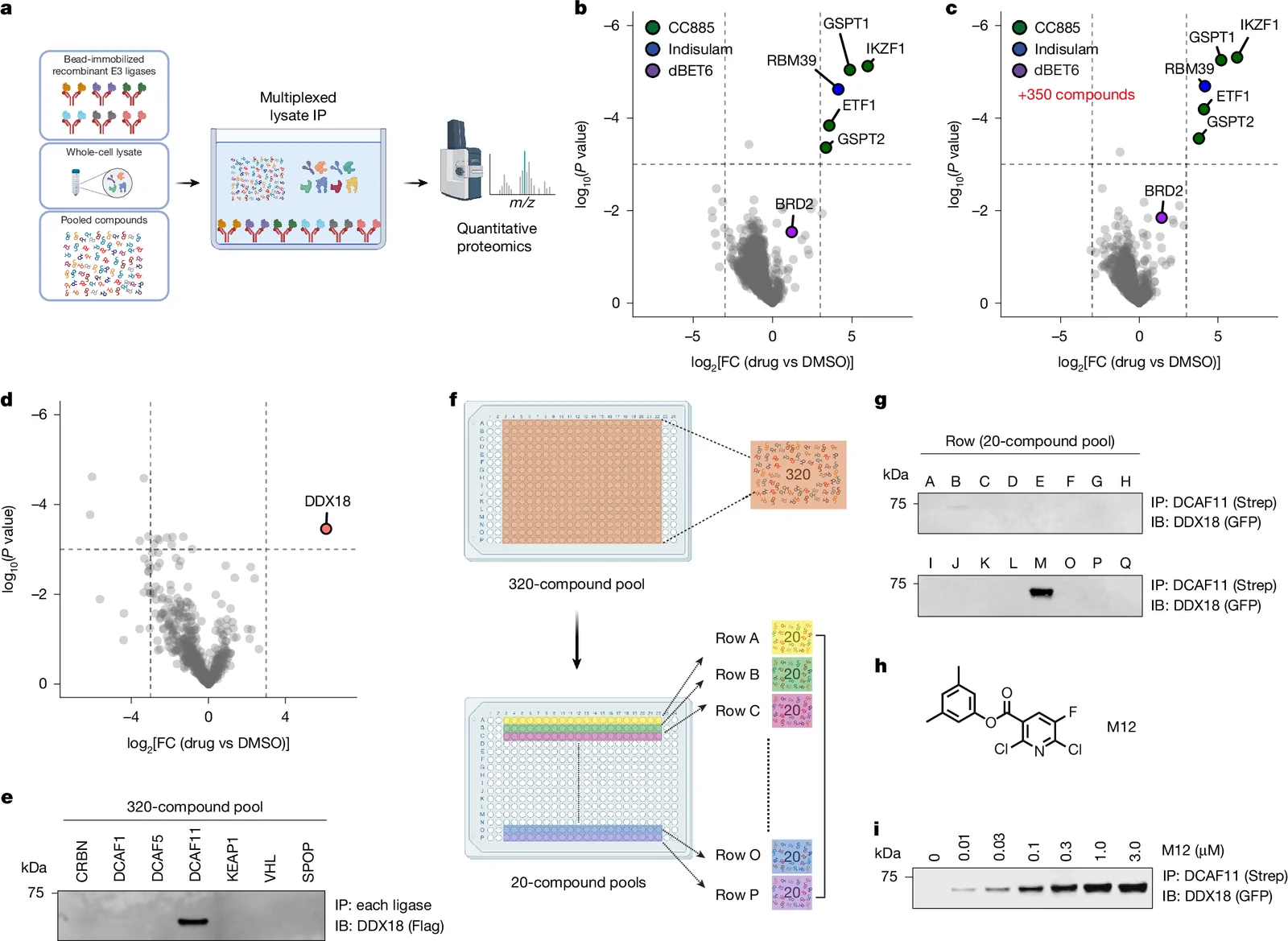
Multiplexed immunoprecipitation using cellular lysates coupled with mass spectrometry identifies a novel molecular glue. **a**, Schematic overview of the multiplexed IP–MS workflow. IP, immunoprecipitation. Created in BioRender; Ebert, B. https://BioRender.com/o06xnir (2026). **b**, Two bait proteins (CRBN and DCAF15) were incubated with cellular lysate and treated with either DMSO or a mixture containing 1 µM CC885, indisulam and dBET6. The scatter plot shows fold change (FC) in protein abundance relative to DMSO control. Significant changes were assessed by a two-sided moderated *t*-test as implemented in the limma package^43^. **c**, As in **b**, with the addition of 350 more compounds to the treatment pool. Significant changes were assessed by a two-sided moderated *t*-test as implemented in the limma package^43^. **d**, CRBN, DCAF1, DCAF5, DCAF11, KEAP1, VHL and SPOP were incubated with cellular lysate and treated with either DMSO or a pooled library of 320 compounds (1 µM each). The scatter plot shows fold changes in protein abundance relative to DMSO control. Significant changes were assessed by a two-sided moderated *t*-test as implemented in the limma package^43^. **e**, Immunoblot (IB) of immunoprecipitation using each ligase in the presence of 320 compounds (1 µM each), with lysates from cells expressing Flag–DDX18. The image is from a single experiment. **f**, Schematic showing the progressively smaller pooling strategy that was used to identify the active molecular glue between DCAF11 and DDX18. Created in BioRender; Ebert, B. https://BioRender.com/oq6vxvt (2026). **g**, Immunoblot of DCAF11 immunoprecipitation in the presence of compound pools (1 µM each) from rows as shown in **f**, using lysate from cells expressing DDX18–eGFP. The image is from a single experiment. **h**, Chemical structure of M12. **i**, Immunoblot of DCAF11 immunoprecipitation with increasing concentration of M12, using lysate from cells expressing DDX18–eGFP. Representative image from two independent experiments with similar results.

Molecular glue degraders exert their activity by facilitating the recruitment of neo-substrates to an E3 ubiquitin ligase, which can be detected using immunoprecipitation coupled to mass spectrometry (IP–MS)^18,19^. To enable library screening, we developed a highly multiplexed screening approach, in which we combine a pool of E3 ligases, pooled small molecules and proteins derived from cellular lysates to identify novel molecular glues in an unbiased and high-throughput manner. We immobilized multiple recombinantly expressed E3 ligases on beads. The beads were incubated with a pool of diverse compounds and whole-cell lysates to enable compound-induced proximity between the different E3s in the pools and any proteins in the lysate for unbiased target discovery. After affinity enrichment of proteins that interact with the immobilized E3 ligases exclusively in the presence of the small molecules, the eluted proteins were identified using liquid chromatography–mass spectrometry (LC–MS) (Fig. 1a).

**Extended Data Fig. 1:**
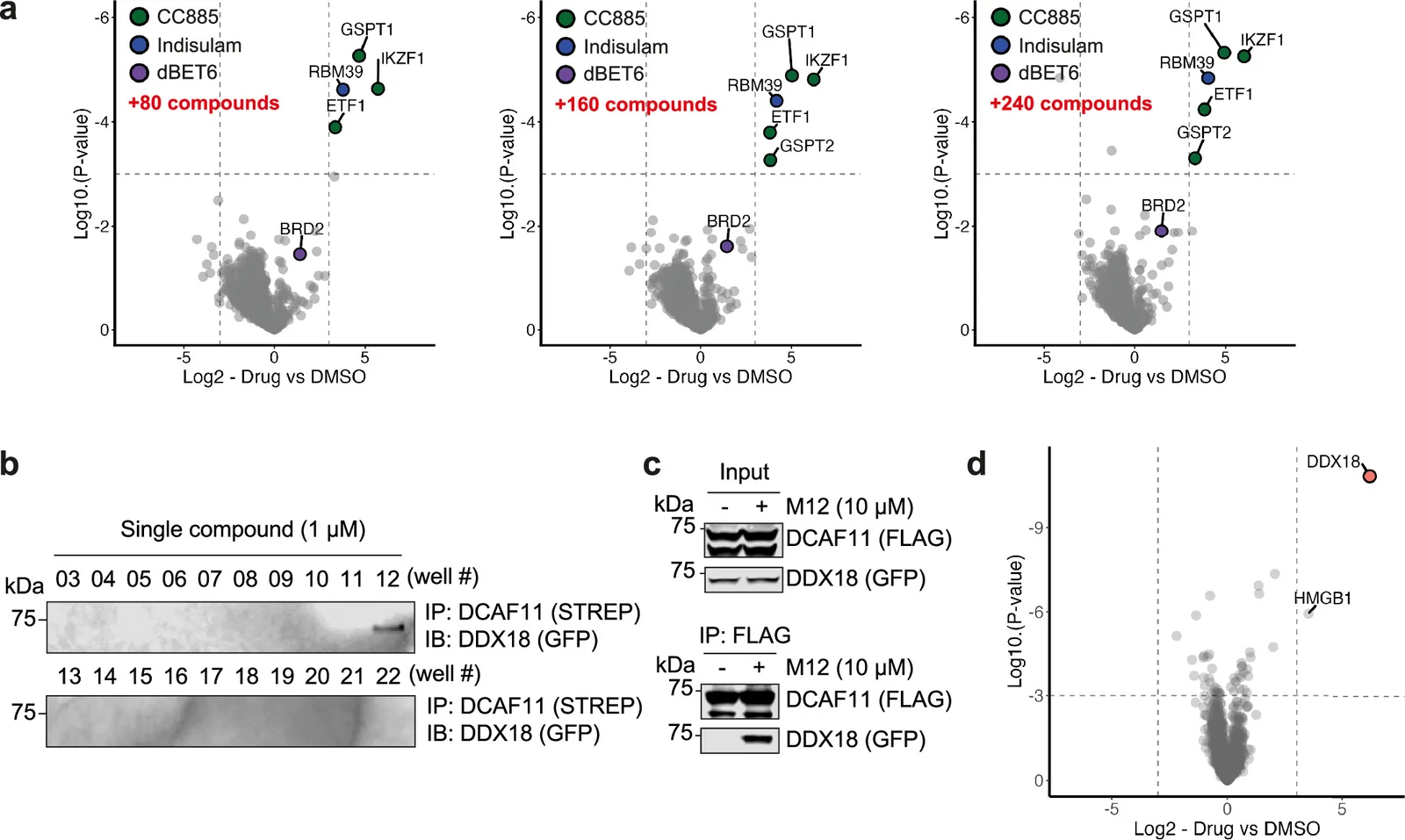
Multiplexed IP-MS identifies M12 as a molecular glue for DCAF11-DDX18. a. As in Fig. 1b, with the addition of increasing number of compounds to the treatment pool. Significant changes were assessed by a two-sided moderated *t* test as implemented in the limma package^43^. b. DCAF11 IP followed by western blots in the presence of each compound (1 µM), using cellular lysate expressing DDX18-eGFP. The image is from a single experiment. c. DCAF11 IP followed by western blots in the presence of DMSO or 10 µM M12 from 293 T cells transfected with indicated FLAG-DCAF11 and GFP-DDX18 constructs. Representative images from at least two independent experiments with similar results is shown. d. IP-MS: DCAF11 IP followed by mass spectrometry with 293 T cellular lysate, treated with either DMSO (*n* = 4) or 10 µM M12 (*n* = 4). Significant changes were assessed by a two-sided moderated *t* test as implemented in the limma package^43^.

To demonstrate the feasibility of this multiplexed screening approach, we tested a mixture of two extensively characterized E3 ligases, CRBN and DCAF15. We assessed whether immobilizing these two ligases on beads allows compound-induced recruitment of their known neo-substrates using a drug mixture of CC885 (a GSPT1, IKZF1, IKZF2 and IKZF3 degrader via CRBN)^20^, dBET6 (a BRD2, BRD3 and BRD4 degrader via CRBN)^21^ and indisulam (an RBM39 degrader via DCAF15)^13^. In this multiplexed format of two ligases and three drugs, unbiased IP–MS experiments robustly enriched neo-substrates, including GSPT1 and IKZF1 with CC885–CRBN, BRD2 with dBET6–CRBN and RBM39 with indisulam–DCAF15 (Fig. 1b). Next, we repeated the experiments with increasing numbers of chemically diverse small molecules. The results demonstrated that the induced interactome remained largely unaffected even with the addition of hundreds of additional compounds, enabling accurate detection of compound-induced neo-substrates without interference (Fig. 1c and Extended Data Fig. 1a). These results validate the robustness of the assay in a pooled-drug setting and motivated us to conduct a larger screen.

To uncover novel molecular glues, we screened 7 E3 ligases simultaneously—CRBN, DCAF1, DCAF5, DCAF11, KEAP1, VHL and SPOP—with 5,000 diverse compounds in a pooled setting (320 compounds per pool). Using IP–MS for each pool, we found that the DEAD-box RNA helicase DDX18, a key regulator of RNA metabolism, ribosome biogenesis, cell cycle progression, R-loop-mediated genome stability and stem cell pluripotency^22–24^, was highly enriched in one specific pool (Fig. 1d). To identify the ligase mediating DDX18 enrichment, we performed immunoprecipitation experiments with each individually immobilized E3 ligase using the same 320-compound pool and found that DCAF11 is the E3 ligase responsible for recruitment of DDX18 (Fig. 1e). To identify the small molecule required for DDX18 enrichment by DCAF11, we performed immunoprecipitation experiments with progressively smaller compound pools, revealing a single compound, M12, to be the active compound (Fig. 1f–i and Extended Data Fig. 1b,c). To validate this, we performed an unbiased IP–MS experiment with DCAF11 as a bait and found selective recruitment of DDX18 to DCAF11 in the presence of M12 (Extended Data Fig. 1d).

## M12-induced DDX18 degradation by DCAF11

**Fig. 2:**
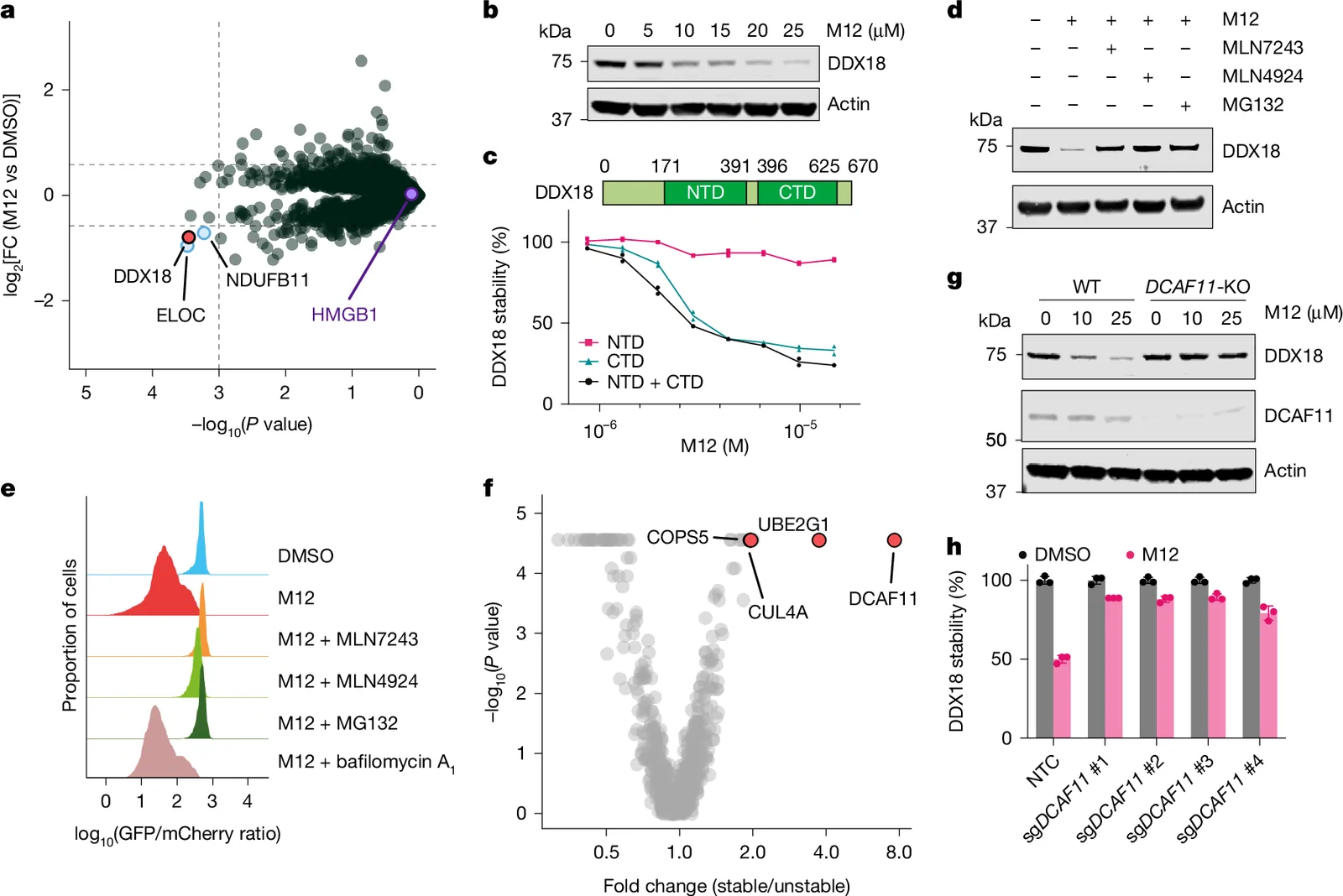
M12 promotes DDX18 degradation via the CRL4^DCAF11^ complex. **a**, Whole-proteome quantification of Jurkat cells treated with 20 µM M12 or DMSO (*n* = 3 technical replicates) for 6 h. Significant changes were assessed by a two-sided moderated *t*-test as implemented in the limma package^43^. **b**, Molt4 cells were treated for 18 h with DMSO or increasing concentrations of M12. Representative images from two independent experiments with similar results. CTD, C-terminal domain; NTD, N-terminal domain. **c**, Top, DDX18 domain architecture. Flow cytometry analysis of DDX18–eGFP reporters in K562-Cas9 cells incubated with M12 for 18 h (*n* = 2 technical replicates). DDX18 stability was calculated as the eGFP/mCherry fluorescence ratio. **d**, Jurkat cells were pretreated with 0.5 µM MLN7243, 1 µM MLN4924 or 5 µM MG132 for 1 h, followed by treatment with 20 µM M12 for 18 h. Representative images from two independent experiments with similar results. **e**, K562-Cas9 DDX18 reporter cells were pretreated with 0.5 µM MLN7243, 1 µM MLN4924 or 5 µM MG132 for 1 h, followed by treatment with 10 µM M12 for 18 h. Representative images from three independent experiments with similar results. **f**, UPS-focused CRISPR degradation screen. K562-Cas9 DDX18 reporter cells treated with 10 μM M12 for 18 h (*n* = 3 technical replicates). Significant changes were assessed by a two-sided empirical rank-sum test. **g**, Wild-type (WT) and *DCAF11*-knockout (*DCAF11*-KO) Jurkat cells were treated with different concentrations of M12 for 18 h, and DDX18 levels were assessed by immunoblot. Representative images from two independent experiments with similar results. **h**, K562-Cas9 DDX18 reporter cells were transduced with non-targeting control (NTC) or one of four DCAF11-targeting single guide RNAs (sgRNAs) (sg*DCAF11*), treated with DMSO or 5 µM M12 for 18 h (*n* = 3 technical replicates), and DDX18–eGFP levels were quantified by flow cytometry. Bars are presented as mean values ± s.d.

**Extended Data Fig. 2:**
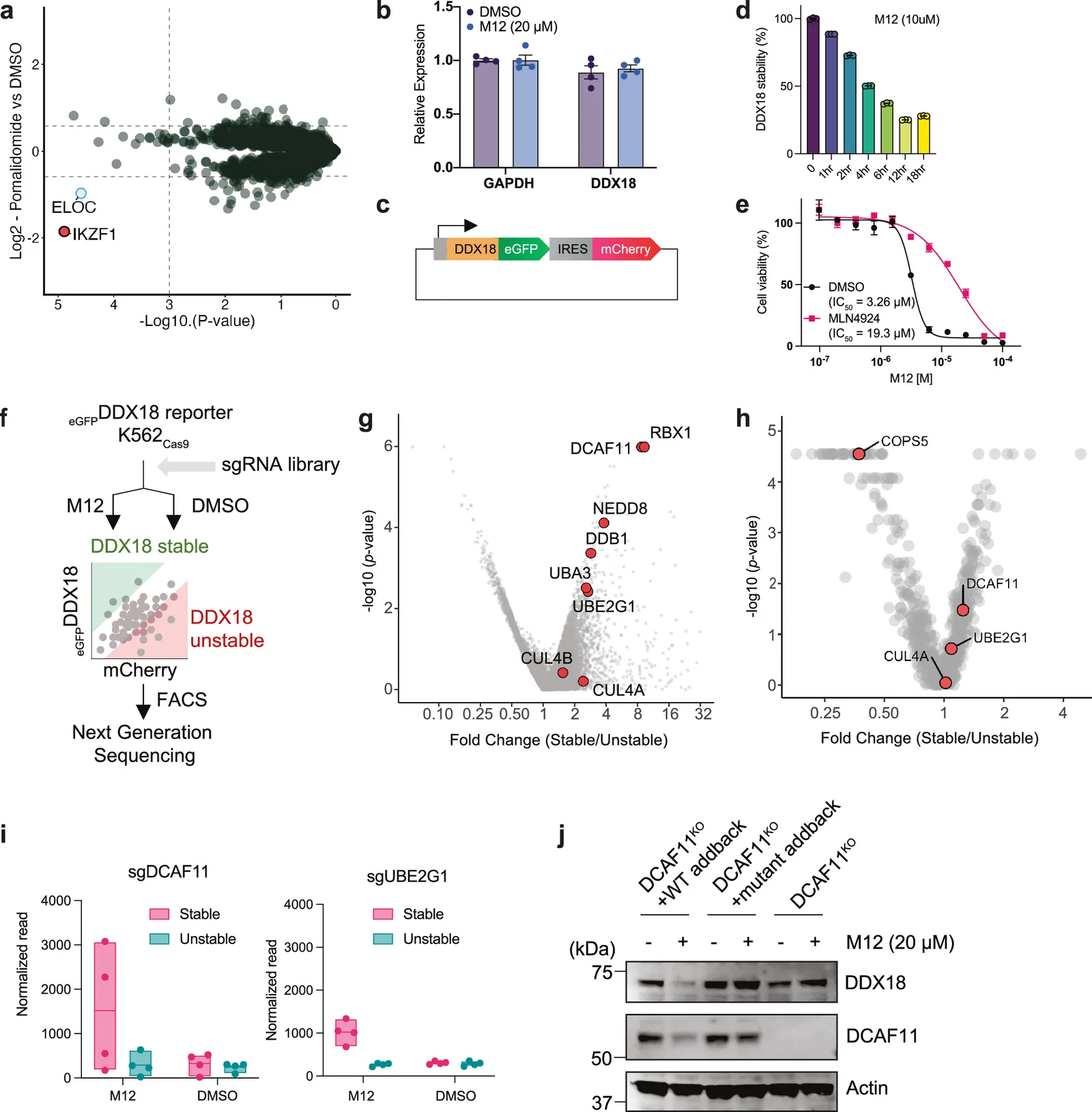
M12 degrades DDX18 via CRL4^DCAF11^. a. Whole-proteome quantification of Jurkat cells treated with 1 µM pomalidomide (*n* = 3 technical replicates) or DMSO (*n* = 3 technical replicates) for 18 h. ELOC is a common contaminant in both proteomics experiments (Fig. 2a and Extended Data Fig. 2a). Significant changes were assessed by a two-sided moderated *t* test as implemented in the limma package^43^. b. mRNA levels quantified by qPCR in Jurkat cells after treatment with 20 µM M12 or DMSO for 18 h (*n* = 4 technical replicates). Data are presented as mean values ± s.e.m. c. Schematic of the DDX18 stability reporter. d. Flow cytometry analysis of DDX18-eGFP reporters in K562_cas9_ cells treated with 10 µM M12 (*n* = 3 technical replicates; data shown is representative of two independent replicates) for the indicated times. DDX18 stability was calculated as the eGFP/mCherry fluorescence ratio. Data are presented as mean values ± s.e.m. e. Cell viability measured by cell-titer glo assays (*n* = 3 technical replicates). Pretreatment of MLN49249 (1 h, 500 nM) showed reduction of cytotoxicity by M12, indicating the mechanism of cell death reliant on ubiquitylation. Data are presented as mean values ± s.d. f. Schematic of the DDX18 stability reporter-based sorting screen. g. Genome-wide CRISPR-Cas9 knockout screen for DDX18 stability in K562_cas9_ cells after 18 h of treatment with 10 µM M12 (*n* = 3 technical replicates). Significant changes were assessed by a two-sided empirical rank-sum test. h. UPS-focused CRISPR degradation screen for DDX18-eGFP reporter in K562_cas9_ cells treated with DMSO for 18 h (*n* = 3 technical replicates). Significant changes were assessed by a two-sided empirical rank-sum test. i. UPS-focused CRISPR degradation screen for DDX18-eGFP reporter: Normalized read counts (M12 or DMSO) in each sorted gate for 4 sgRNAs targeting *DCAF11* and *UBE2G1* (*n* = 3 technical replicates). Centre line, median; box limits, upper and lower quartiles (25th and 75th percentiles); dots, individual data points. j. Immunoblots of DDX18 degradation in DCAF11 KO K562 cells, or wild-type (WT) addback, DCAF11 mutant (D309A/N311A/D328A) addback. Cells were treated with M12 (20 µM) for 6 h, and DDX18 levels were assessed by immunoblot. The image is from a single experiment.

Molecular glue degraders recruit neo-substrates to an E3 ubiquitin ligase, resulting in ubiquitylation and degradation. Using quantitative mass spectrometry-based proteomics, we found that treatment of cells with M12 leads to decreased protein levels of DDX18 (Fig. 2a and Extended Data Fig. 2a). In western blot analyses, DDX18 protein levels decreased in response to M12 in a dose-dependent manner (Fig. 2b), while DDX18 mRNA levels remained unchanged (Extended Data Fig. 2b). To identify the critical domain of DDX18 required for M12-induced degradation, we generated a fluorescent reporter in which the N-terminal or C-terminal domain of DDX18 were cloned in frame with eGFP followed by an internal ribosome entry site (IRES) and mCherry (Extended Data Fig. 2c). We found that treatment of cells with M12 requires the CTD region of DDX18 for M12-induced degradation (Fig. 2c and Extended Data Fig. 2d). Pretreatment with either MLN7243 (a UBA1 inhibitor), MLN4924 (a NAE1–UBA3 inhibitor), or MG132 (a 26S proteasome inhibitor) rescued M12-induced DDX18 degradation, but inhibition of lysosomal degradation using bafilomycin A1 did not attenuate the degradation of DDX18, consistent with DCAF11-mediated ubiquitylation and proteasomal degradation (Fig. 2d,e and Extended Data Fig. 2e).

To dissect the molecular machinery that is required for M12-induced DDX18 degradation in an unbiased manner, we performed flow cytometry-based CRISPR–Cas9 screens using the DDX18 stability reporter (Extended Data Fig. 2f). In both genome-wide and targeted ubiquitin proteasome system (UPS)-focused genetic screens, DCAF11 was the only E3 ligase receptor that scored significantly, as did all components of the CRL4^DCAF11^ complex and its regulators (Fig. 2f and Extended Data Fig. 2g–i). CRISPR–Cas9-mediated knockout of *DCAF11* prevented M12-induced DDX18 degradation (Fig. 2g,h and Extended Data Fig. 2j). Collectively, these experiments confirm that M12-induced proximity between DCAF11 and DDX18 leads to CRL4^DCAF11^-mediated degradation of DDX18 in cells.

## Activation of M12 by glutathionylation

**Fig. 3:**
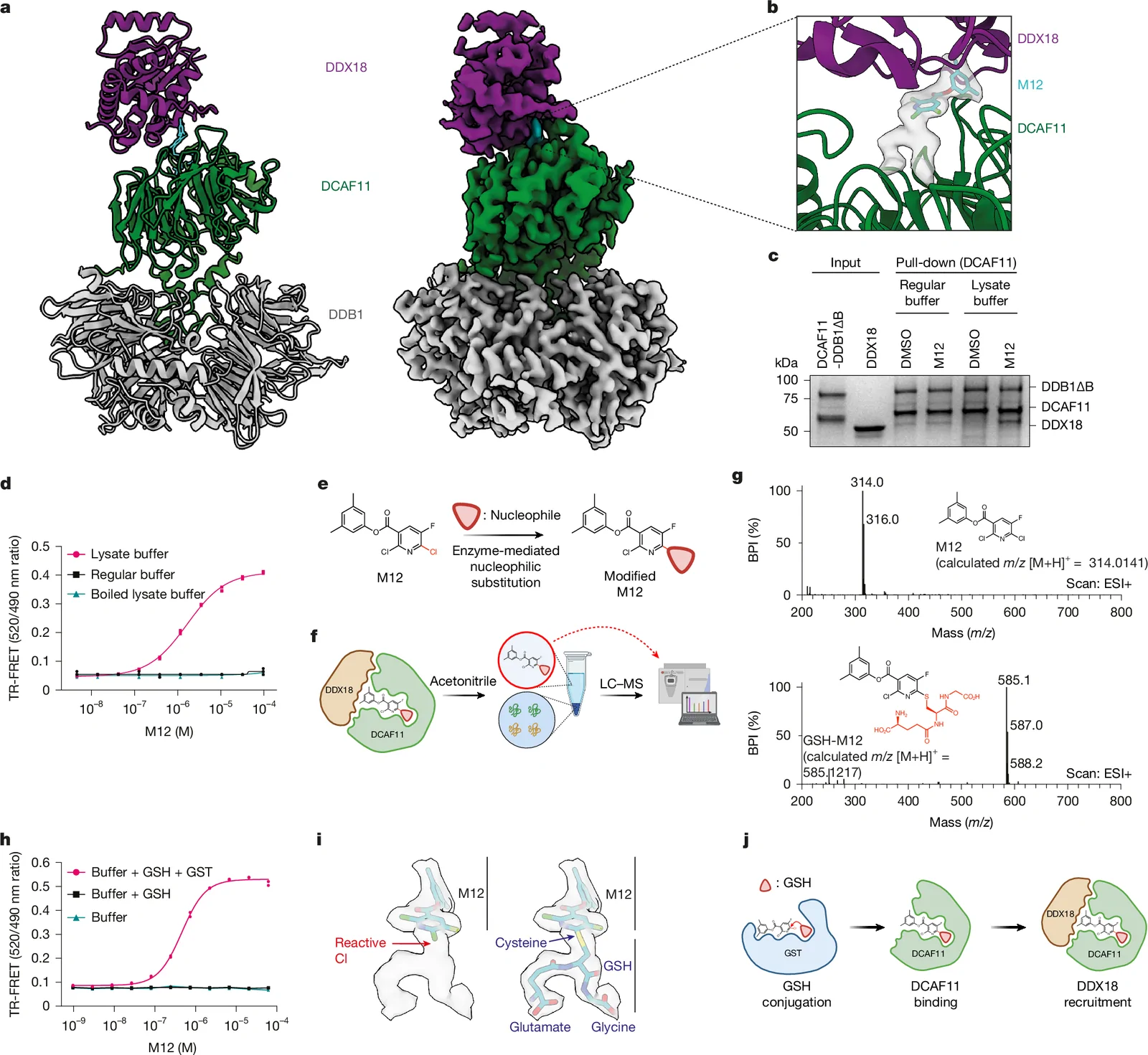
Glutathionylation of M12 converts it into a functional molecular glue for DCAF11–DDX18. **a**, Cryo-EM model and composite map of the DDX18–DCAF11–DDB1∆B complex mediated by M12. **b**, Zoomed-in composite cryo-EM map of the indicated region in **a**, with M12 shown in cyan. **c**, SDS–PAGE analysis of in vitro pulldown of Flag–DDX18 with recombinant Strep-tagged DCAF11–DDB1∆B in the presence of 10 µM M12 or DMSO, under regular buffer and lysate buffer conditions. Representative images from two independent experiments with similar results. **d**, TR-FRET. Titration of M12 versus biotinylated DCAF11 (200 nM) and GFP–DDX18 (200 nM) in regular buffer, lysate or boiled lysate (*n* = 3 technical replicates). **e**, Schematic representation of enzymatic modification of M12 in cellular lysate. Created in BioRender; Ebert, B. https://BioRender.com/dmj7hyq (2026). **f**, Schematic workflow for isolating the modified form of M12 via mass spectrometry. Created in BioRender; Ebert, B. https://BioRender.com/dmj7hyq (2026). **g**, Mass spectra of M12 and the modified form (GSH-M12), corresponding to **f**. BPI, base peak intensity. **h**, TR-FRET. Titration of M12 versus biotinylated DCAF11 (200 nM) and GFP–DDX18 (200 nM) in buffer, buffer with GSH or buffer with GSH plus GST (*n* = 2 technical replicates). **i**, Putative density maps of unmodified M12 (left) and GSH-M12 (right). **j**, Schematic model illustrating the transformation of M12 to GSH-M12, which functions as a molecular glue for DCAF11–DDX18. Created in BioRender; Ebert, B. https://BioRender.com/rvznwr3 (2026).

**Extended Data Fig. 3:**
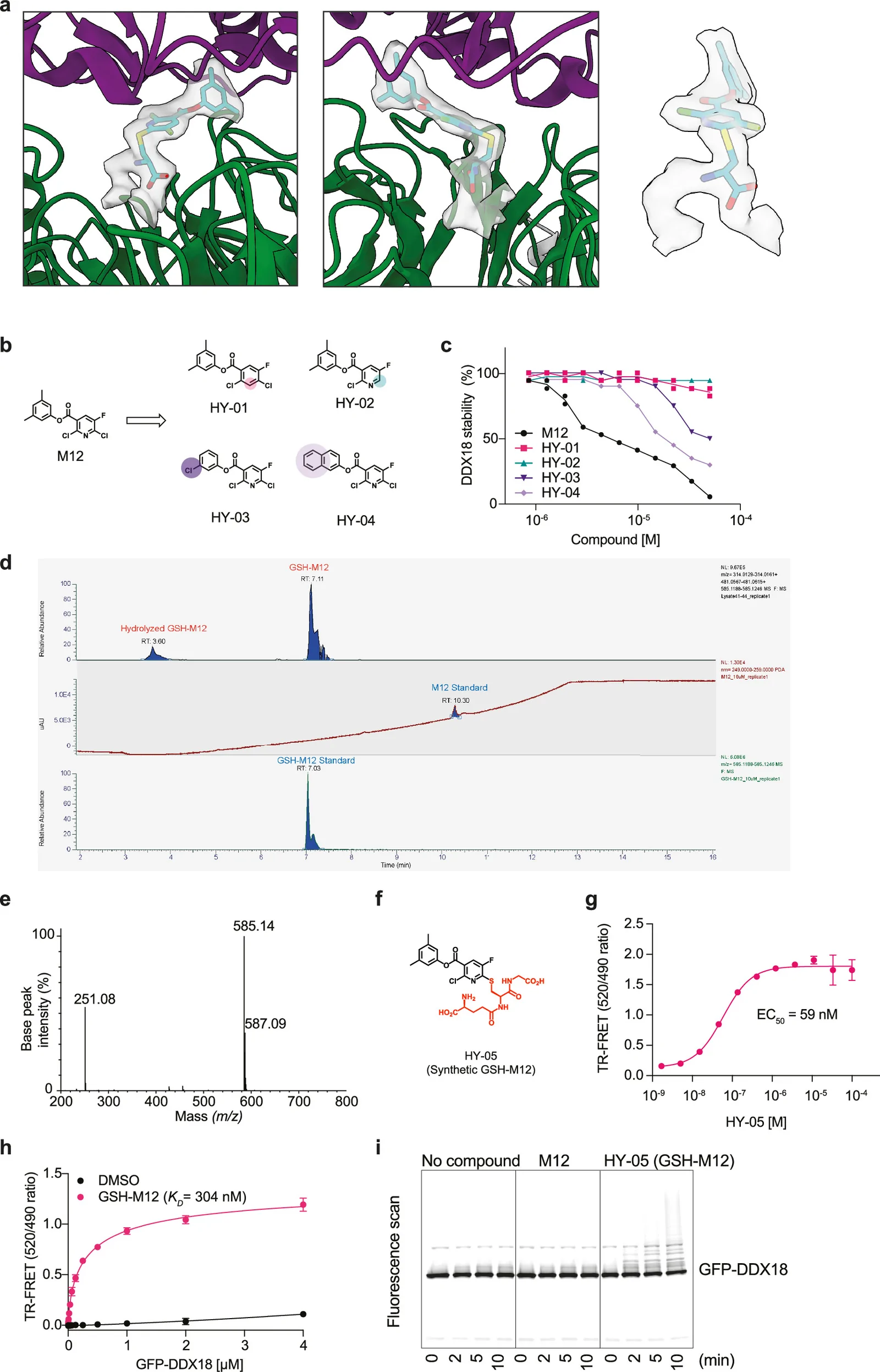
Glutathionylation of M12 promotes its binding to DCAF11 and recruitment of DDX18. a. Putative density modeled with cysteine-M12. DCAF11 shown in green, DDX18 shown in purple. b. Chemical structure of M12 analogs. c. Flow cytometry analysis of DDX18-eGFP reporters in K562_cas9_ cells with M12 or its analogs for 18 h. DDX18 stability was calculated as the eGFP/mCherry fluorescence ratio (*n* = 2 technical replicates). d. LC-MS/MS chromatogram of cellular lysates treated with M12 (10 µM) for 1 h. e. Mass spectra of GSH-M12 generated by incubating GST, GSH, and M12. f. Chemical synthesis of HY-05 (Synthetic GSH-M12, see Supplementary Information). g. TR-FRET: Titration of HY-05 (GSH-M12) to biotinylated DCAF11 (100 nM) and GFP-DDX18 (300 nM) in standard buffer condition (*n* = 3 technical replicates). Data are presented as mean values ± s.d. h. TR-FRET: Titration of GFP-DDX18 to biotinylated DCAF11 (100 nM) in the presence of DMSO or GSH-M12 (HY-05, 200 nM) (*n* = 3 technical replicates; data shown is representative of two independent replicates). Data are presented as mean values ± s.d. i. In vitro ubiquitination assay: neddylated CUL4-RBX1 (500 nM) was incubated with DCAF11-DDB1-DDA1 (700 nM) and GFP-DDX18 (1 µM) with buffer, M12 (10 µM), or HY-05(GSH-M12, 10 µM) on ice for 20 min. Reaction was performed in 25 mM HEPES, 100 mM NaCl, 10 mM MgCl_2_, 5 mM ATP, with 2 µM UBE2D, 2 µM UBE2G1, 0.2 µM UBA1, and was initiated by adding 60 µM ubiquitin. A representative image from two independent experiments with similar results is shown.

To understand the molecular basis by which M12 recruits DDX18 to DCAF11 for degradation, we set out to determine a cryo-electron microscopy (cryo-EM) structure of the ternary complex formed by DDX18, M12 and DCAF11 (Fig. 3a and Extended Data Table 1). We co-expressed DDX18, DCAF11 and DDB1∆B (DDB1∆B is DDB1 with deletion of the BPB domain^7^) in insect cells. Following purification of the complex in the presence of M12, cryo-EM grids were prepared and data were collected on a Titan Krios microscope (Thermo), resulting in a consensus map refined to an overall resolution of 2.3 Å (Fig. 3a). A model of the ternary complex was built into density using prior structures of DDB1 (Protein Data Bank (PDB): 5FQD) and AlphaFold2 models of DCAF11 and DDX18. At the interface between DDX18 and DCAF11, we observed additional density that could not be accounted for by DCAF11, DDX18 or M12. This density, contiguous with the density assigned to M12, presented itself as a peptide-like stretch extending beyond the position of M12 that did not belong to DDX18 or DCAF11 (Fig. 3b). The bifurcated and kinked density allowed fitting of a potential cysteine-containing peptide (Extended Data Fig. 3a).

M12 contains a reactive chloride (6-Cl) that faces the observed density and is susceptible to nucleophilic substitution. We generated analogues of M12, and found that removal of the chloride group or attenuation of its reactivity completely abolished M12-induced degradation of DDX18 (Extended Data Fig. 3b,c). We hypothesized that M12 may require a modification at the chloride group to become a functional molecular glue, and that this modification could occupy the unexplained density observed in the cryo-EM structure. In support of this hypothesis, we found that recombinant DDX18 and DCAF11 proteins did not form a complex when incubated with M12 alone. However, when pre-incubated with cellular lysate, we observed M12-dependent complex formation between DCAF11 and DDX18 (Fig. 3c). Similarly, in a time-resolved Förster resonance energy transfer (TR-FRET)-based proximity assay, M12 alone did not induce proximity between DCAF11 and DDX18, but addition of cellular lysate to the assay buffer enabled M12 to promote the DCAF11–DDX18 interaction (half-maximal effective concentration (EC_50_) = 1.76 µM). Boiled cellular lysate did not promote the M12-induced DCAF11–DDX18 interaction (Fig. 3d), indicating that enzymatic activity or another protein factor in the lysate is likely to be required for this process. Together, these findings suggested that an additional component in the lysate is required, along with M12, to form the ternary complex (Fig. 3e).

To determine how M12 was modified, we precipitated the protein components in the DCAF11–DDX18 complex induced by M12 and cellular lysate, and analysed the resulting supernatant using mass spectrometry (Fig. 3f). The mass spectrum revealed a new peak with a distinct elution time and a mass to charge (*m/z*) of 585.1 for the singly charged species, corresponding to a mass difference of 271 Da. This mass difference is consistent with the modification of M12 with glutathione (GSH) accounting for elimination of chloride and a hydrogen, providing strong evidence that the active species is a glutathionylated M12 (GSH-M12) (Fig. 3g). Cells treated with M12 also yielded a metabolite with a mass consistent with GSH-M12 (Extended Data Fig. 3d).

GSH is a tripeptide composed of glutamate, cysteine and glycine, and serves as the substrate for intracellular glutathionylation catalysed by glutathione *S*-transferase (GST). We tested whether the cellular lysate-mediated GSH modification of M12 is GST-dependent and could be recapitulated using purified recombinant GST protein. Incubating M12 with recombinant GST and GSH under buffered conditions yielded the same mass peak as GSH-M12 from the lysate, confirming that GSH modification on M12 is mediated by GST (Extended Data Fig. 3e). Using our TR-FRET assay, we validated that the induced proximity of DDX18 and DCAF11 occurs only in the presence of M12, GST and GSH (EC_50_ = 385 nM) (Fig. 3h). To confirm that no additional modifications of M12 are required, we chemically synthesized GSH-M12 (HY-05) and demonstrated that GSH-M12 promotes DCAF11–DDX18 complex formation without addition of cellular lysate (Extended Data Fig. 3f,g). DDX18 showed no affinity to DCAF11 in the absence of GSH-M12 (Extended Data Fig. 3h). In vitro ubiquitination assays with recombinant proteins further demonstrated GSH-M12-mediated DDX18 ubiquitination by the CRL4^DCAF11^ complex (Extended Data Fig. 3i). The ternary complex cryo-EM map supports GSH modification when GSH-M12 is modelled into the density between DCAF11 and DDX18, providing additional evidence that the GSH modification activates M12 (Fig. 3i). Together, these experiments demonstrate that M12 undergoes conjugation with GSH, enabling M12-dependent DCAF11–DDX18 complex formation (Fig. 3j).

## Structure of GSH-M12-bound DCAF11–DDX18

**Fig. 4:**
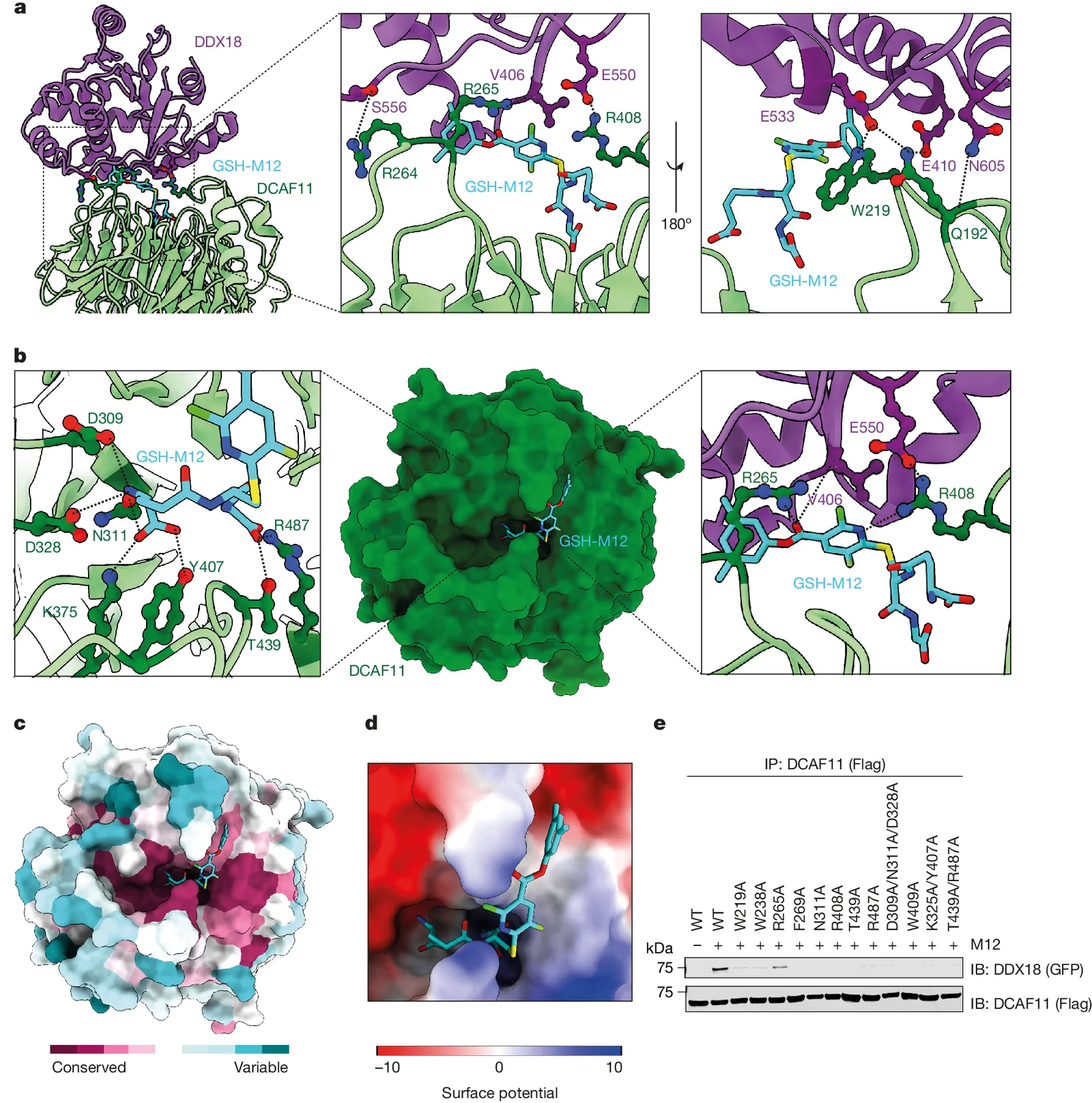
GSH-M12 mediates an interaction network between DCAF11 and DDX18. **a**, Cryo-EM model showing the interaction interface between DCAF11 and DDX18, mediated by GSH-M12. Close-up view of polar contacts between DCAF11 and DDX18. **b**, Surface representation (top view) of modelled DCAF11 (green). Close-up of DCAF11 residues interacting with GSH (left) and the M12 moiety (right). **c**, Conservation representation of the DCAF11 surface (top view)^44^. **d**, Calculated coulombic electrostatic surface potential indicating high charge complementarity between DCAF11 and GSH-M12. Representative images from three independent experiments with similar results. **e**, Immunoprecipitation of wild-type and indicated mutants of DCAF11 in the presence of 10 μM M12.

**Extended Data Fig. 4:**
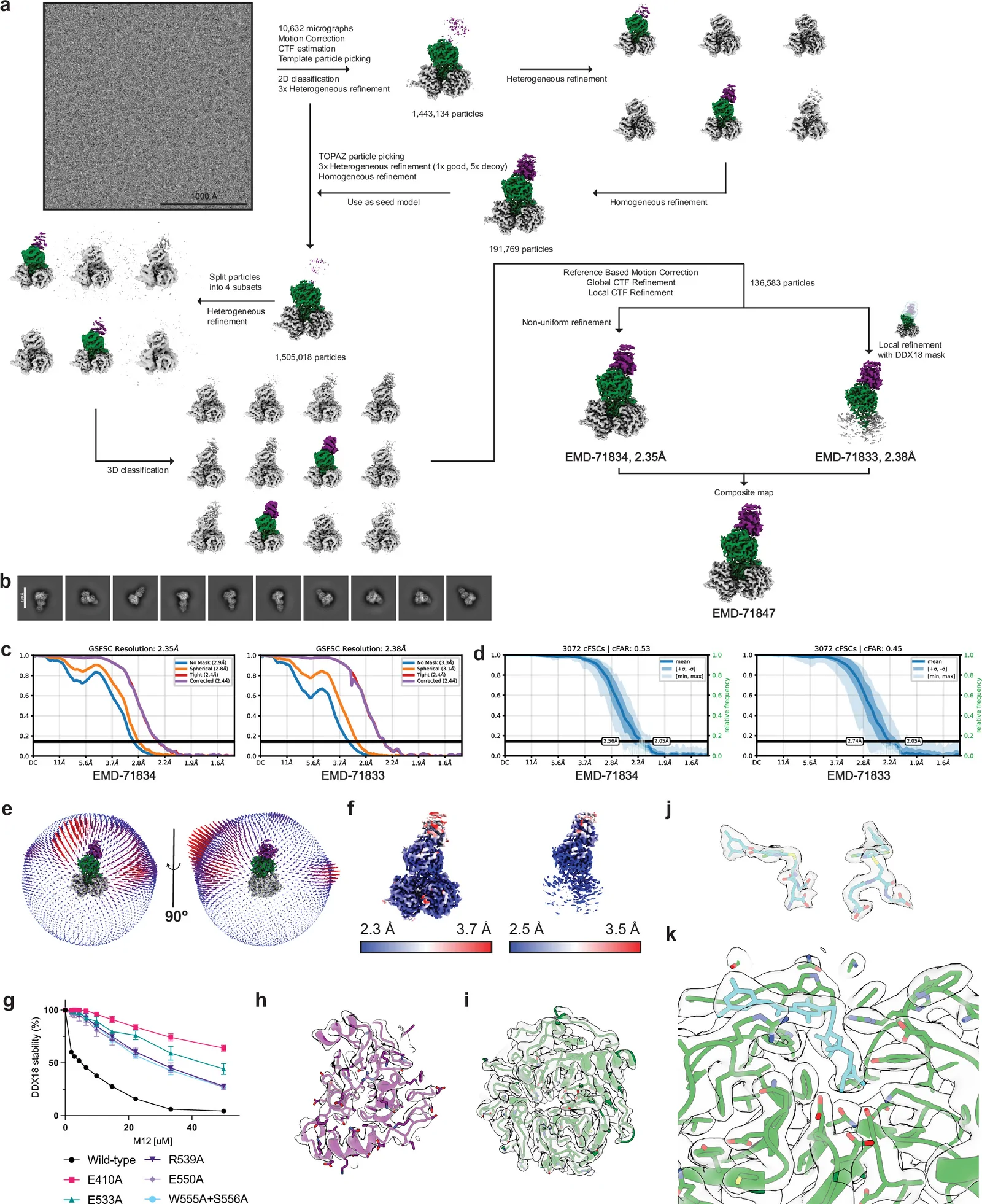
Cryo-EM processing workflow and density examples for DCAF11-DDB1∆B-GSH-M12-DDX18 structure. a. Overview of cryo-EM processing workflow from raw micrograph to final maps. Particles belonging to the colored volumes were used in subsequent steps. b. Representative 2D classes from particles used in the final reconstruction. c. FSC plots for EMD-71834 and EMD-71833. d. Directional FSC plots for EMD-71834 and EMD-71833. e. Viewing distribution for EMD-71834. f. EMD-71834 and EMD-71833 colored according to local resolution. g. Flow cytometry analysis of DDX18 variants-eGFP reporters in K562_cas9_ cells treated with M12 for 18 h. DDX18 stability was calculated as the eGFP/mCherry fluorescence ratio (*n* = 3 technical replicates). Data are presented as mean values ± s.d. h-k. Density examples for DDX18 (h), top view of DCAF11 (i), GSH-M12 (j), and the interaction interface between DCAF11 and GSH-M12 (k).

DDX18 contains two canonical RecA-like domains, referred to as N- and C-terminal lobes^25^. In our cryo-EM reconstruction, only the C-terminal lobe of DDX18 was visible and shown to engage directly with DCAF11 (Extended Data Fig. 4a–h), consistent with the reporter degradation data (Fig. 2c). The 870 Å^2^ interface between DDX18 and DCAF11 is stabilized by a network of polar interactions mediated by V406, E410, E533, E550, S556 and N605 from DDX18 and Q192, W219, R264, R265, R408 from DCAF11 (Fig. 4a and Extended Data Fig. 4i).

The structure provides insight into the requirement of GSH modification for M12 activity. The GSH moiety positions the compound in the protein–protein interface by binding to the canonical central pore of the WD40 β-propeller of DCAF11 (Fig. 4b and Extended Data Fig. 4j,k), which is commonly involved in binding to peptides or ligands. Anchored by GSH, GSH-M12 extends from inside the DCAF11 cavity to the outer groove of the WD40 β-propeller of DCAF11, remodelling the surface of DCAF11 to allow recruitment of DDX18. The M12 moiety of GSH-M12 forms direct polar interactions with DCAF11, including the interactions of R265 in DCAF11 to the carbonyl oxygen of M12 and R408 with the pyrimidine ring contained in M12 (Fig. 4b, right), and this in turn stabilizes a polar interaction network involving R408–D406–W409 in DCAF11. The dimethyl phenyl group of M12 is buried in the interface between DCAF11 and DDX18, stabilizing the overall complex.

**Extended Data Fig. 5:**
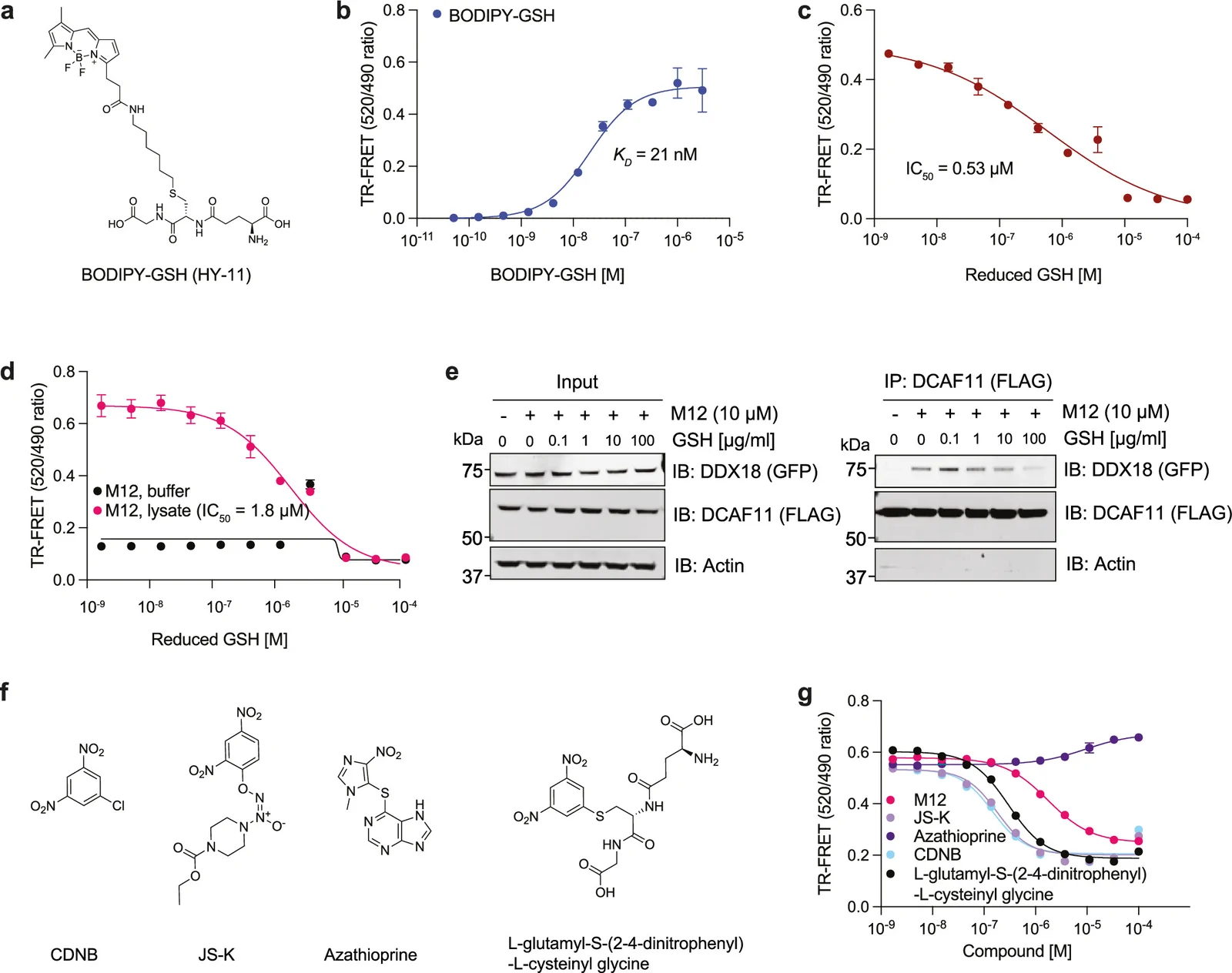
GSH directly binds to DCAF11. a. Chemical structure of BODIPY-labeled GSH (HY-11) b. TR-FRET: Titration of BODIPY-GSH (HY-11) to biotinylated DCAF11 (*n* = 3 technical replicates; data shown is representative of two independent replicates). Data are presented as mean values ± s.d. c. TR-FRET: Pre-assembled complex of 300 nM GFP-DDX18 and 100 nM biotinylated DCAF11 formed by 50 nM GSH-M12 (HY-05) in regular buffer were treated with an increasing concentration of reduced GSH (*n* = 3 technical replicates; data shown is representative of two independent replicates). Data are presented as mean values ± s.d. d. TR-FRET: Pre-assembled complex of 300 nM GFP-DDX18 and 100 nM biotinylated DCAF11 by 200 nM M12 in lysate or buffer conditions were treated with an increasing concentration of reduced GSH (*n* = 3 technical replicates; data shown is representative of two independent replicates). Data are presented as mean values ± s.d. e. Immunoprecipitation: Competitive titration of reduced GSH against the M12-induced complex formed between DCAF11 and DDX18. Representative images from two independent experiments with similar results are shown. f. Chemical structure of the xenobiotic compounds. g. TR-FRET: Pre-assembled 390 nM BODIPY-M12 (HY-06) and 100 nM biotinylated DCAF11 in lysate buffer were treated with M12, xenobiotic compounds (*n* = 3 technical replicates; data shown is representative of two independent replicates). Data are presented as mean values ± s.d.

Notably, the entire GSH-binding site of DCAF11 is highly conserved throughout evolution (Fig. 4c), suggesting a physiological role of DCAF11 in binding to GSH independent of M12. This is further supported by the highly specific network of residues that constitute the interaction between the GSH moiety and DCAF11. Key hydrogen bonds are formed by GSH with D309, N311, D328, K375, Y407, T439, R487 in DCAF11 (Fig. 4b, left). The interaction interface has high charge complementary (Fig. 4d). Disruption of critical interactions through alanine substitutions abrogated M12-mediated recruitment of DDX18 to DCAF11 (Fig. 4e), underscoring the essential role of GSH modification in M12 binding to DCAF11 and DDX18 recruitment. To determine whether GSH directly binds DCAF11, we utilized TR-FRET using BODIPY-labelled GSH (HY-11) and observed its direct binding to DCAF11 (Extended Data Fig. 5a,b). Furthermore, reduced GSH competes with GSH-M12 (Extended Data Fig. 5c), driving dose-dependent dissociation of DDX18 from the GSH-12 mediated DCAF11–DDX18 complex (Extended Data Fig. 5d,e), suggesting that DCAF11 is a principal binder of glutathionylated molecules. In concordance, several xenobiotic compounds that are known to undergo glutathionylation^26–28^ also bind DCAF11 in a dose-dependent manner (Extended Data Fig. 5f). Together, these structural and biochemical results demonstrate that DCAF11 recognizes and binds GSH or glutathionylated molecules, and reveal how GSH-M12 hijacks this evolutionary conserved GSH-binding ligase to facilitate degradation of DDX18.

## Glutathionylation-activated PROTACs

Our biochemical, cellular and structural data revealed that GSH-M12 binds to a highly conserved pocket in DCAF11, which is implicated in substrate recognition. On the basis of this finding, we sought to leverage M12 as a DCAF11-targeting prodrug warhead for proteolysis-targeting chimera (PROTAC) development^29^. Several groups have reported DCAF11-recruiting molecules, establishing DCAF11 as an E3 ligase that can be reprogrammed for targeted protein degradation. Electrophilic PROTACs that covalently modify cysteines in DCAF11 have been shown to induce degradation of targets such as FKBP12, androgen receptor and HDAC, demonstrating that DCAF11 is a ligandable E3 ligase^30,31^. In addition, several Michael acceptor-containing ligands have been reported to enable DCAF11-mediated targeted degradation^32,33^. Beyond classical Michael acceptor warheads, nucleophilic SNAr chemistry has been exploited to develop DCAF11-dependent degraders^34^. Two additional DCAF11-mediated degraders lacking electrophilic moieties have also been reported^35,36^. As these compounds do not contain electrophiles that can be modified by GSH, they are likely to operate through mechanisms that are distinct from glutathionylation-mediated targeted protein degradation.

**Fig. 5:**
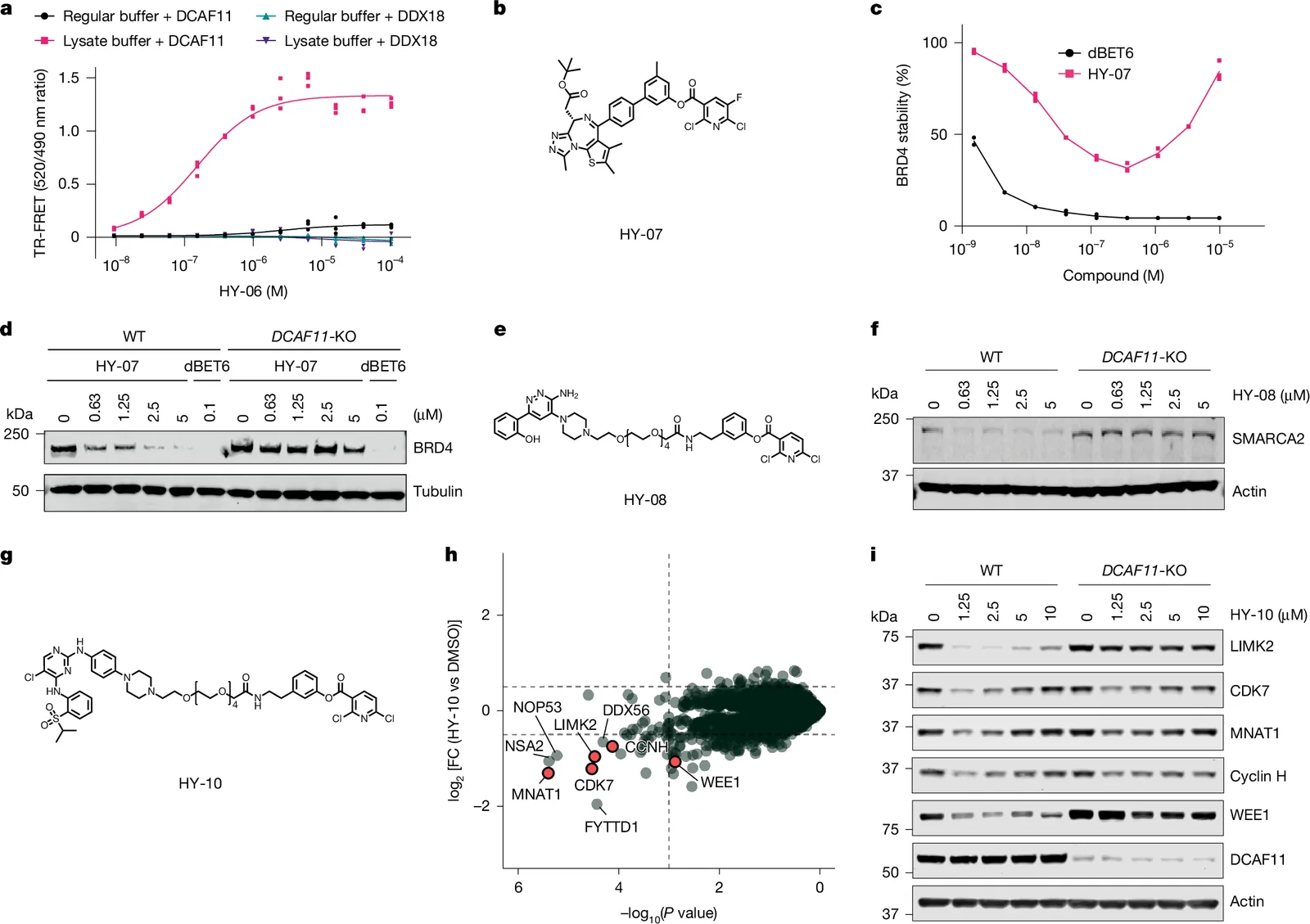
Glutathionylation of M12 confers binding specificity to DCAF11, enabling rational design of DCAF11-based PROTACs. **a**, TR-FRET: Titration of HY-06 with biotinylated DCAF11 (50 nM) or biotinylated DDX18 (200 nM) in standard buffer and cell lysate conditions (*n* = 3 technical replicates; data representative of two independent replicates). **b**, Chemical structure of the DCAF11–BRD4 PROTAC HY-07. **c**, Flow cytometry analysis of BRD4–eGFP reporter in K562 cells treated with HY-07 (*n* = 3 technical replicates) or dBET6 (*n* = 2 technical replicates) for 18 h. BRD4 stability was quantified as the eGFP/mCherry fluorescence ratio. **d**, Immunoblot analysis of BRD4 levels in wild-type and *DCAF11*-KO Jurkat cells treated with increasing concentrations of HY-07 for 18 h. Representative images from two independent experiments with similar results. **e**, Chemical structure of the DCAF11–SMARCA2 PROTAC HY-08. **f**, Immunoblot analysis of SMARCA2 levels in wild-type and *DCAF11*-KO Jurkat cells treated with increasing concentrations of HY-08 for 18 h. Representative images from three independent experiments with similar results. **g**, Chemical structure of the DCAF11–kinase PROTAC HY-10. **h**, Whole-proteome quantification of Molt4 cells treated with 2.5 µM HY-10 (*n* = 3 technical replicates) or DMSO (*n* = 3 technical replicates) for 4 h. Significant changes were assessed by a two-sided moderated *t*-test as implemented in the limma package^43^. **i**, Immunoblot analysis of kinase degradation in wild-type and *DCAF11*-KO cells treated with increasing concentrations of HY-10 for 4 h. Images are from a single experiment.

**Extended Data Fig. 6:**
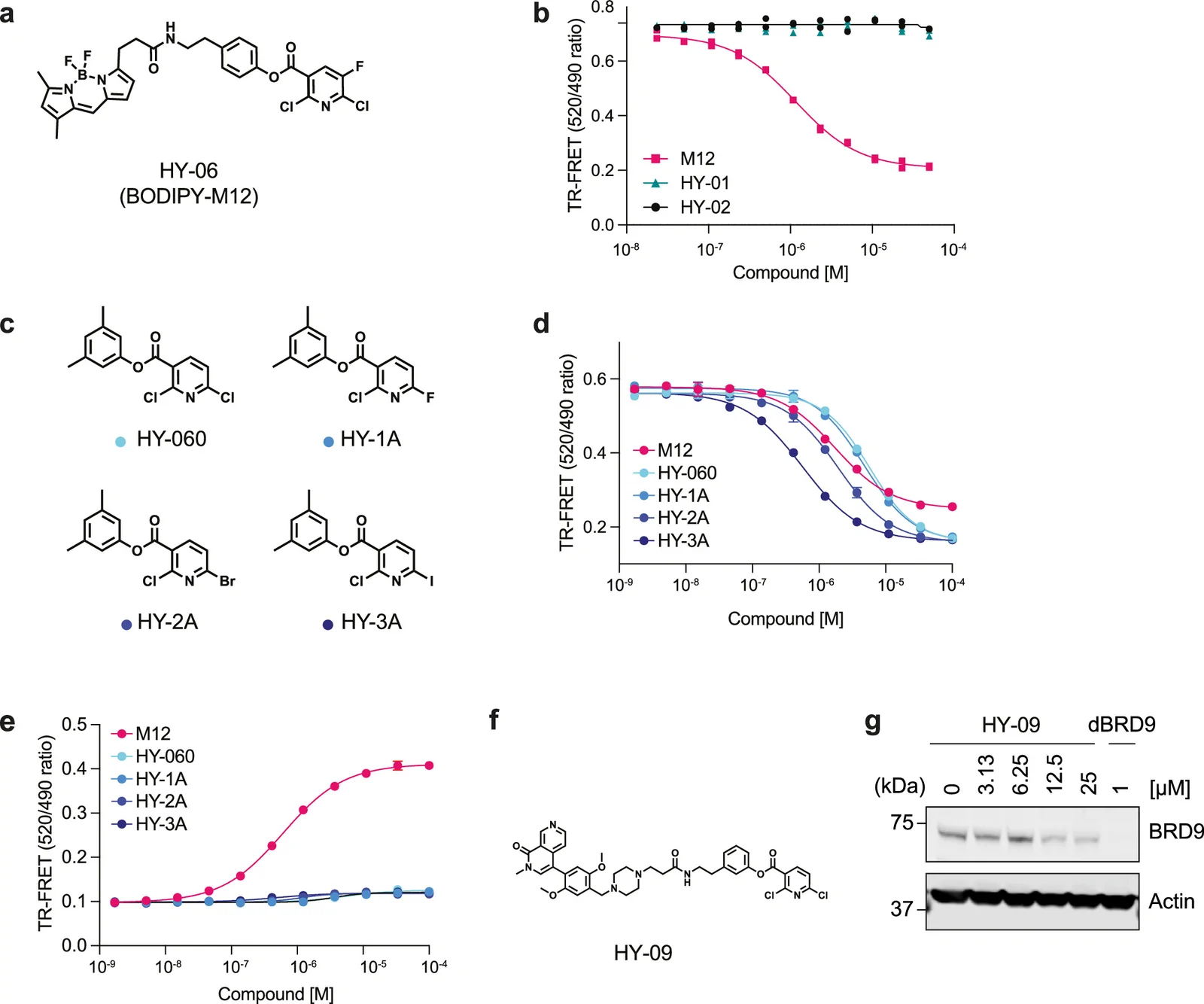
M12 as a DCAF11-binding warhead for the development of PROTACs. a. Chemical structure of BODIPY-labeled M12 (HY-06). b. TR-FRET: Competitive titration of M12 or its analogs against pre-assembled 100 nM HY-06 and biotinylated DCAF11 (200 nM) in lysate buffer (*n* = 2 technical replicates). c. Chemical structure of M12 analogs containing alternative halogen substituents at the 6-position. d. TR-FRET: Competitive titration of M12 or its analogs against pre-assembled 390 nM HY-06 and biotinylated DCAF11 (100 nM) in lysate buffer (*n* = 3 technical replicates; data shown is representative of two independent replicates). Data are presented as mean values ± s.d. e. TR-FRET: Titration of M12 or its analogs to biotinylated DCAF11 (100 nM) and GFP-DDX18 (300 nM) in lysate buffer (*n* = 3 technical replicates). Data are presented as mean values ± s.d. f. Chemical structure of DCAF11-BRD9 PROTAC (HY-09). g. Immunoblot analysis of BRD9 levels in Jurkat cells treated with HY-09 or dBRD9 for 18 h.

Using a BODIPY-labelled M12 (HY-06) (Extended Data Fig. 6a), we measured its binding affinity to DCAF11 and observed strong affinity (apparent dissociation constant (*K*_d_) = 149 nM) only in the presence of cellular lysate, and no detectable affinity was found with DDX18 (Fig. 5a). Consistent with analysis of M12 derivatives using the reporter-based DDX18 degradation (Extended Data Fig. 3b,c), the binding of M12 to DCAF11 was dependent on the reactive Cl group that was required for GSH modification, further confirming its glutathionylation-mediated interaction with DCAF11, as neither the phenyl derivative (HY-01) nor the de-chlorinated analogue (HY-02) exhibited measurable binding (Extended Data Fig. 6b). Analogues without the 5-F but bearing alternative halogen substitutions at the 6 position can still bind to DCAF11, but do not mediate DDX18 recruitment (Extended Data Fig. 6c–e)

We linked M12 to the BET bromodomain inhibitor JQ1 to generate HY-07, and found that this molecule efficiently degrades BRD4 (Fig. 5b,c). DCAF11 knockout abolished compound-induced BRD4 degradation, confirming the dependency on DCAF11 (Fig. 5d). Next, we conjugated M12 to a SMARCA2 ligand to generate HY-08, and found that this molecule induced robust degradation of SMARCA2. Similarly, linking M12 to a BRD9 inhibitor produced HY-09, which triggered dose-dependent degradation of BRD9, albeit with lower potency compared to other degraders (Extended Data Fig. 6f,g). To explore the broader applicability of M12 as a DCAF11-recruiting warhead, we synthesized the multikinase-targeting PROTAC HY-10 by linking M12 to a promiscuous kinase inhibitor^37^ (Fig. 5h). Quantitative proteomics revealed that HY-10 induced degradation of several kinases—LIMK2, WEE1 and CDK7–cyclin H/MAT1 complex—all of which were further validated by western blot analysis (Fig. 5i). Collectively, our finding demonstrate that M12 can be readily repurposed as a modular warhead to reprogramme DCAF11 towards diverse neo-substrates, highlighting its potential for expanding the scope of DCAF11-mediated targeted degradation.

## Discussion

Discovery of molecular glue degraders that co-opt E3 ligases beyond a few well-characterized E3 ligases remains challenging, as it requires unbiased strategies to identify compounds that induce proximity between an E3 ligase and a neo-substrate without prior knowledge of their interaction while sampling vast chemical space. Here we present a novel strategy to greatly increase the search space by simultaneously multiplexing ligases and compounds in a cellular lysate-based IP–MS screen. Using this platform, we discovered M12, which recruits DDX18 to DCAF11 for targeted protein degradation. Structural studies along with comprehensive biochemical dissection reveal that M12 undergoes glutathionylation by GST in cells, converting it into a functional molecular glue. The conjugated GSH moiety facilitates direct binding to DCAF11, thereby promoting the recruitment of DDX18. Additionally, GSH-M12 binds with high affinity to a pocket located within the canonical substrate recognition site of WD40 propeller substrate receptors, enabling its conversion to bifunctional degraders. These findings uncover a novel mechanism of targeted protein degradation, in which a small molecule prodrug acquires neo-morphic molecular glue activity by co-opting a specific, intracellular enzymatic modification.

GSH is a key regulator of redox homeostasis, and functions in maintaining cellular redox balance and protecting against oxidative stress^38^. Distinct from its known functions, the GST- and GSH-mediated gain-of-function activities described here represent, to our knowledge, the first known example of enzyme-mediated activation into a functional molecular glue. Given that many cancer cells maintain high levels of GSH to protect against reactive oxygen species while actively proliferating^39^, it is conceivable that GSH-mediated activation of a prodrug may enhance its therapeutic index. Future investigation into GSH and GST levels across cancer cells and correlation with M12 cytotoxicity could lead to advances in the development of antitumour agents with improved selectivity.

Our studies reveal that the GSH-binding pocket of DCAF11 is highly conserved and that GSH itself can directly bind to DCAF11, suggesting a physiological GST- and GSH-dependent regulatory mechanism involving DCAF11. Given the diversity of naturally glutathionylated species, DCAF11 may recognize a wide range of glutathionylated substances—either endogenous metabolites or proteins—in a context-dependent manner, which is analogous to other quality control pathways that respond to post-translational modifications, cleavage or cyclization to trigger protein degradation^40–42^. Considering the conserved and modular nature of substrate recognition domains across the E3 ligase family, other mechanisms similar to GSH-dependent DCAF11 regulation may exist for other E3 ligases.

Although molecular glue degraders hold considerable therapeutic promise, their discovery and application remain largely confined to a few E3 ligases. Here, we demonstrate that harnessing an endogenous enzymatic modification pathway with a small molecule can reprogramme an E3 ligase. Glutathionylation of reactive small molecules leading to DCAF11-mediated ubiquitination offers an opportunity to widen the scope of targeted protein degradation. More broadly, our multiplexed approach to identification of molecular glue degraders opens new possibilities to expand the repertoire of E3 ligases that can be redirected for targeted degradation of neo-substrates.

## Methods

### Compounds

dBET6 (HY-112588), dBRD9 (HY-117690), MLN7243 (HY-100487), MLN4924 (HY-70062), MG132 (HY-13259), bafilomycin A_1_ (HY-100558), and 5K Scaffold Library (HY-L902) were obtained from MedChemExpress.

### Plasmids

The following plasmids were used in this study: Artichoke (Addgene #73320) and Cilantro (Addgene #74450) for flow-based reporter degradation assays, reporter CRISPR screens and co-immunoprecipitation; sgBFP (U6.sgRNA.SFFV.tBFP) for validation of *DCAF11*-knockout phenotypes; pNTM2 (CMV) for co-immunoprecipitation; pAC8-derived plasmids for protein purification. All proteins are derived from human origin sequences: full-length DCAF11, DDX18 (residues 171–625), DDB1(ΔBPB) (residues 1–395 and 706–1140 with a GNGNSG linker) and full-length DDA1. Unique amino acid tags: Flag (for DCAF11, DDX18), Flag–GFP (for DDX18), StrepII–Avi (for DCAF11 and DDX18) and His (for DDB1(ΔBPB) and DDA1) were designed to the N-terminal ends of the constructs, and then subcloned into pAC-derived expression vectors (pAC8RedNK)^45^.

### Antibodies

The following antibodies were used: Flag (CST 14793S), GFP (CST 2555S), β-actin (CST 3700S), tubulin (Sigma T9026), BRD4 (Bethyl A301-985A-T), DDX18 (GeneTex GTX103392), BRD9 (Bethyl, A303-781A-T), SMARCA2 (Bethyl A301-015A-T), LIMK2 (CST 3845 T), WEE1 (CST 4936S), CDK7 (Proteintech 27027-1-AP), cyclin H (Proteintech 67065-1-lg), MNAT1 (Proteintech 11719-1-AP), DCAF11 (Novus Biologicals NBP2-92244), IRDye 800CW Goat anti-Rabbit IgG Secondary Antibody (LI-COR 926-32211) and IRDye 680LT Goat anti-Rabbit IgG Secondary Antibody (LI-COR 925-68021).

### Protein expression and purification

The recombinant proteins from the constructs in pAC-derived vectors were expressed in *Trichoplusia ni* High Five insect cells (Gibco, 85502) using the baculovirus expression system. In brief, expression plasmids were transfected into *Spodoptera frugiperda* (Sf9) cells (Expression Systems, 94-001 F) at a density of 0.9 × 10^6^ cells per ml grown in ESF 921 medium (Expression Systems) to generate baculovirus, and this was followed by 2 rounds of infection in Sf9 cells to increase viral titre. For recombinant protein expression, High Five cells grown in SF-4 baculo express insect medium (BioConcept) at a density of 2.0 × 10^6^ cells per ml were infected with baculovirus at 1.5% v/v ratio. After 42 h of expression at 27 °C, High Five cells were collected by centrifugation for 15 min at 1,500 rpm. For purification of StrepII or Flag-tagged proteins, pelleted cells were resuspended in lysis buffer containing 50 mM Tris (hydroxymethyl) aminomethane hydrochloride (Tris-HCl) pH 8.0, 200 mM NaCl, 1 mM Tris (2-carboxyethyl) phosphine (TCEP), and protease inhibitors, and the cell pellets were lysed by sonication. After ultracentrifugation (1 h, 40,000 rpm, 4 °C), the soluble fraction was passed over the appropriate affinity resin of Strep-Tactin XT Superflow (IBA 2-4010-025) or Anti-DYKDDDDK G1 Affinity Resin (Genscript L00432), eluted with wash buffer (50 mM Tris-HCl pH 8.0, 200 mM NaCl, 1 mM TCEP) supplemented with 50 mM Biotin (IBA 2-1016-005) or 0.15 mg ml^−1^ Flag peptide (custom synthesis), respectively. The affinity-purified proteins were then applied to an ion exchange column (POROS 50HQ, Thermo Scientific 1255911) and eluted in 50 mM Tris-HCl pH 8.5 and 2 mM TCEP by a linear salt gradient (from 50 mM to 1000 mM NaCl). All proteins were then subjected to size-exclusion chromatography on a Superdex 200 Increase 10/300 (Cytiva 28990944) in 25 mM 4-(2-hydroxyethyl)-1-piperazineethanesulfonic acid (HEPES) pH 7.4 or pH 8.0, 150 mM NaCl and 1 mM TCEP. For structural biology studies, the non-concentrated peak fraction was used, whereas for biochemistry studies, protein within the peak was pooled, concentrated, and flash frozen in liquid nitrogen and stored at −80 °C.

### Biotinylation

Purified StrepII–Avi-tagged DCAF11 or DDX18 were biotinylated in vitro by incubation with final concentrations of 2.5 μM BirA enzyme (prepared in-house) and 0.2 mM biotin in 50 mM HEPES, pH 7.4, 200 mM NaCl, 10 mM MgCl_2_, 1 mM TCEP and 20 mM ATP. The reaction was incubated for 1 h at room temperature and stored overnight at 4 °C. Biotinylated proteins were purified by size-exclusion chromatography and flash frozen in liquid nitrogen and stored at −80 °C.

### TR-FRET

Titrations of compounds to induce the DCAF11–DDX18 complex were carried out by mixing biotinylated DCAF11 and GFP–DDX18 at the concentration described in the figure legend, and 2 nM terbium-coupled streptavidin (prepared in-house) in an assay buffer containing 50 mM HEPES pH 8.0, 200 mM NaCl, 1 mM TCEP, 0.05% Tween-20, and 1 mM TCEP. For lysate buffer, 2 × 10^7^ HEK293T cells were lysed by sonication. After centrifugation (15,000 rpm, 30 min, 4 °C), the soluble fraction was used as a lysate buffer. For GST and GSH-mediated TR-FRET, 0.5 mg ml^−1^ GST and 0.2 mg ml^−1^ of reduced GSH were used. After dispensing the assay mixture (15 μl volume), increasing concentrations of compounds were dispensed in a 384-well microplate (Corning 4514) using a D300e Digital Dispenser (HP) and then incubated for 1 h at room temperature. After excitation of terbium fluorescence at 337 nm, emission at 490 nm (terbium) and 520 nm (GFP) were recorded with a 70-μs delay over 600 μs to reduce background fluorescence, and the reaction was followed over 60 cycles of each data point using a PHERAstar FS microplate reader (BMG Labtech). The TR-FRET signal of each data point was extracted by calculating the 520/490 nm ratio. The dose-dependent TR-FRET curve was generated using GraphPad Prism. The number of technical replicates is indicated in the figure legend.

Titrations of BODIPY-labelled M12, BODIPY-labelled GSH or GFP–DDX18 were carried out by mixing biotinylated DCAF11 (or biotinylated DDX18) at the concentration described in the figure legend, and 2 nM terbium-coupled streptavidin in the same assay buffer or lysate buffer. After dispensing the assay mixture, an increasing concentration of BODIPY-M12 was dispensed in the 384-well plate using a D300e Digital Dispenser then incubated for 1 h at room temperature. The 520/490 nm ratios from the sample with biotinylated proteins were subtracted by the ratios from the sample without proteins, and the subtracted values were plotted using GraphPad Prism. The number of technical replicates is indicated in the figure legend.

### Multiplexed immunoprecipitation and sample preparation for mass spectrometry analysis

DMSO, CC-885 (positive control, 31.25 µM, 6 µl per well) or pools of 320 drugs (31.25 µM, 6 µl per well) were dispensed into a 96-well plate in triplicate. A total of 1 × 10^9^ frozen cells (8 × 10^8^ Expi293, 5 × 10^7^ K562, 1 × 10^8^ U937 and 5 × 10^7^ Hep3B cells) were resuspended in 20 ml lysis buffer (50 mM Tris pH 8.0, 200 mM NaCl, 2 mM TCEP, 0.1% NP-40, 2 µl Benzonase (EMD Millipore 77664-3), and 1 tablet of cOmplete protease inhibitor cocktail). The suspension was sonicated on ice for 20 cycles (3 s on, 5 s off) at 25% amplitude. After centrifugation, 504 µl of each of 7 bait proteins (15 µM each) were added to 9.5 ml of clarified lysate. Then, 125 µl of the lysate–protein mixture was added to each well of the pre-plated 96-well PCR plate containing compounds. The plate was incubated on ice for 1 h, followed by addition of 50 µl pre-washed MagStrep Strep-Tactin XT beads (IBA 2-5090-010) to each well (final composition: 90 µl lysate, 6 µl compound pool, 25 µl protein, 50 µl resin; final concentration of each drug: 1.1 µM). The mixture was incubated for an additional hour on ice. Beads were washed using wash buffer (50 mM Tris pH 8.0, 2 mM TCEP) containing the corresponding compound pool, and elution was performed using 0.5 M NaOH. The eluate was immediately neutralized with 0.1 M Tris pH 2.0 to achieve a final pH of 8.5 and a final volume of 200 µl.

Samples were reduced with TCEP (final concentration 10 mM) for 30 min at room temperature on a thermomixer, followed by alkylation with iodoacetamide (Sigma I1149, final concentration 15 mM) for 45 min, protected from light. The reaction was quenched with 1 M DTT (final concentration 10 mM). Subsequently, proteins were digested with 3 µg of Trypsin/Lys-C Mix, Mass Spec Grade (Promega V5072) overnight at 37 °C. Sample digests were acidified with formic acid to a pH of 2–3 prior to desalting using C18 solid phase extraction plates (Thermo Scientific 60307). Desalted peptides were dried in a vacuum-centrifuged and reconstituted in 0.1% formic acid for LC–MS analysis. Data for multiplexed immunoprecipitations were collected following the DDA methods described below. Data for non-multiplexed immunoprecipitations were collected following the diaPASEF methods described below.

### Sample preparation for whole-cell quantitative proteomics

Treated cells were lysed by addition of lysis buffer (8 M urea, 50 mM NaCl, 50 mM 4-(2-hydroxyethyl)-1-piperazineethanesulfonic acid (EPPS) pH 8.5, protease and phosphatase inhibitors) and homogenization by bead beating (BioSpec) for three repeats of 30 s at 2,400 strokes per min. Protein quantification, tryptic digestion and C18 desalt was performed following procedures described^46^. Data for whole-cell quantitative proteomics were collected following the diaPASEF methods described below.

### LC–MS data collection and analysis using diaPASEF

Data were collected on a TimsTOF HT (Bruker Daltonics) coupled to a nanoElute2 LC pump (Bruker Daltonics) as described^19,46^. The diaPASEF raw file processing and false discovery rate analysis was performed using library free analysis in DIA-NN^47^ searched against a Swiss-Prot human database (January 2021) using the default settings for directDIA, which include the following: tryptic with two missed cleavages, carbamidomethylation of cysteine, and oxidation of methionine and precursor Q-value (false discovery rate) cut-off of 0.01. Precursor quantification strategy was set to Robust LC (high accuracy) with retention time-dependent cross run normalization.

For global proteomics, proteins with low sum of abundance (<2,000× no. of treatments) were excluded from further analysis and resulting data was filtered to only include proteins that had a minimum of 3 counts in at least 3–4 replicates of each independent comparison of treatment sample to the DMSO control.

For multiplexed immunoprecipitation proteomics: Resulting data was filtered to only include proteins that had a minimum of three precursor counts in at least four replicates of each independent comparison of treatment sample to the DMSO control. Protein abundances were globally normalized using in-house scripts in the R framework.

Proteins with missing values were imputed by random selection from a Gaussian distribution either with a mean of the non-missing values for that treatment group or with a mean equal to the median of the background (in cases when all values for a treatment group are missing). Significant changes comparing the relative protein abundance of the treatment to DMSO control comparisons were assessed by two-sided moderated *t*-test as implemented in the limma package within the R framework^43^.

### LC–MS data collection and analysis using DDA

Data were collected using an Orbitrap Exploris 480 mass spectrometer (Thermo Fisher Scientific) coupled with an UltiMate 3000 RSLCnano System. Peptides were separated on an Aurora 25 cm × 75 μm inner diameter microcapillary column (IonOpticks), and using a 60 min gradient of 5–25% acetonitrile in 1.0% formic acid with a flow rate of 250 nl min^−1^. Each analysis used a TopN data-dependent method. The data were acquired using a mass range of *m*/*z* 350–1,200, resolution 60,000, 300% normalized AGC target, auto maximum injection time, dynamic exclusion of 30 s, and charge states of 2–6. TopN 40 data-dependent MS2 spectra were acquired with a scan range starting at 110 *m*/*z*, resolution 15,000, isolation window of 1.4 *m*/*z*, HCD normalized collision energy set at 30%, standard AGC target and the automatic maximum injection time.

Data were collected using an Orbitrap Eclipse mass spectrometer (Thermo Fisher Scientific) coupled with an UltiMate 3000 RSLCnano System. Peptides were separated on a 50 cm, 75 μm inner diameter EasySpray ES903 microcapillary column (Thermo Fisher Scientific), and using a 60 min gradient of 6–22% acetonitrile or a 60 min gradient of 5–25% acetonitrile in 1.0% formic acid with a flow rate of 350 nl min^−1^. Each analysis used a cycle time-based data-dependent acquisition method with a total cycle time of 3 s. Full MS1 scans were acquired in the orbitrap at a resolution of 120,000 over a mass range of *m*/*z* 375–1,325, with a maximum injection time of 200 ms and AGC target of 4 × 10^5^ (normalized 100%). Precursors with charge states of 2–6 and intensity above 20,000 were selected for fragmentation, with 30 s dynamic exclusion. MS1 scans were acquired in the orbitrap at a resolution of 30,000 using HCD fragmentation with a normalized collision energy of 35%, quadrupole isolation window of 0.5 *m*/*z* and 54 ms maximum injection time. The AGC target for MS2 scans was 5 × 10^4^ (normalized 100%), and data were collected in centroid mode.

Proteome Discoverer 2.4 or 2.5 (Thermo Fisher Scientific) was used for processing of RAW files and controlling peptide and protein level false discovery rates, assembling proteins from peptides, and protein quantification from peptides. MS/MS spectra were searched against a Swiss-Prot human database (January 2021) with both the forward and reverse sequences, as well as with known contaminants, such as human keratins. Database search criteria were as follows: tryptic with two missed cleavages, a precursor mass tolerance of 10 ppm, fragment ion mass tolerance of 0.03 or 0.06 Da, static alkylation of cysteine (57.0215 Da) and variable oxidation of methionine (15.9949 Da), N-terminal acetylation (42.0106 Da) and with or without phosphorylation of serine, threonine and tyrosine (75.966 Da). Peptides were quantified using the MS1 Intensity, and peptide abundance values were summed to yield the protein abundance values. Resulting data was filtered to only include proteins that had a minimum of 2 counts in at least 2 replicates of each independent comparison of treatment sample to the DMSO control. Protein abundances were globally normalized using in-house scripts in the R framework. Proteins with missing values were imputed by random selection from a Gaussian distribution either with a mean of the non-missing values for that treatment group or with a mean equal to the median of the background (in cases when all values for a treatment group are missing). Significant changes comparing the relative protein abundance of the treatment to DMSO control comparisons were assessed by two-sided moderated *t*-test as implemented in the limma package within the R framework^43^.

### Quantitative PCR

A total of 1 × 10^6^ Jurkat cells were treated with DMSO or 20 µM M12 for 18 h, collected by centrifugation, washed with phosphate-buffered saline (PBS) and flash frozen in −80 °C. mRNA was isolated using the QIAGEN RNeasy Plus (Qiagen 74134). For cDNA synthesis, 1 µg of RNA was reverse-transcribed with iScript cDNA Synthesis Kit (Bio-Rad, 1708891) and before quantitative PCR analysis with TaqMan Gene Expression Master Mix (Applied Biosystems 4369016) for *DDX18* (Life Technologies TaqMan Hs00705691_s1) and *GAPDH* (TaqMan, Hs02786624_g1). Reactions were run and analysed on the QStudio 6 FLX Real-Time PCR System (Applied Biosystems). Fold expression was calculated using the ∆∆Cq method by normalizing cycle threshold (Cq) values to the GAPDH reference gene and the M12-treated sample to the DMSO sample.

### Immunoblots

Cells were washed with PBS and lysed in RIPA lysis buffer (Thermo Scientific 89901) with cOmplete Protease Inhibitor Cocktail (Sigma 11836170001) for 20 min on ice. The insoluble fraction was removed by centrifugation; the protein concentration was quantified using a BCA protein assay kit (Thermo Scientific 23227); and an equal amount of lysate was run on SDS–PAGE 4–12% Bis-Tris protein gels (Thermo Scientific) and then transferred to nitrocellulose membrane with an XCell II Blot Module Wet Tank Transfer System (Thermo Scientific). Membranes were blocked in Intercept (PBS) Blocking Buffer (LI-COR Biosciences 927-70001) and incubated with primary antibodies overnight at 4 °C. The membranes were then washed in Tris-buffered saline with Tween-20 (TBS-T), incubated for 1 h with secondary IRDye-conjugated antibodies (LI-COR Biosciences) and washed three times in TBS-T for 5 min before near-infrared western blot detection on an Odyssey Imaging System with Image Studio software (LI-COR Biosciences).

### Co-immunoprecipitation

Five million HEK293T cells expressing DDX18–eGFP in Cilantro were plated in 10 cm dishes. After 1 day, cells were transiently transfected with 5 µg of Flag-tagged DCAF11 wild-type or mutants in pNTM plasmid using TransIT-LT1 (Mirus MIR 2304). After an additional 1 day of incubation, cells were treated with MLN4924 (1 µM) for 1 h, then subsequently treated with M12 (10 µM) for 6 h. After the incubation period, cells were collected, washed with PBS, and lysed in Pierce IP lysis buffer (Thermo Scientific 87787) supplemented with cOmplete protease inhibitor cocktail. Cells were lysed for 30 min on ice, with vortexing every 10 min, then centrifuged for 10 min to remove the insoluble fraction. M12 was added to the wash buffer and lysis buffer of M12-treated samples. After being washed in IP lysis buffer, 25 µl of Anti-DYKDDDDK Magnetic Agarose (Thermo Scientific A36797) was added to each lysate sample. Samples were then incubated at 4 °C overnight on a rotator. Beads were washed three times with IP lysis buffer, and then boiled in 1× NuPage LDS sample buffer (Invitrogen NP0007). Immunoblotting was performed using the procedure described in the ‘Immunoblots’ section above.

### Reporter cell line generation

Reporter constructs were transformed into Stbl3 *Escherichia coli* and purified using a MiniPrep Kit (Invitrogen K210011), and sequences were confirmed by Sanger sequencing (Quintara Biosciences). Lentiviruses for reporters were packaged into lentivirus as follows. First, 0.55 × 10^6^ HEK293T cells were seeded in 2 ml of DMEM medium. The next day, a packaging mix that includes 1.5 μg of psPAX2, 0.15 μg of pVSV-G and 1.5 μg of transgene plasmid was prepared in 37.5 µl of OptiMEM (Gibco 31985070). This mix was combined with 9 μl of TransIT-LT1 and 15 µl of OptiMEM, incubated for 30 min at room temperature and then applied dropwise to cells. Cells were allowed to incubate for another 48 h. Lentivirus was collected by 0.4-μm filters and then transduced to 1 × 10^6^ of HEK293T-Cas9 or K562-Cas9 cells at 10% volume ratio by spin infection. One day after infection, reporter cells were selected with puromycin at a concentration of 2 μg ml^−1^.

### Reporter degradation assays

K562-Cas9 cells stably expressing DDX18 or BRD4 reporters were dosed with DMSO or degraders at various times and concentrations using D300e Digital Dispenser (HP). The fluorescent signal was quantified by flow cytometry (Symphony flow cytometer with BD FACSDiva 8.0 software, BD Biosciences, see Supplementary Fig. 2a for the gating strategy) and analysed using FlowJo v10 (flow cytometry analysis software, BD Biosciences). The geometric mean of the eGFP and mCherry fluorescent signal for round and mCherry-positive cells was calculated. GFP expression was normalized to mCherry signal, and drug treatments were compared to DMSO controls. The number of technical replicates is indicated in the figure legend. Data are shown from one representative experiment.

### Genome-scale or UPS-targeted DDX18 reporter CRISPR screen

The genome-scale (Brunello sgRNA library; Addgene, #73178) or UPS-targeted CRISPR library (BISON sgRNA library; Addgene #169942) containing viruses were spin infected into K562-Cas9 cells expressing DDX18–eGFP stability reporter at a 10% volume ratio. Transduced cells were allowed to recover and expand for 7 days (Brunello) and 14 days (Bison) and then treated with DMSO or 10 µM M12 for 18 h. Top (stable gate) and bottom (unstable gate) 5% of cells by eGFP/mCherry fluorescence ratios were sorted for two replicates (see Supplementary Fig. 2b for the gating strategy). Sorted cells were pelleted and lysed, and sgRNAs were amplified, quantified by next-generation sequencing and analysed for enrichment in stable gate over unstable gate, representing degradation rescue. The resulting data was analysed as described previously^48^, using R (v4.5.1) and RStudio (v2025.05.1+513) with the following packages: tidyverse (v2.0.0), ggrepel (v0.9.8), GGally (v2.4.0), dr4pl (v2.0.0) and ShortReads (Bioconductor v3.2.4).

### Single-gene knockouts

Guide RNAs targeting genes of interest were cloned into the sgBFP vector using BsmBI digestion/ligation as previously described^48^. Lentivirus was produced as described above. DDX18 stability reporter in K562-Cas9 cells were transduced with sgRNAs. The effect of the knockdown was determined by quantifying the GFP/mCherry ratios in BFP positive and negative populations by flow cytometry seven days after infection. Guid RNAs: sgDCAF11 #1, TGTGGGATCGACGCACCATG; sgDCAF11 #2, CGCCTAGATTGAGTCCCATG; sgDCAF11 #3, AGACGCTCCAGCCTACGTCG; and sgDCAF11 #4, AGAGGGTAAGTTACCTGCGG.

### In vitro reconstitution of GSH-M12

A frozen aliquot (500 µl, 2.5 mg ml^−1^) of the complex DCAF11, DDB1(ΔBPB), DDA1 and DDX18 (residues 171–625), formed by 10 µM M12, were thawed, and an equal volume of acetonitrile (500 µl) was added to precipitate the proteins. The resulting opaque mixture was centrifuged at 15,000 rpm for 10 min at 4 °C. The supernatant was then analysed using UPLC–MS/MS (Waters) to obtain low-resolution (unit-mass) mass spectrometric data.

### In vitro glutathionylation on M12 by GSH and GST

M12 (1 µM), GST (0.5 mg ml^−1^, purified in-house), and GSH (0.2 mg ml^−1^, reduced, Sigma G4251) were incubated in 100 µl buffer (50 mM Tris pH 8.0, 200 mM NaCl) at room temperature for 30 min, and an equal volume of acetonitrile was added to precipitate the proteins. The resulting opaque mixture was centrifuged at 15,000 rpm for 10 min at 4 °C. The supernatant was then analysed using UPLC–MS/MS (Waters) to obtain low-resolution (unit-mass) mass spectrometric data.

### LC–MS/MS quantification of GSH-M12 in cell media and cell lysates

The concentrations of GSH-M12 in cell media and cell lysate samples were determined using a validated LC–MS/MS bioanalytical method. For sample preparation, a 50 µl aliquot of each sample was mixed with 50 µl of methanol/water (80:20, v/v). The mixture was vortexed for 10 min and centrifuged at 4,000 rpm for 10 min at 4 °C prior to LC–MS/MS injection. Calibration standards and quality control samples were prepared in the corresponding pooled DMSO-treated cell medium or lysate, respectively. The LC–MS/MS system consisted of a Shimadzu Nexera X2 UHPLC system coupled with a Sciex 5500 triple quadrupole mass spectrometer (ESI+). The optimized source parameters were as follows: ion source gas 1 (GS1), 55 psi; ion source gas 2 (GS2), 55 psi; curtain gas (CUR), 30 psi; collision gas (CAD), 9 psi; source temperature, 550 °C; and ion spray voltage, 4,000 V.

Chromatographic separation of GSH-M12 was achieved on a Supelco Ascentis Express C18 column (2.1 × 30 mm, 2.7 µm, 90 Å) using a gradient elution. Mobile phase A was 5 mM ammonium acetate in water with 1% (v/v) formic acid, and mobile phase B was 1 mM ammonium acetate in acetonitrile/water (95:5, v/v) with 0.025% formic acid. The liquid chromatography gradient (%B) was 1% (0.00–0.30 min), 1–95% (0.30–1.40 min), 95% (1.41–2.00 min), 95-1% (2.00–2.01 min) and 1% (2.01–2.40 min). The flow rate was 0.5 ml min^−1^. The column temperature was 40 °C and the injection volume was 5 µl. GSH-M12 was detected by the multiple reaction monitoring (MRM) transition at *m*/*z* 585.022 > 456.026. Under these conditions, the retention time of GSH-M12 was 1.35 min. The method was validated over a concentration range of 0.5 to 500 ng ml^−1^ for GSH-M12 in the cell medium or cell lysate.

### Metabolite identification of M12 in cell lysates

Metabolites of M12 in cell lysates were analysed using a high-resolution mass spectrometer (HRMS). For sample preparation, a 50 µl aliquot of each sample was mixed with 50 µl methanol. The mixture was vortexed then centrifuged at 12,000 rpm for 10 min at 4 °C prior to LC–MS injection. The corresponding DMSO-treated cell lysate samples were used as negative controls. HRMS was performed using a Thermo Vanquish Horizon UHPLC system coupled with a Thermo LTQ Orbitrap Elite high-resolution mass spectrometer (ESI+). The source conditions were as follows: heat temp 375 °C, sheath gas flow rate 45, aux gas flow rate 10, sweep gas flow rate 3, I spray voltage 4.10 kV, capillary temperature 320 °C, S-lens RF level 55%. The LC–HRMS data was analysed using Mass-MetaSite software (Mass Analytica)

Chromatographic separation of M12 metabolites was achieved on a Waters Acquity UPLC BEH C18 column (1.8 µm, 2.1 mm × 100 mm). Mobile phase A was water containing 1% (v/v) formic acid, and mobile phase B was acetonitrile containing 1% (v/v) formic acid. The LC gradient (%B) was 5% (0–2 min), 5–75% (2–12 min), 75–95% (12–14 min), 95% (14–16 min), 95–5% (16–16.5 min), 5% (16.5–18 min). The column temperature was 40 °C and the flow rate was 0.5 ml min^−1^. The injection volume was 10 µl.

### In vitro neddylation and ubiquitination assay

CUL4–RBX1 was neddylated as previously described, in brief, by incubating 12 µM CUL4–RBX1, 1 µM UBE2M, 0.2 µM APPBP1-UBA3, 25 µM NEDD8 at room temperature for 10 min in 25 mM HEPES, 100 mM NaCl, 10 mM MgCl2, 5 mM ATP, pH 7.5. The reaction was quenched by adding 20 mM DTT and was additionally purified by size-exclusion chromatography in 25 mM HEPES, 200 mM NaCl, 1 mM TCEP, pH 7.5. For ubiquitination of GFP–DDX18, 500 nM neddylated CUL4–RBX1 was incubated with 700 nM DCAF11–DDB1–DDA1 and 1 µM GFP–DDX18 with buffer, M12, or GSH-M12 on ice for 20 min. Reaction was performed in 25 mM HEPES, 100 mM NaCl, 10 mM MgCl_2_, 5 mM ATP, pH 7.5 with 2 µM UBE2D, 2 µM UBE2G1, 0.2 µM UBA1, and was initiated by adding 60 µM ubiquitin at room temperature. Samples were taken at indicated timepoints, quenched with SDS sample buffer, and separated by SDS–PAGE. Assay was analysed by detecting fluorescence of GFP–DDX18 on an Amersham Typhoon gel scanner.

### Cryo-EM sample preparation and data processing

DCAF11, DDX18 (residues 171–625), DDB1(ΔBPB) (residue 1–395, 706–1140) and DDA1 were purified in the presence of 10 μM M12 compound and applied to a freshly glow discharged (20 mA for 2 min) Quantifoil UltraAuFoil grid (R0.6/1 and R1.2/1.3). The sample was blotted for 5 s (2 s for R1.2/1.3) after incubation for 10 s at 10 °C with a relative humidity of 90%, and after 3 s (0 s for R1.2/1.3) after blotting plunge frozen into liquid ethane using a Leica EM GP1 plunger (Leica Microsystems). Cryo-EM data were collected on a Titan Krios Electron microscope (Thermo Fisher) at 300 kV equipped with a Falcon 4i detector at the Harvard Cryo-Electron Microscopy Center for Structural Biology using both grids sequentially. Movie stacks were automatically collected using Thermo Scientific Smart EPU software. The dataset of both grids was combined. 10,632 movies were collected with a total dose of 52 e^−^ Å^−2^ over 54 frames, at 0.73 Å per pixel with a nominal magnification of 165,000×, with a defocus range of −0.6 μm to −2.0 μm.

### Electron microscopy data processing and model building

All processing was performed in cryoSPARC (v4.5.3 and 4.6.2)^49^. 10,632 movies were corrected for beam-induced motion, and contrast transfer function was estimated on the fly in cryoSPARC live. 6,549,850 particles were picked with template particle picking, followed by 2D classification. Several rounds of heterogenous refinement led to an initial consensus refinement from 191,769 particles at 2.52 Å, which was used as a seed model to classify 3,767,503 particles from TOPAZ particle picking (v0.2.5a), leading to a consensus refinement of 2.61 Å from 676,855 particles. Heterogeneous refinement and subsequent 3D classification were able to enrich for DDX18 density, leading to a 2.35 Å reconstruction from non-uniform refinement of 131,583 particles following reference-based motion correction, global contrast transfer function refinement, and local contrast transfer function refinement. Local refinement with a soft mask covering DDX18 further improved DDX18 density, yielding a 2.38 Å reconstruction. The two maps were combined into a final composite map using ChimeraX. All unsharpened and sharpened maps were used for model building with Coot (v0.9.8.92 EL)^50^.

Models for DDB1(ΔBPB) (PDB: 5FQD) and AlphaFold predictions for DCAF11 and the C-terminal lobe of DDX18 (residues 402–621) were rigid-body fitted into the density using ChimeraX (v1.8)^51^ and relaxed into the density using ISOLDE (v1.8)^52^. The GSH-M12 compound was built de novo and fit into the density between DCAF11 and DDX18. The model was iteratively refined in PHENIX (realspacerefine v1.21.2-5419)^53,54^ against the composite map and manually inspected in COOT. The non-uniform refinement, DDX18 local refinement, and composite map were deposited in the Electron Microscopy Data Bank under accession codes EMD-71834, EMD-71833 and EMD-71847, respectively. The DDX18–GSH-M12–DCAF11–DDB1(ΔBPB) model was deposited in the Protein Data Bank (PDB) under accession code 9PTU. Structural biology applications used in this project were compiled and configured by SBGrid^55^.

### Chemical synthesis

Additional details are provided in the Supplementary Information.

### Reporting summary

Further information on research design is available in the Nature Portfolio Reporting Summary linked to this article.

## Online content

Any methods, additional references, Nature Portfolio reporting summaries, source data, extended data, supplementary information, acknowledgements, peer review information; details of author contributions and competing interests; and statements of data and code availability are available at https://doi.org/10.1038/s41586-026-10873-1.

## Supplementary information

Supplementary Information This file contains Supplementary Fig. 1: uncropped gel images; Supplementary Fig. 2: gating strategy for flow cytometry; and Supplementary Methods: chemical synthesis.

Reporting Summary

Supplementary Data Source data for main figures and extended data figures.

Peer Review File

## Figures and Tables

**Extended Data Table 1:**
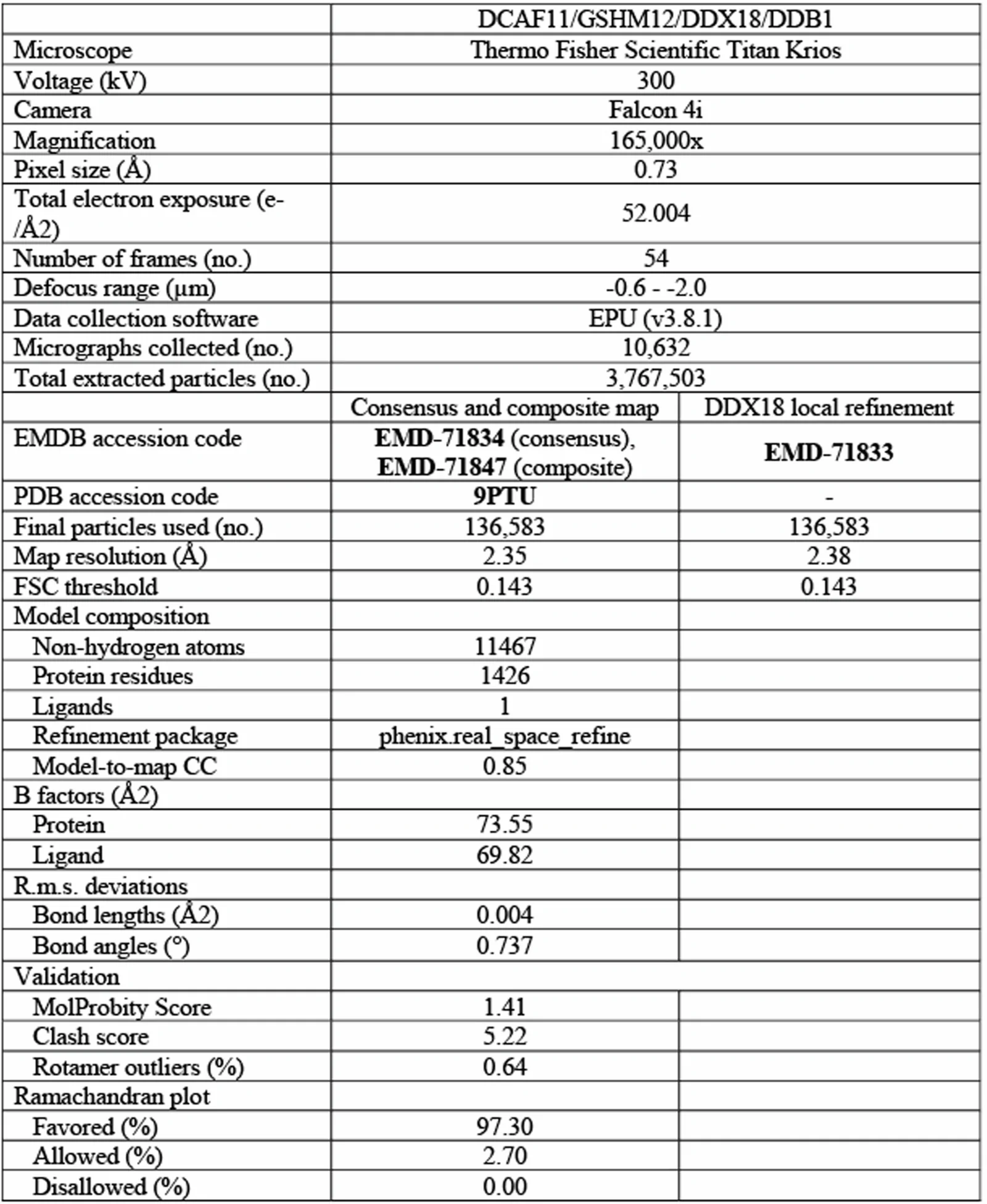
Data collection and refinement statistics

## Data Availability

The raw proteomics datasets generated during this study are available at PRIDE accessions: PXD067016, PXD067063, PXD067066, PXD067067, PXD067070 and PXD067076. Cryo-EM data are available from the Electron Microscopy Data Bank under accessions EMD-71847, EMD-71833, EMD-71834 and the PDB under accession 9PTU.

## Code availability

The code necessary to reproduce the statistical analysis in this study can be found at https://github.com/Yoonlab-MDA/DCAF11_Nature2026.
