# Supplementary material for: DCAF11-dependent molecular glue degrader activated by glutathionylation
Source: Nature. 2026 Aug 5;657(8133):1045–52. doi: 10.1038/s41586-026-10873-1 (PMC13600927; doi:10.1038/s41586-026-10873-1)
Supplement: Supplementary file 1 [file 41586_2026_10873_MOESM1_ESM.pdf]

nature portfolio
https://doi.org/10.1038/s41586-026-10873-1

Supplementary information

DCAF11-dependent molecular glue degrader activated by glutathionylation

In the format provided by the authors and unedited

Supplementary Figure 1: Uncropped gel images

Fig 1e

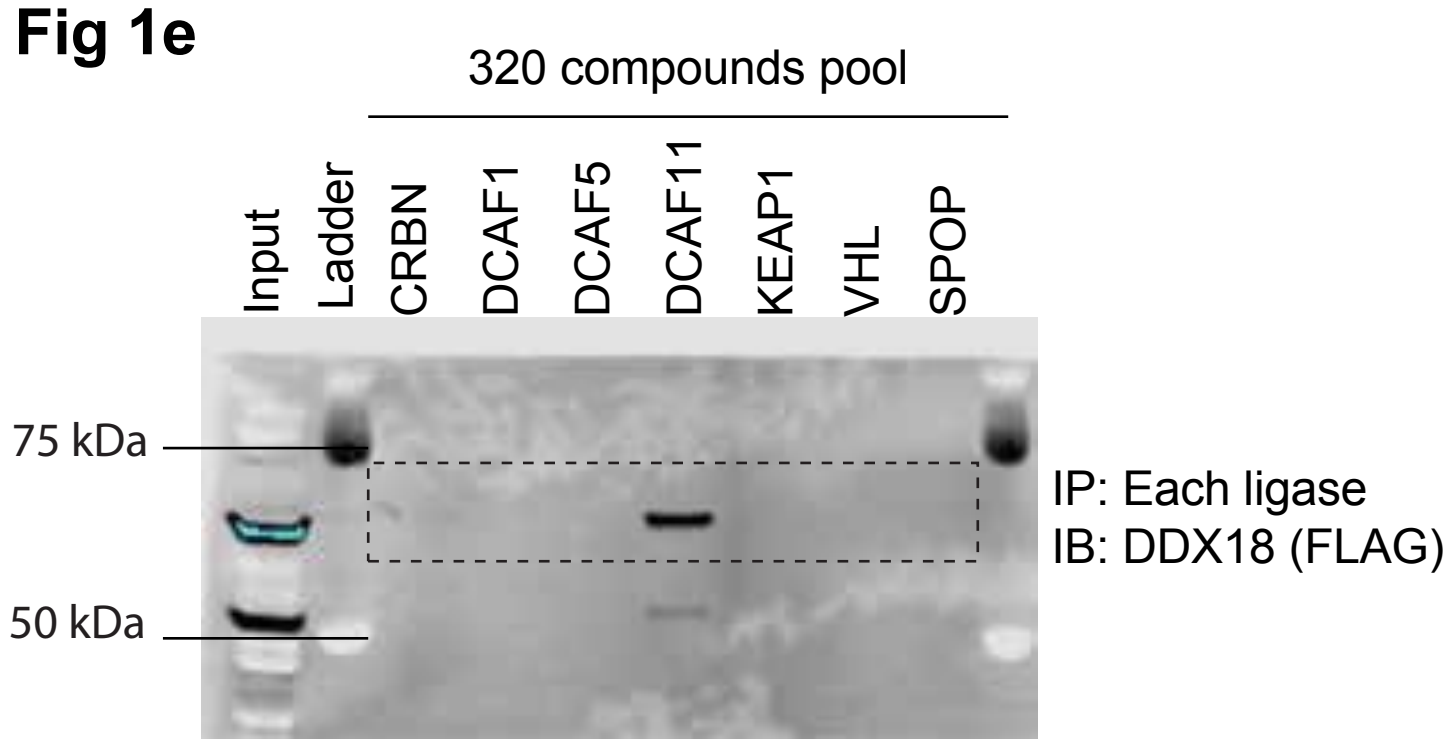

Fig 1g

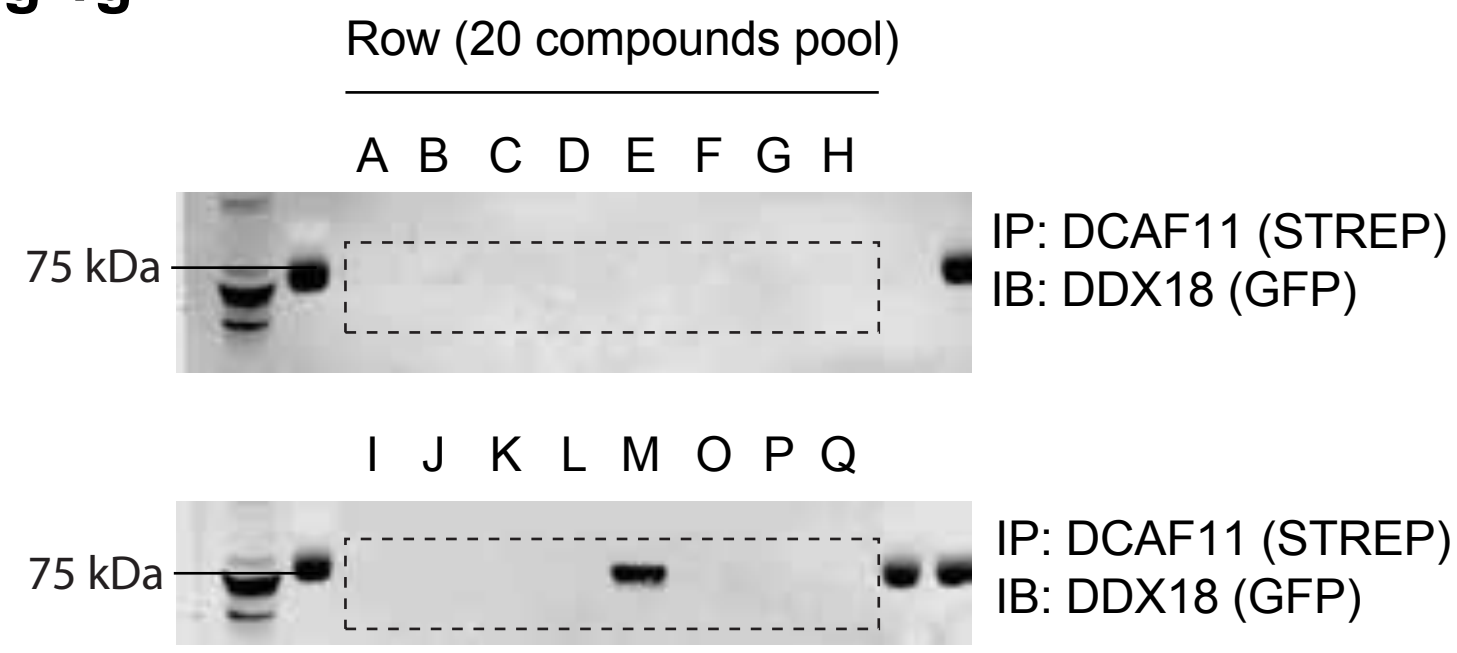

Fig 1i

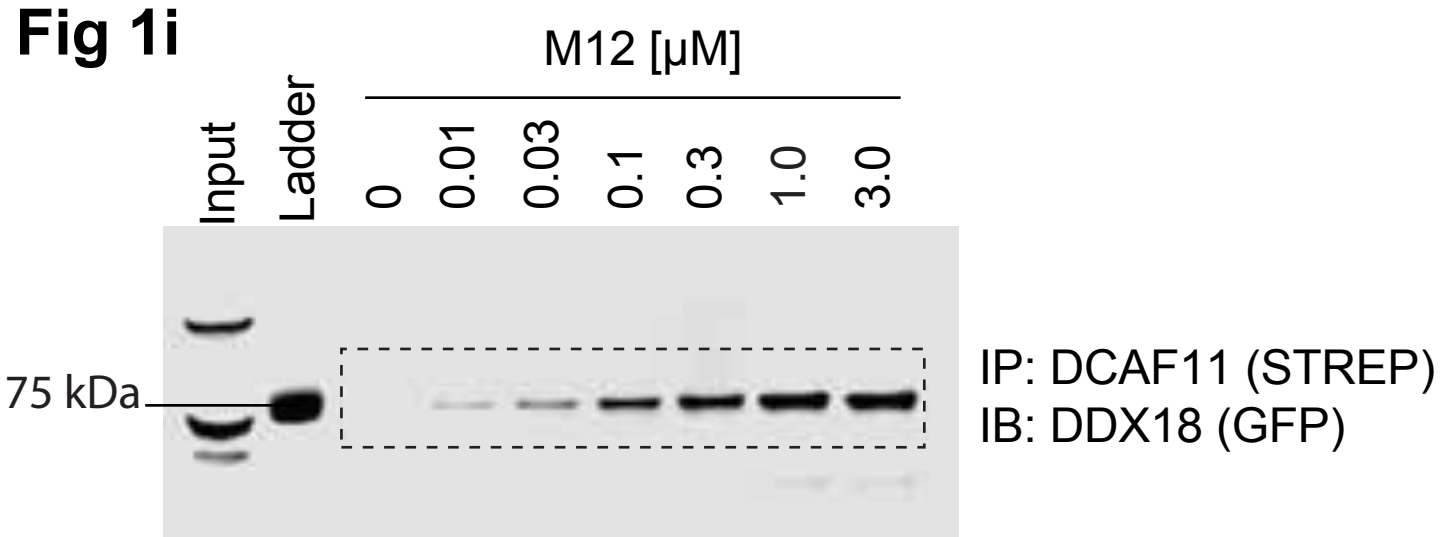

Fig 2b

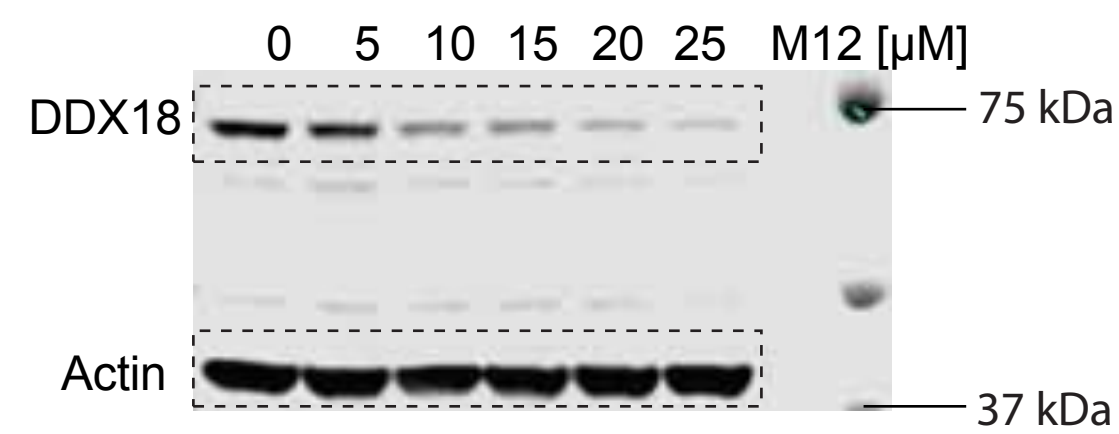

Fig 2b

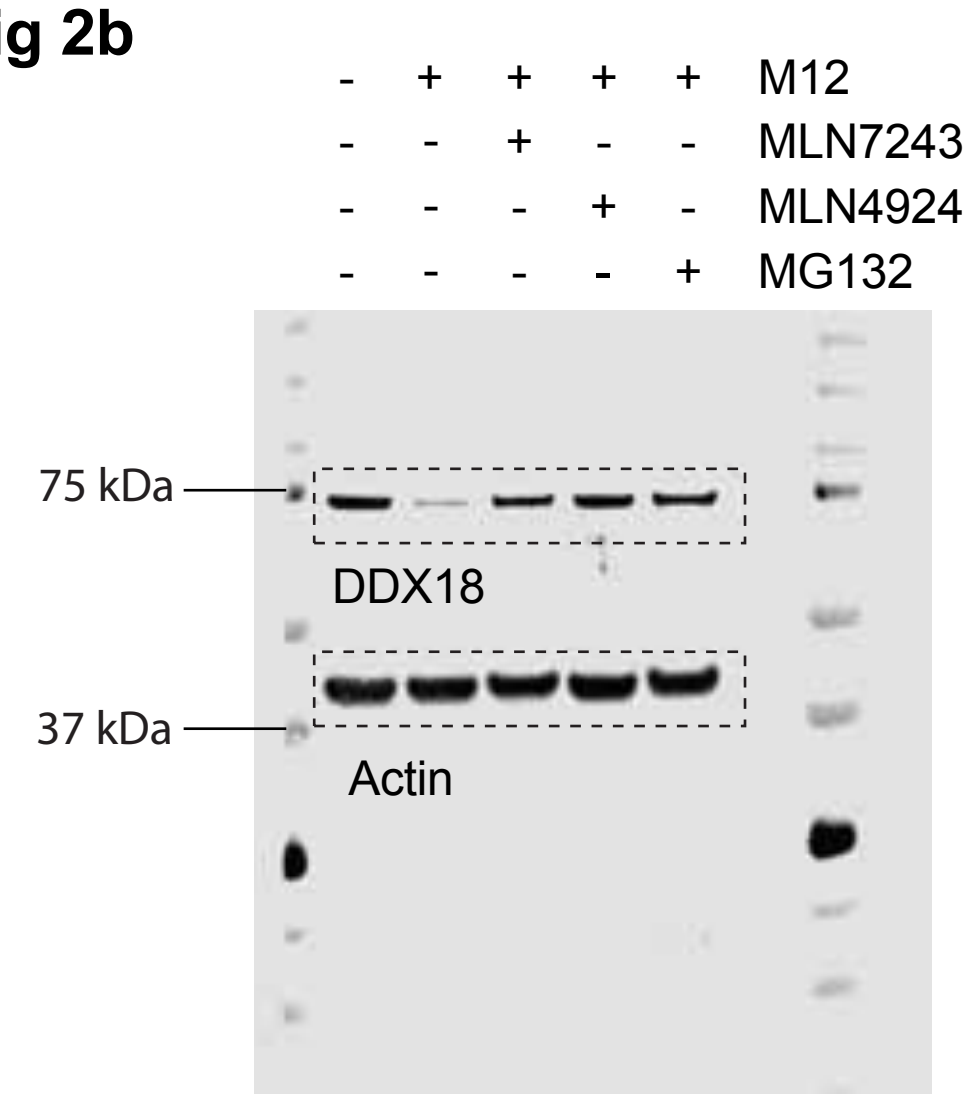

Fig 2g

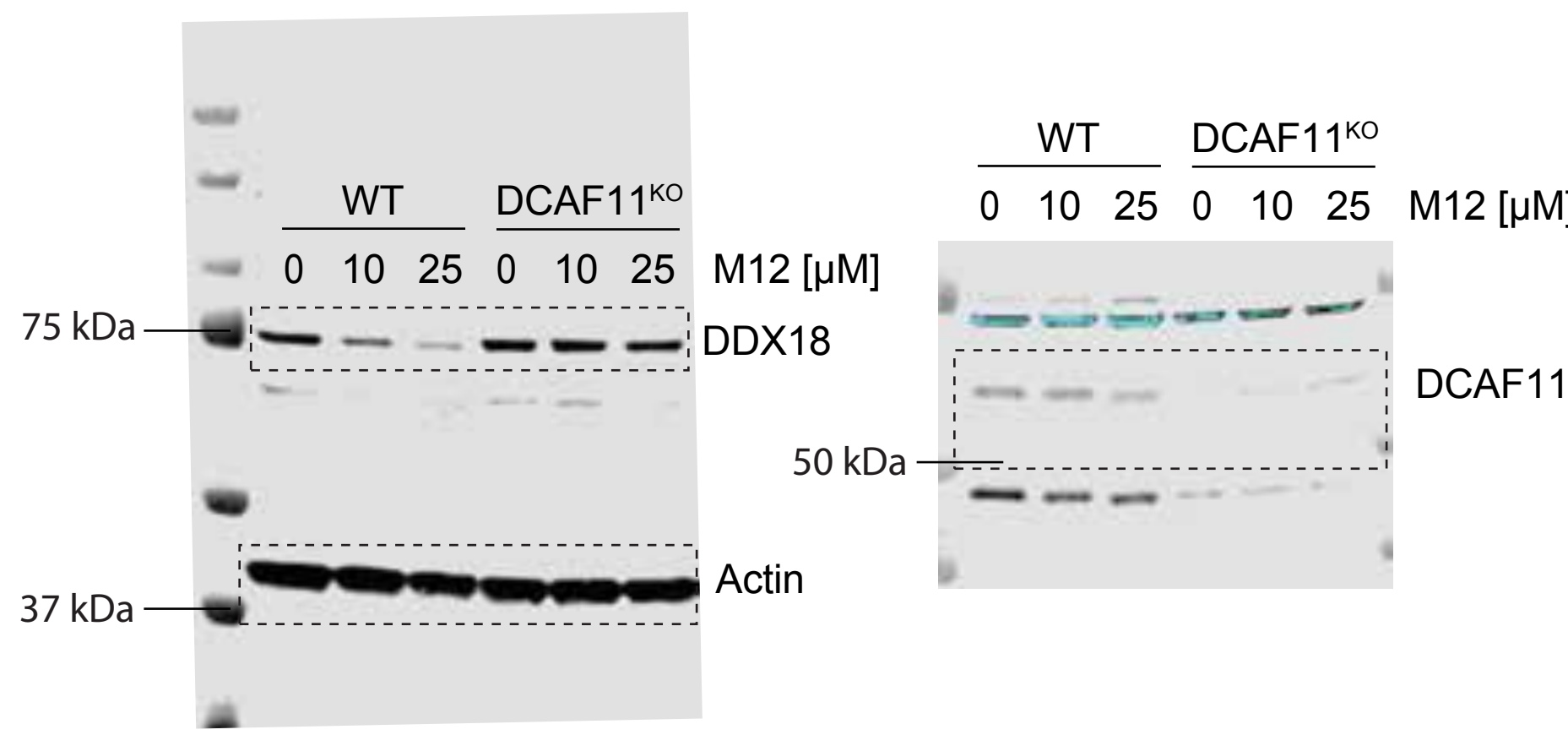

Fig 3c

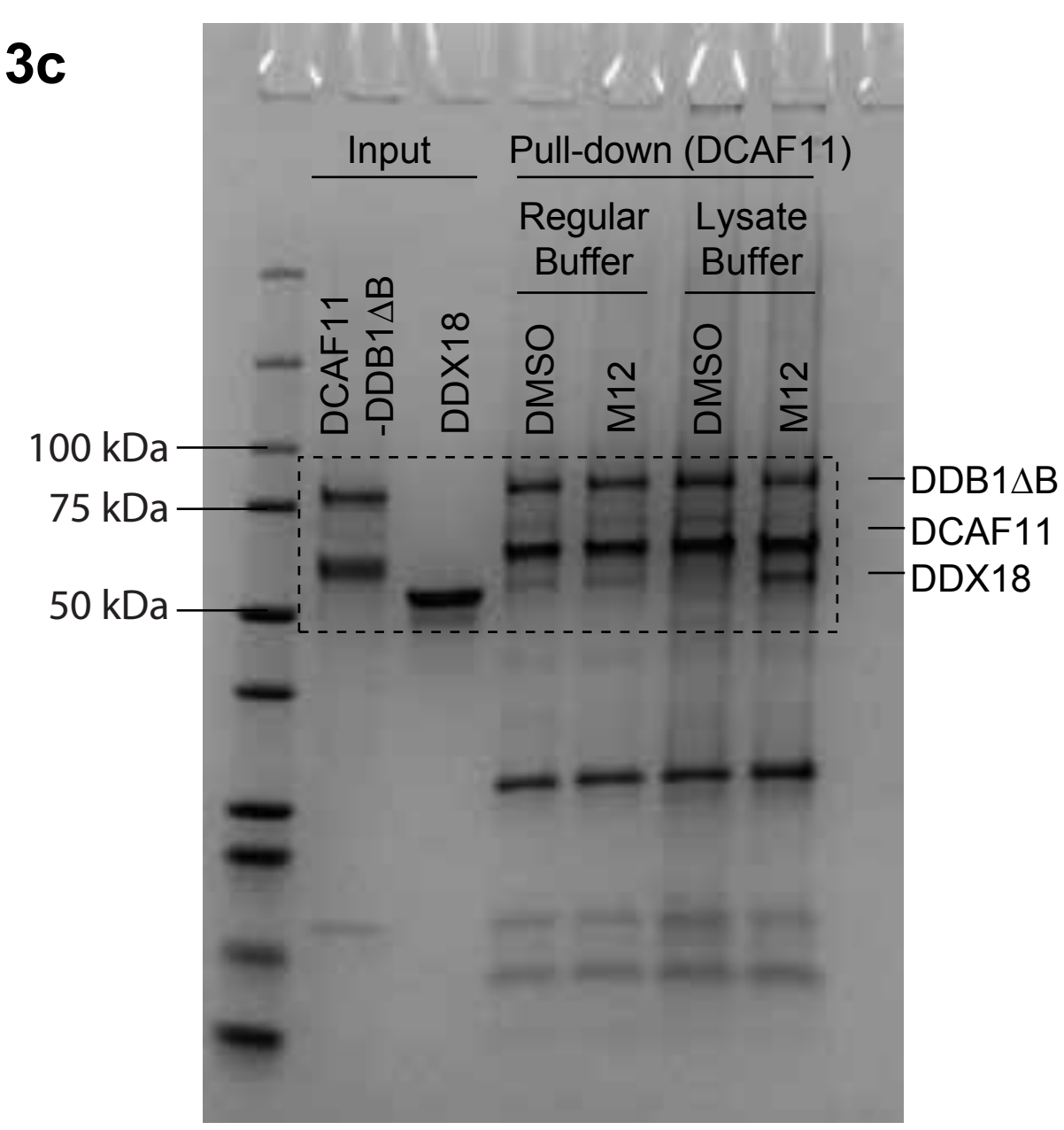

Fig 4e

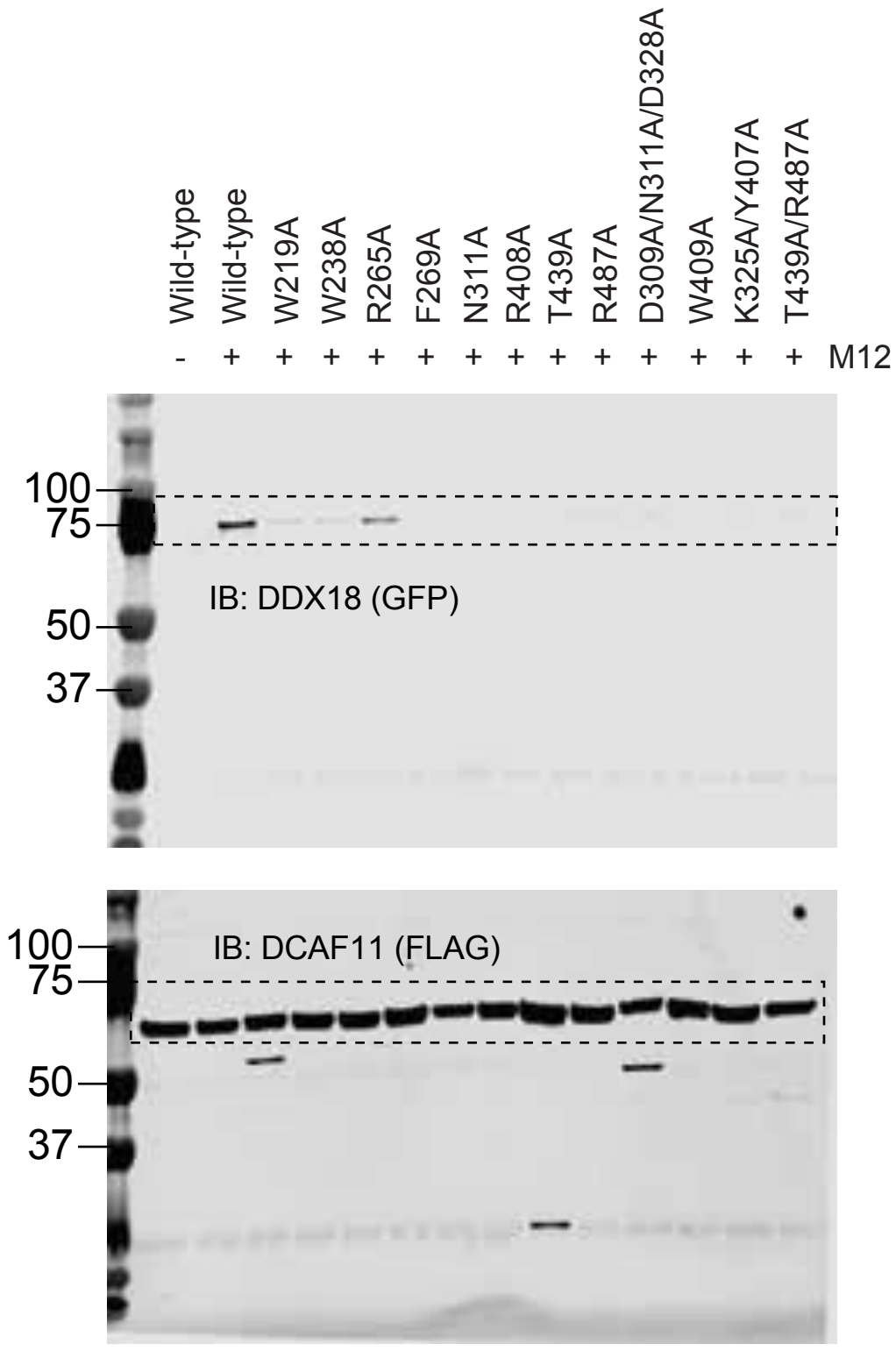

Fig 5d

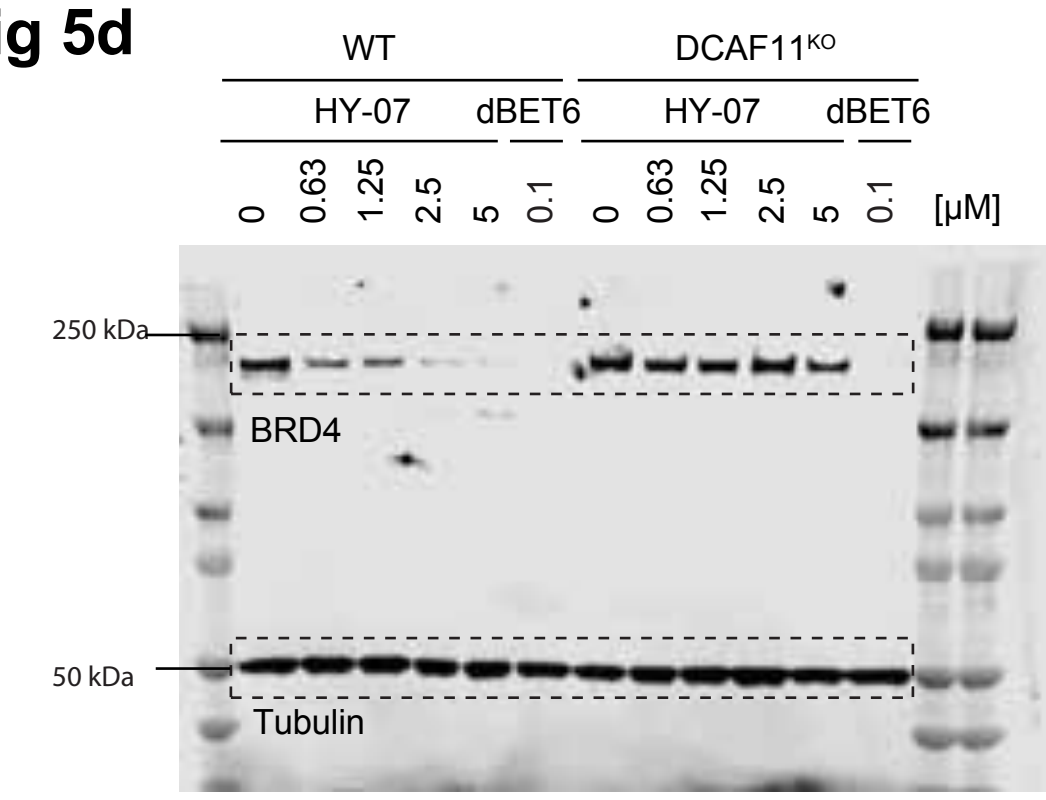

Fig 5f

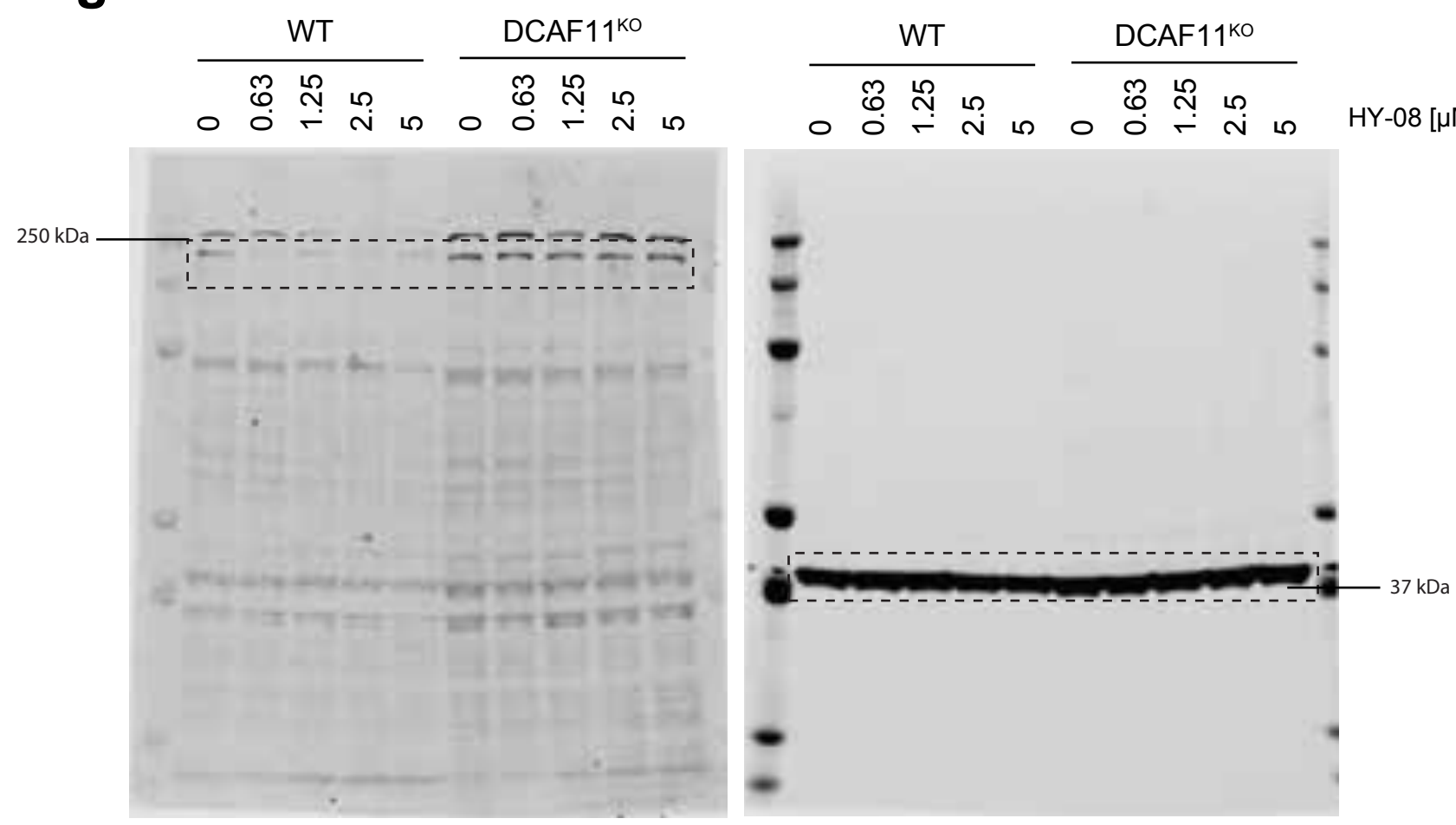

Fig 5i

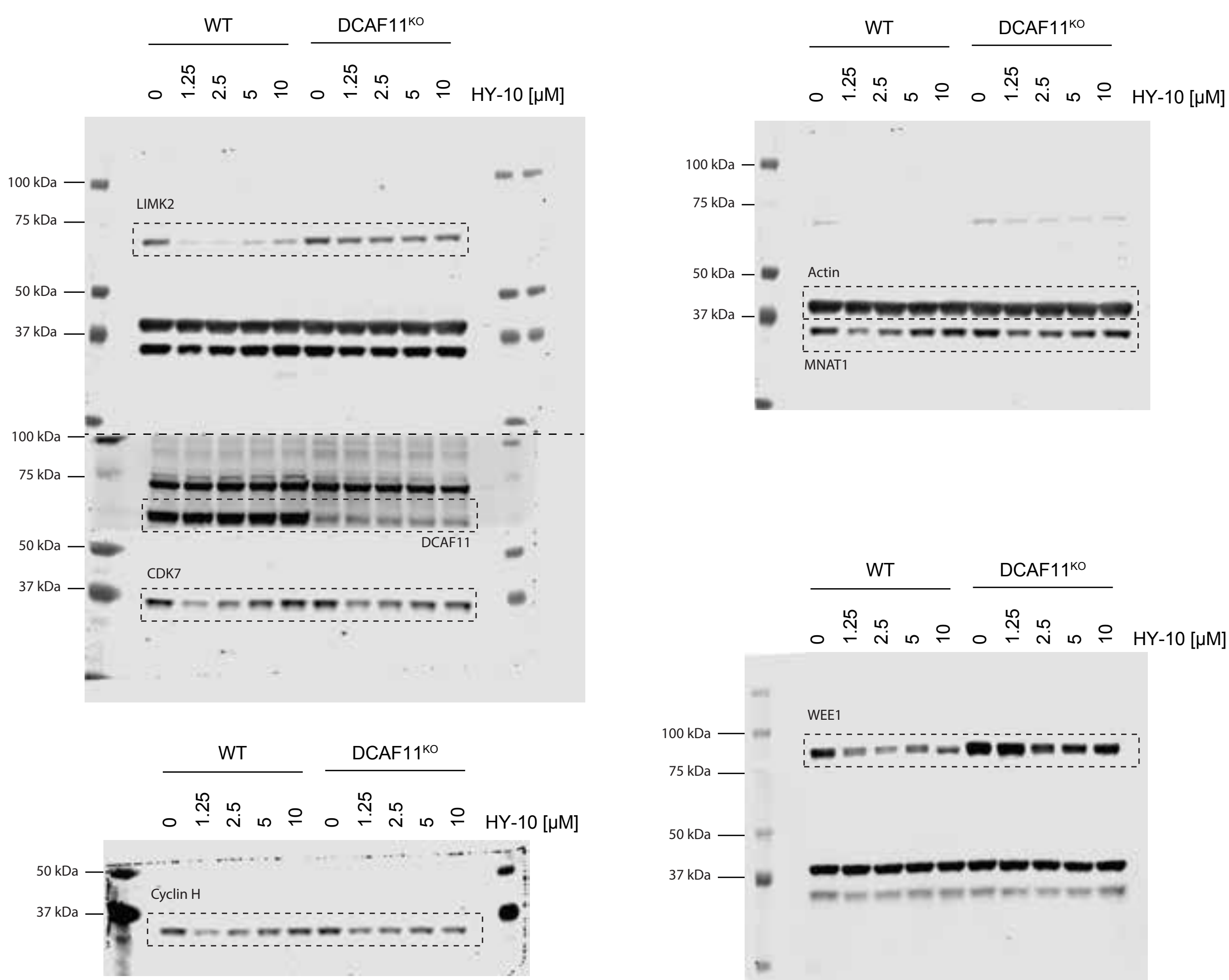

Extended Data Fig 1b

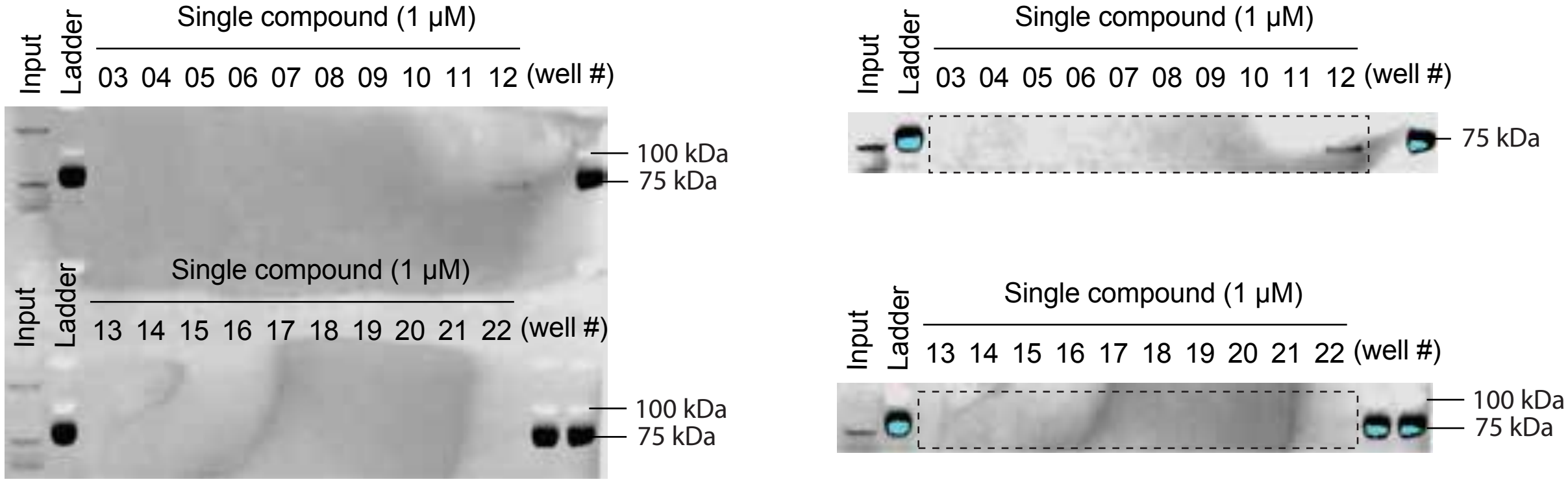

Extended Data Fig 1c

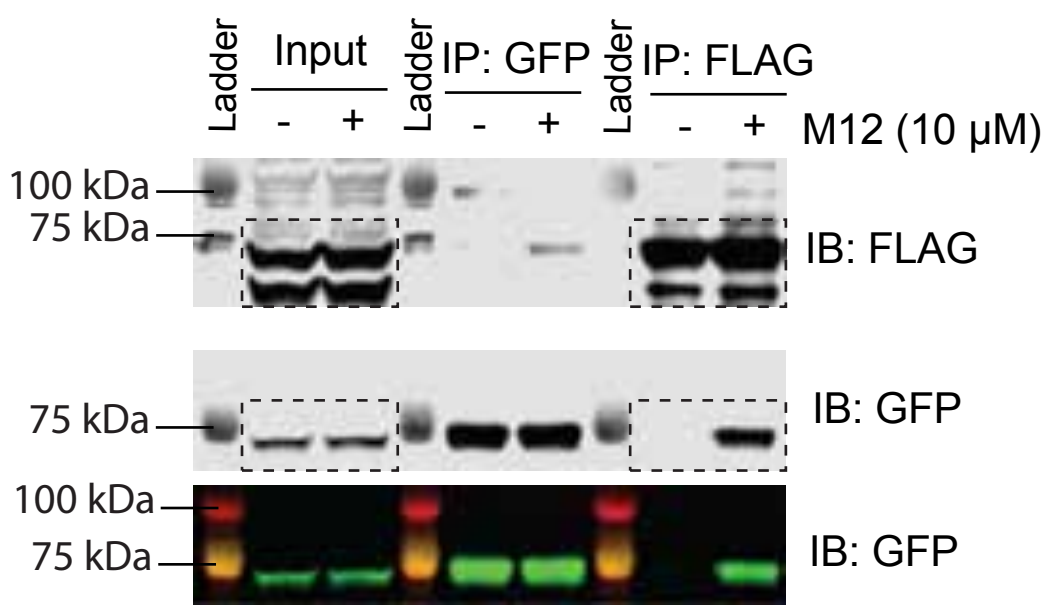

Extended Data Fig 2g

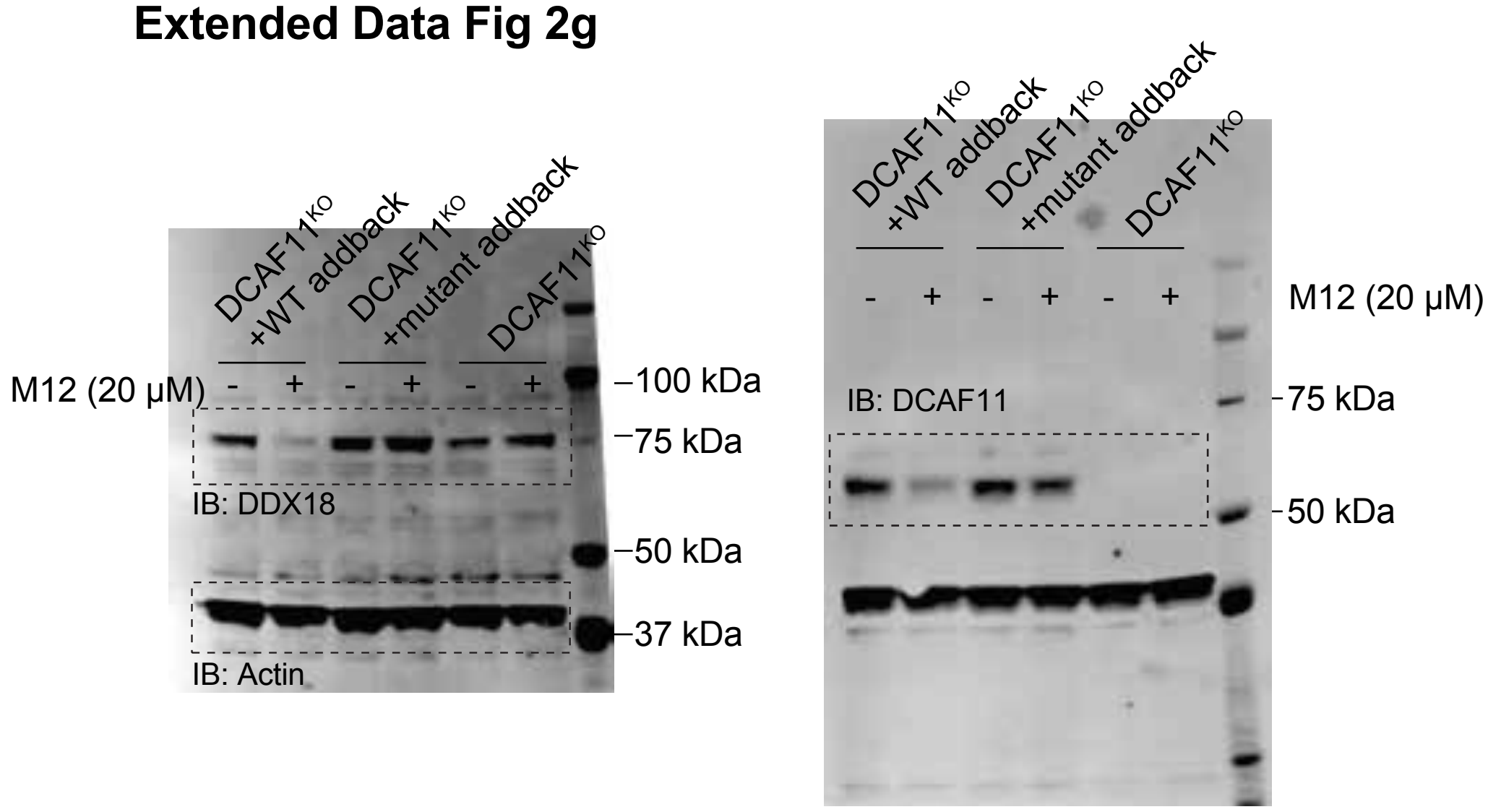

Extended Data Fig 3i

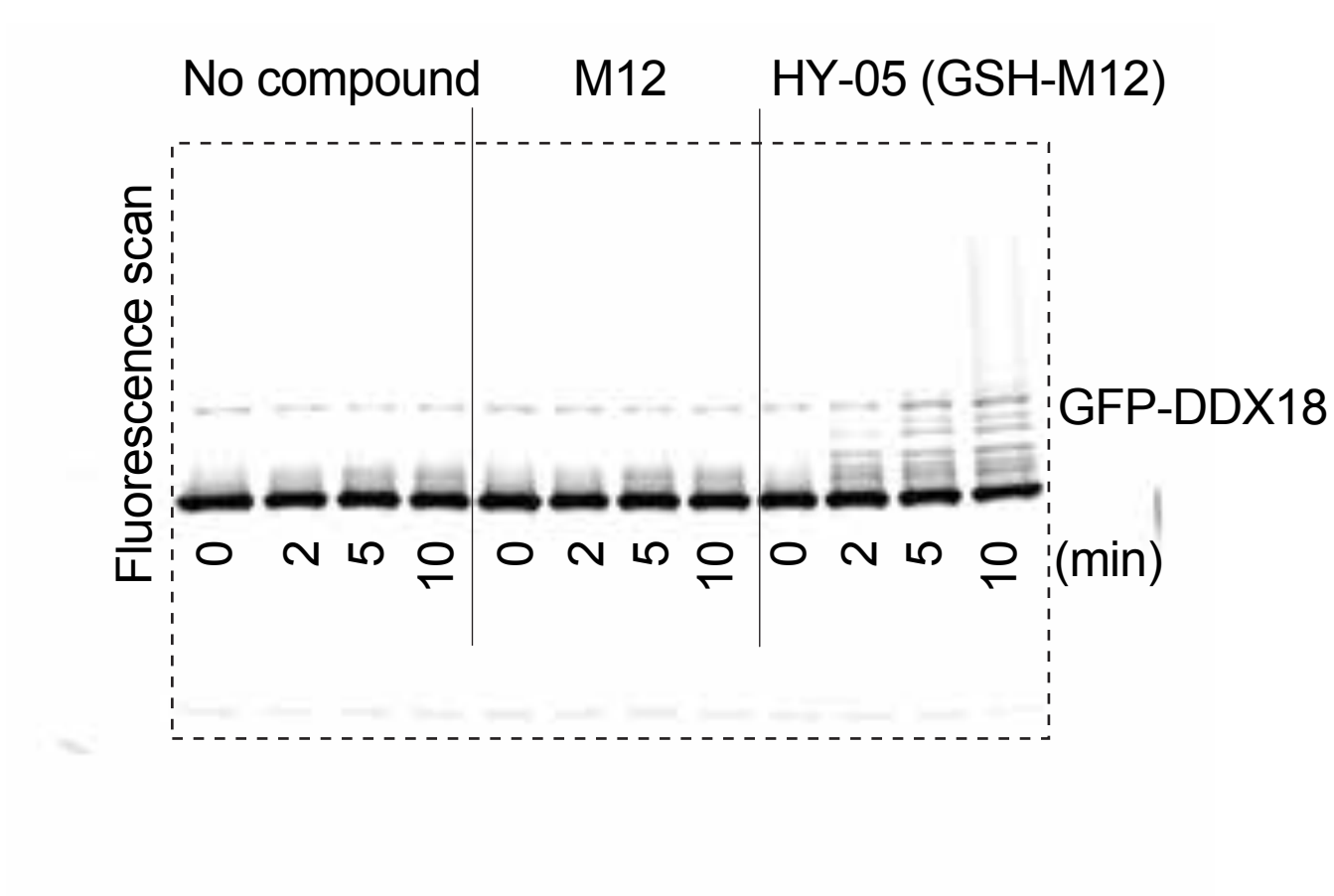

Extended Data Fig 5e

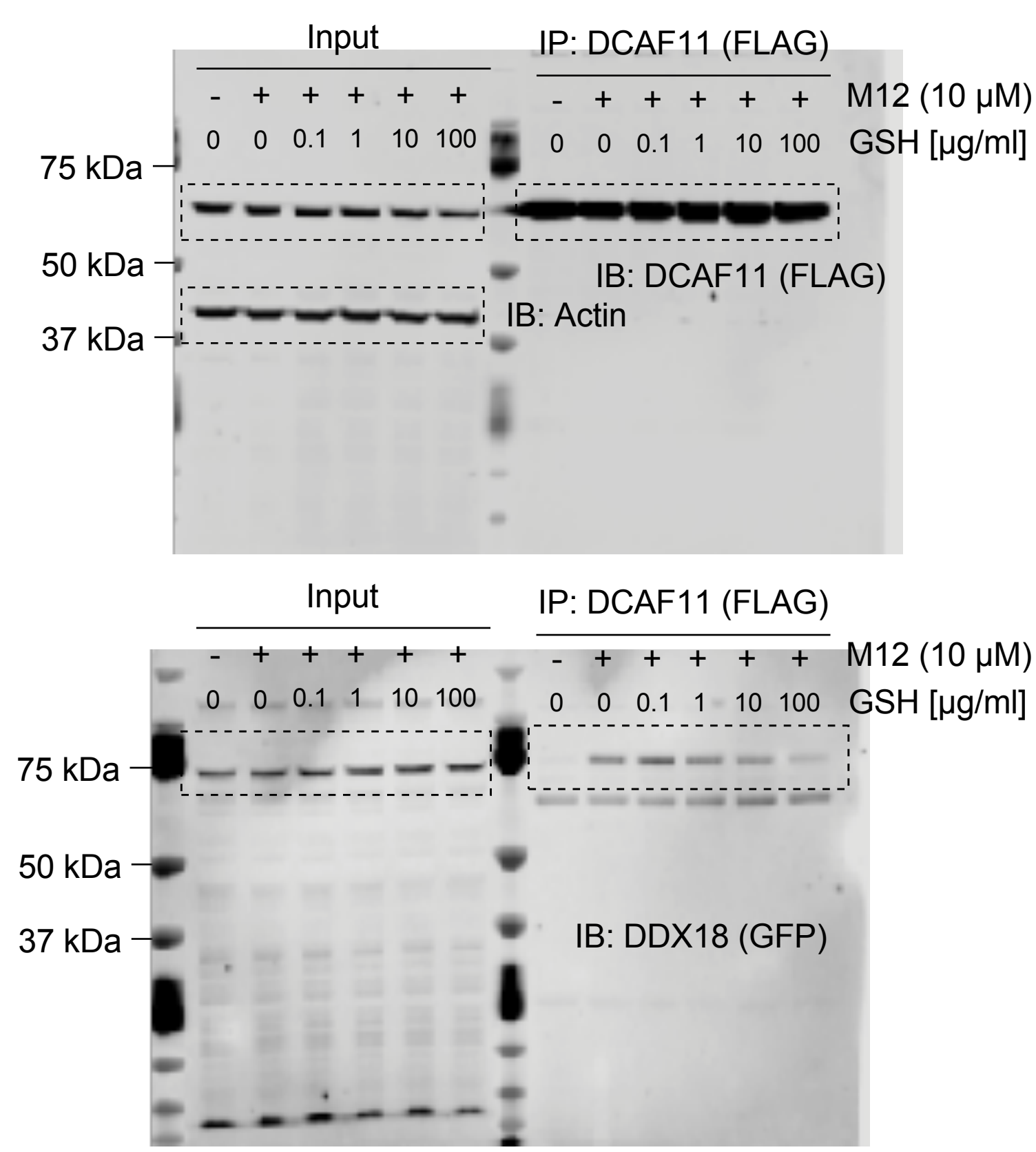

Extended Data Fig 6g

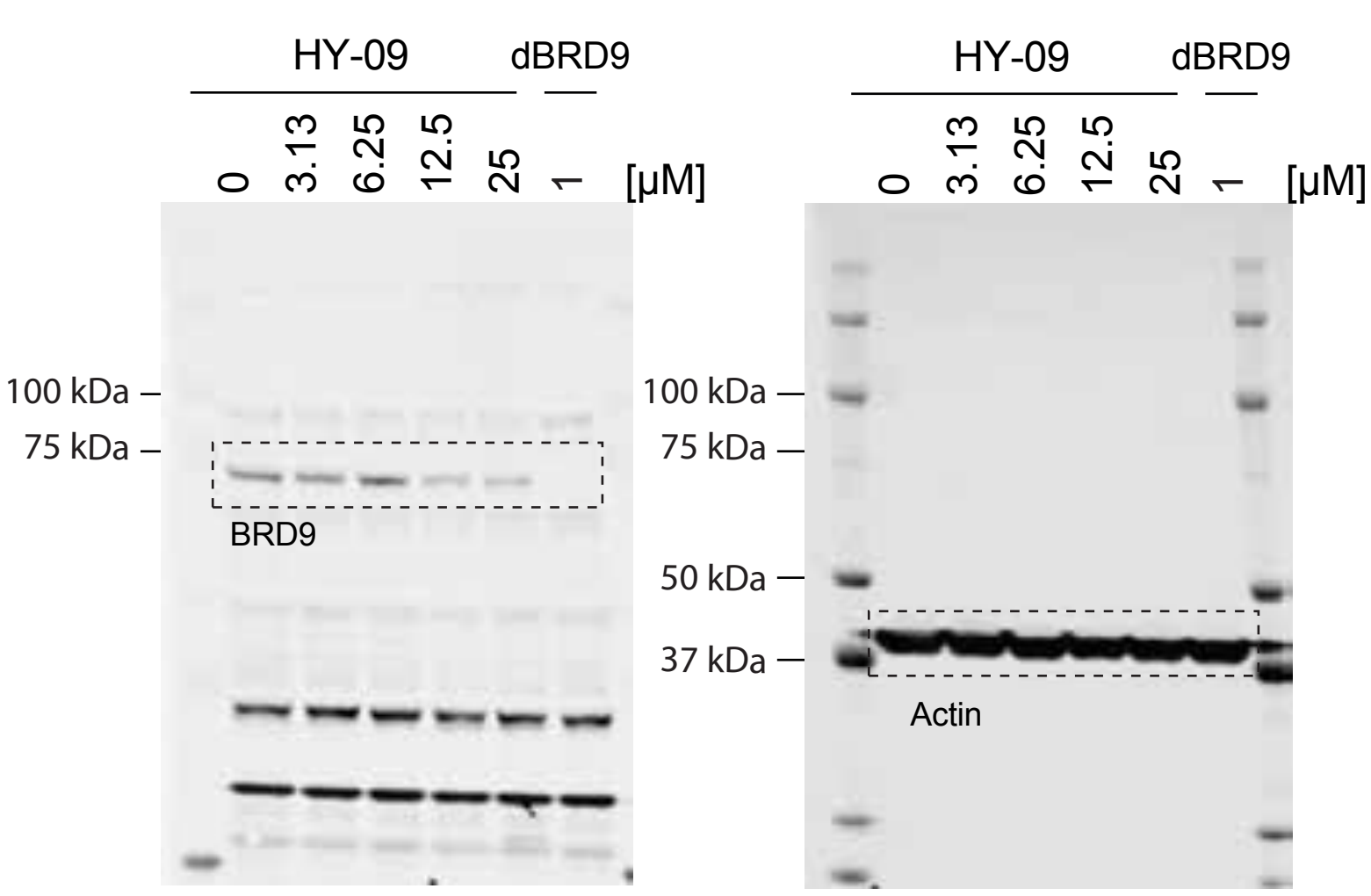

Extended Data Fig 3i. Replicate with different time points

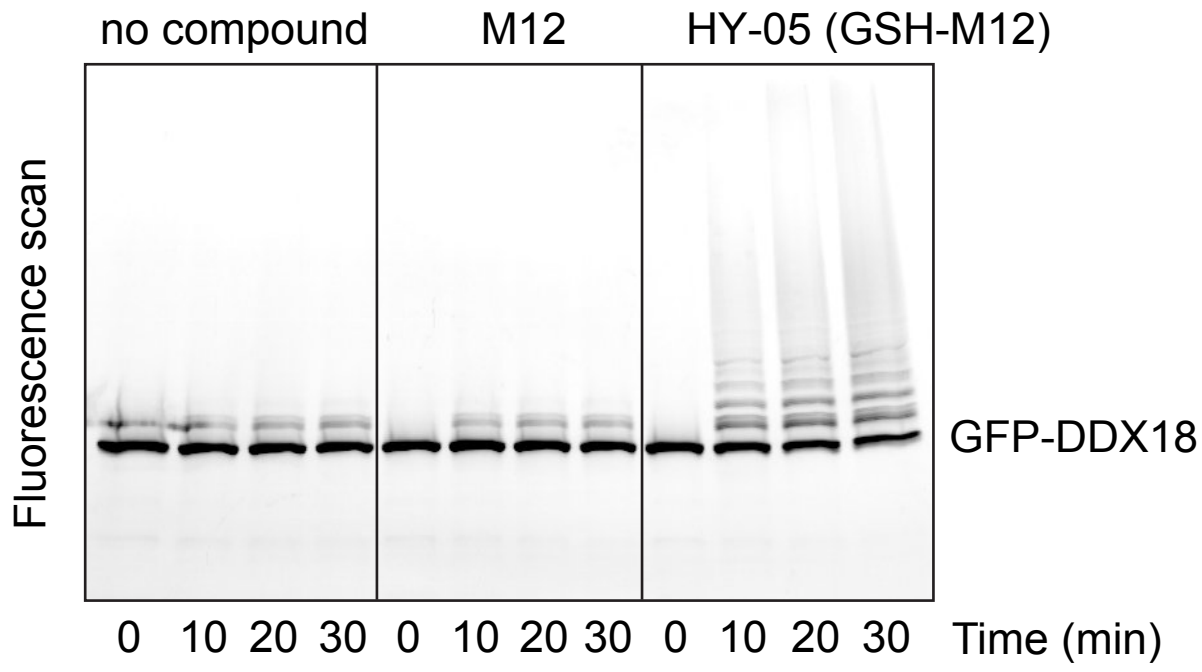

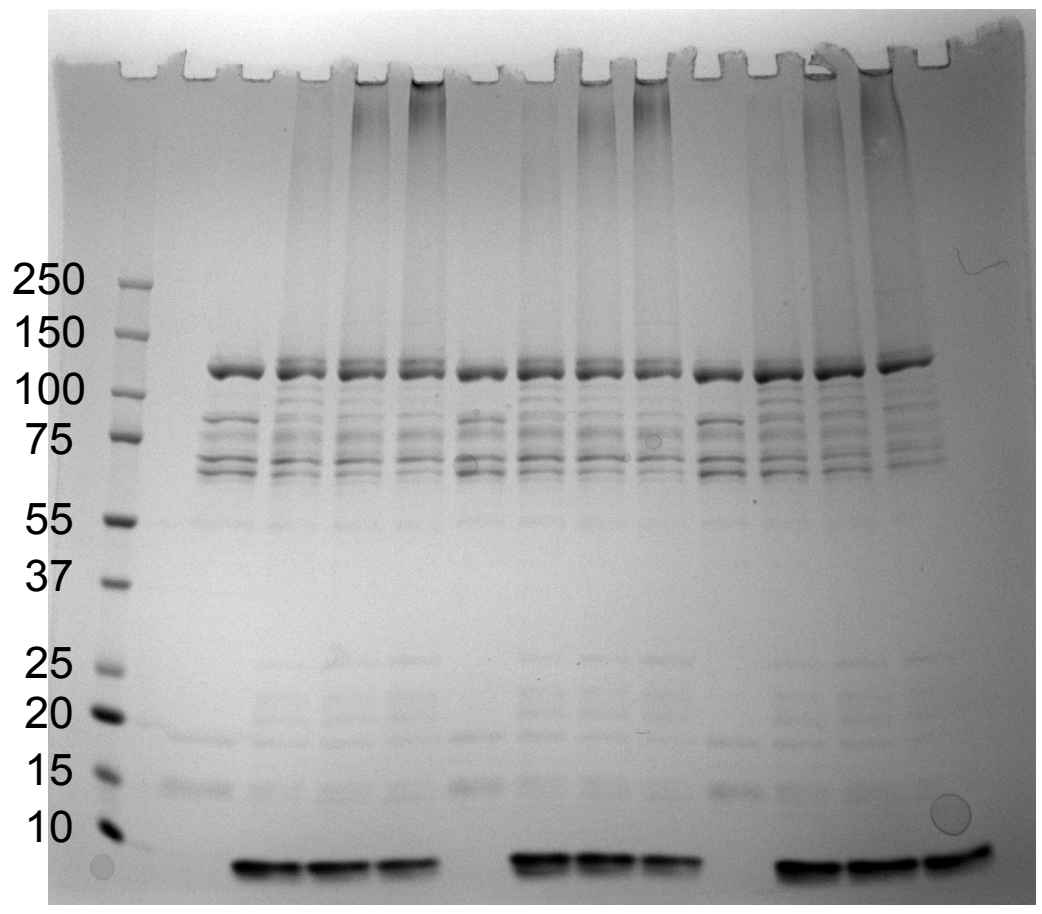

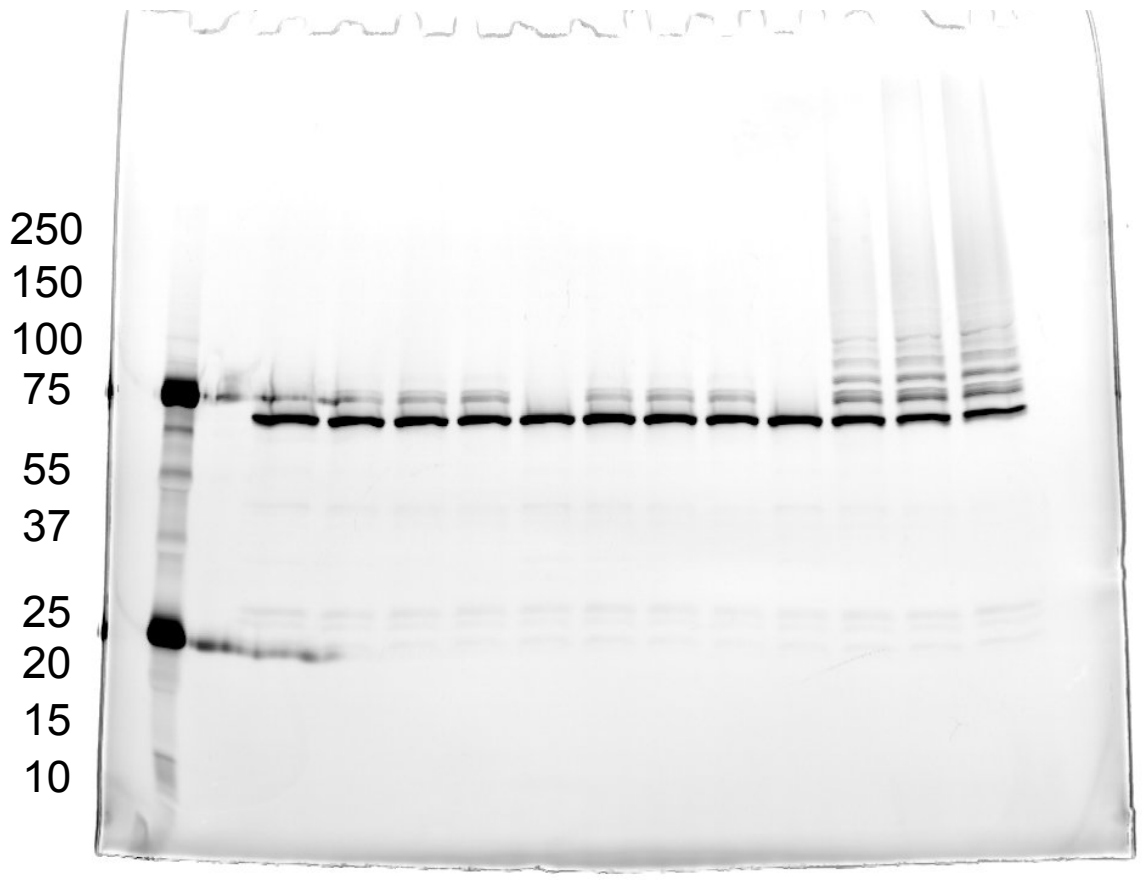

Supplementary Figure 2: Gating strategy for flow cytometry

a Gating strategy for all flow cytometry experiments - described in Slabicki *et al. Nature* 2020. PMID: 33208943

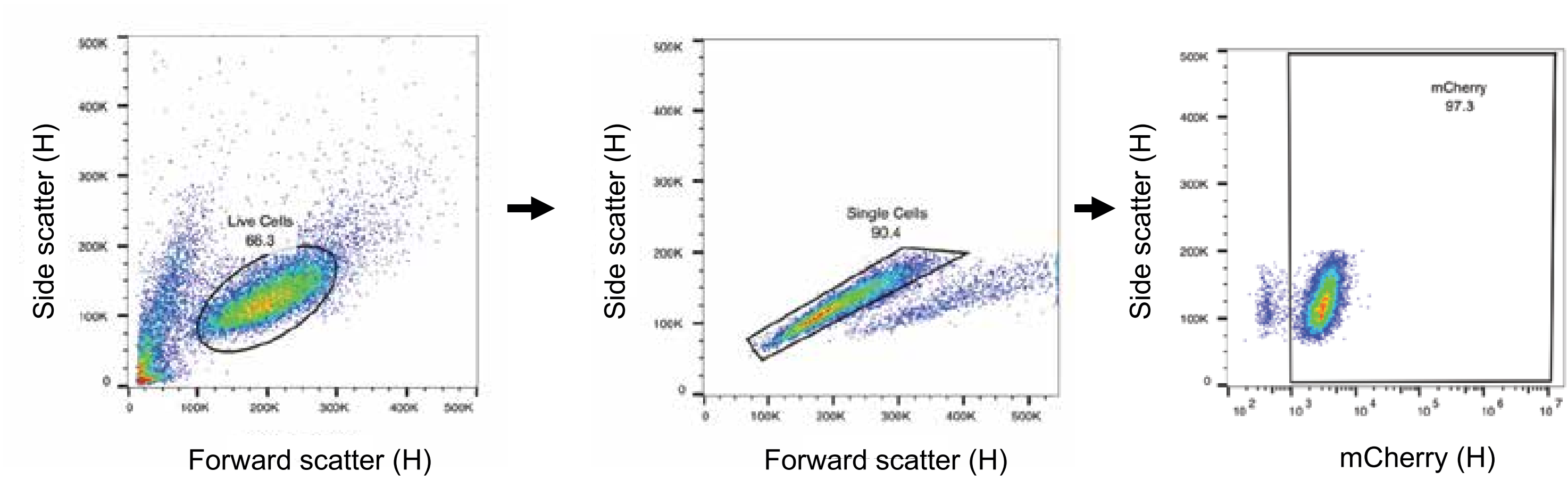

b Gating strategy for FACS sorting screens

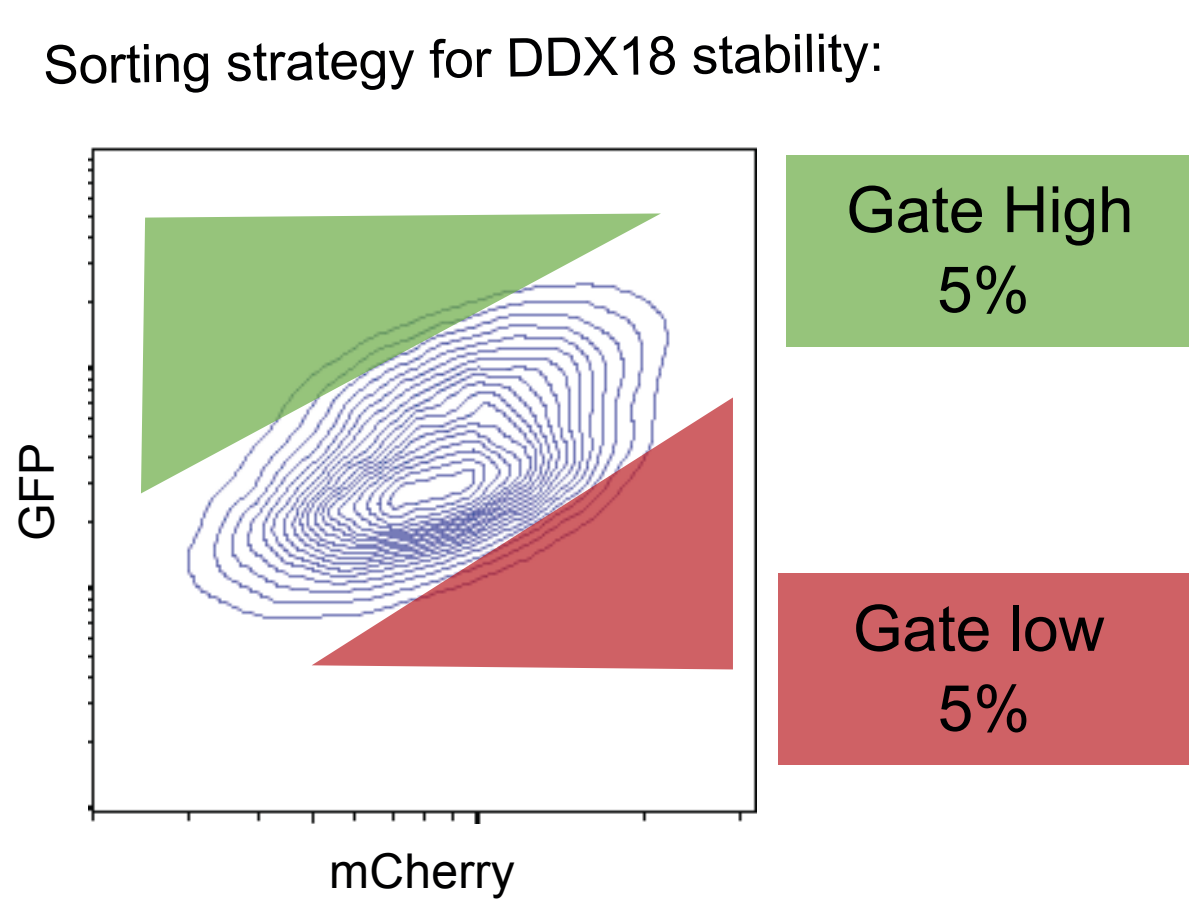

Supplementary Note: Synthesis of Compounds and characterization

General Information. All reactions were carried out under an inert atmosphere of dry nitrogen or argon in oven or flame-dried glassware, unless the reaction procedure states otherwise. Tetrahydrofuran (THF), 1,4-dioxane, toluene and other solvents were purchased from commercial sources. Reaction temperatures were controlled by TY98-1 (Shang Hai SiLe). Analytical thin-layer chromatography (TLC) was performed using pre-coated TLC plates and visualized using combinations of UV, anisaldehyde, ceric ammonium molybdate (CAM), potassium permanganate, and iodine staining. Flash column chromatography was preformed using 100-200 mesh silica gel (LCGY-FD0100) as the stationary phase. Proton nuclear magnetic resonance spectra were recorded at Agilent–400 MHz spectrometer. Carbon nuclear magnetic resonance spectra were recorded at Agilent–100 MHz spectrometer. Optical Rotations were measured on a Rudolph Autopol III S2 polarimeter. LCMS were measured on an Agilent 1260+6120. HPLC were measured on a Shimadzu LC-20. SFC were measured on a Waters UPCC.

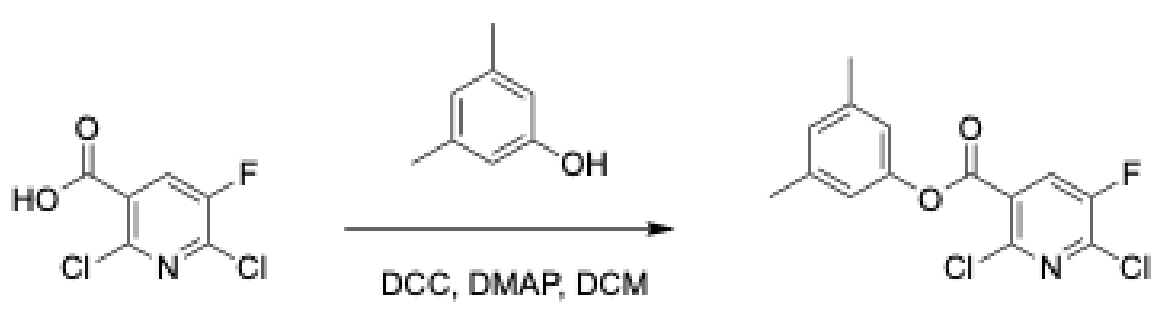

(3,5-dimethylphenyl) 2,6-dichloro-5-fluoro-pyridine-3-carboxylate (M12)

To the mixture of 2,6-dichloro-5-fluoro-pyridine-3-carboxylic acid (5 g, 23.81 mmol, 1 eq) and 3,5-dimethylphenol (2.33 g, 19.05 mmol, 0.8 eq) in DCM (100 mL) was added DCC (7.37 g, 35.72 mmol, 7.22 mL, 1.5 eq) and DMAP (290.89 mg, 2.38 mmol, 0.1 eq). The mixture was stirred at 20 °C for 2 h. The residue was filtered and the filtrate was concentrated in vacuum. The residue was purified by flash silica gel chromatography (ISCO®; 40 g SepaFlash® Silica Flash Column, Eluent of 0~15% Ethyl acetate/Petroleum ether gradient @ 80 mL/min) to the title compound (1.58 g, 5.03 mmol, 100% purity) as a white solid. MS (m/z) ($M+H^+$): found 314.0, calculated 313.0. ^1H NMR (400 MHz, DMSO-d_6) δ (ppm): 8.70 (d, J = 7.6 Hz, 1H), 6.98 (s, 1H), 6.94 (s, 2H), 2.31 (s, 6H)

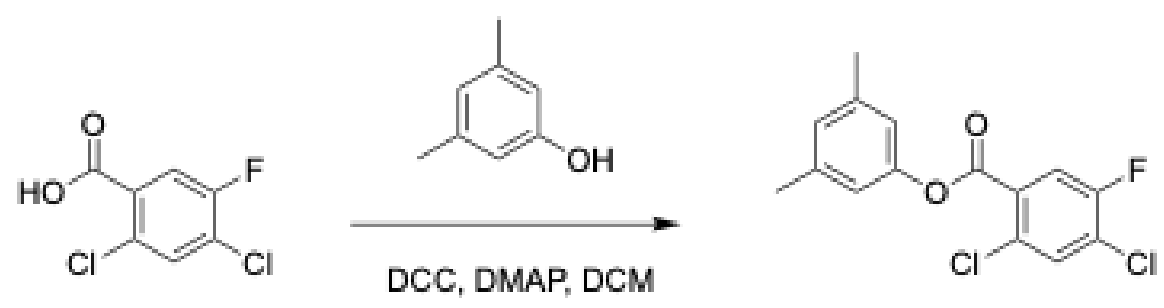

HY-01

(3,5-dimethylphenyl) 2,4-dichloro-5-fluoro-benzoate (HY-01)

HY-01 was synthesized similarly as M12 with 2,4-dichloro-5-fluoro-benzoic acid. MS (m/z) ($M+H^+$): found 313.0, calculated 312.0. ^{1}H NMR (400 MHz, DMSO-d_6) δ (ppm): 8.16 (d, J = 9.2 Hz, 1H), 8.08 (d, J = 6.4 Hz, 1H), 6.98 (s, 1H), 6.93 (s, 2H), 2.31 (s, 6H).

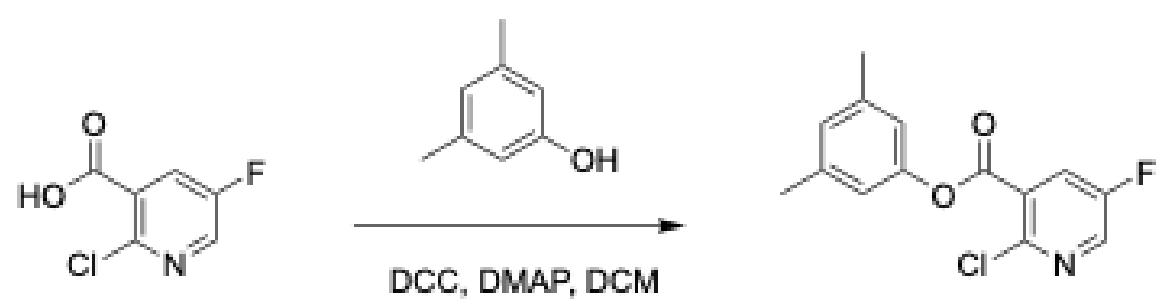

HY-02

(3,5-dimethylphenyl) 2-chloro-5-fluoro-pyridine-3-carboxylate (HY-02)

HY-02 was synthesized similarly as M12 with 2-chloro-5-fluoro-pyridine-3-carboxylic. MS (m/z) ($M+H^+$): found 280.0, calculated 279.0. ^{1}H NMR (400 MHz, DMSO-d_6) δ (ppm): 8.76 (d, J = 2.8 Hz, 1H), 8.52 (dd, J = 8.0 Hz, 2.8 Hz, 1H), 6.99 (s, 1H), 6.95 (s, 2H), 2.32 (s, 6H).

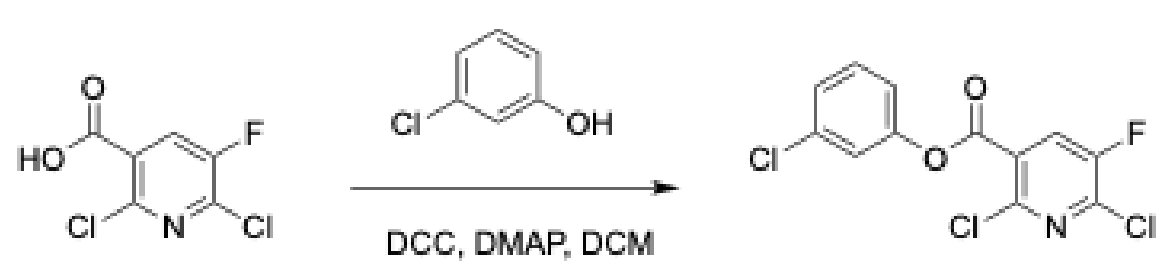

HY-03

(3-chlorophenyl) 2,6-dichloro-5-fluoro-pyridine-3-carboxylate (HY-03)

HY-03 was synthesized similarly as M12 with 3-chlorophenol. ^{1}H NMR (400 MHz, DMSO-d_6) δ (ppm): 8.77 (d, J = 8.0 Hz, 1H), 7.58 - 7.52 (m, 2H), 7.48 - 7.42 (m, 1H), 7.36 (m, 1H).

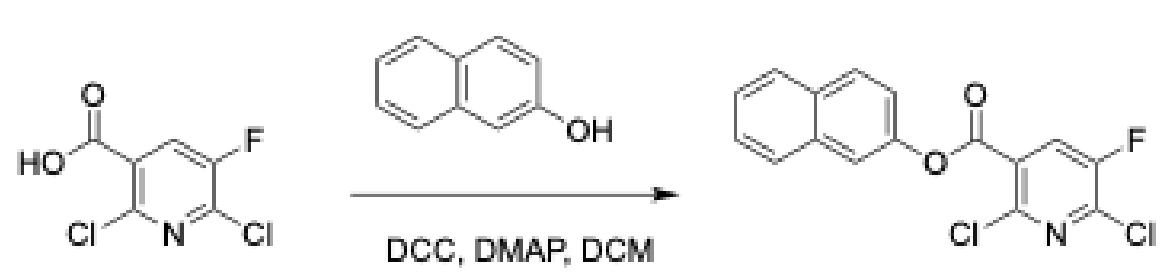

HY-04

2-naphthyl 2,6-dichloro-5-fluoro-pyridine-3-carboxylate (HY-04)

HY-04 was synthesized similarly as M12 with naphthalen-2-ol. MS (m/z) ($M+H^+$): found 336.0, calculated 336.0. ^{1}H NMR (400 MHz, DMSO-d_6) δ (ppm): 8.83 (d, J = 8.0 Hz, 1H), 8.07 (d, J = 8.8 Hz, 1H), 8.03 - 7.95 (m, 2H), 7.91 (d, J = 2.0 Hz, 1H), 7.62 - 7.55 (m, 2H), 7.53 (m, 1H).

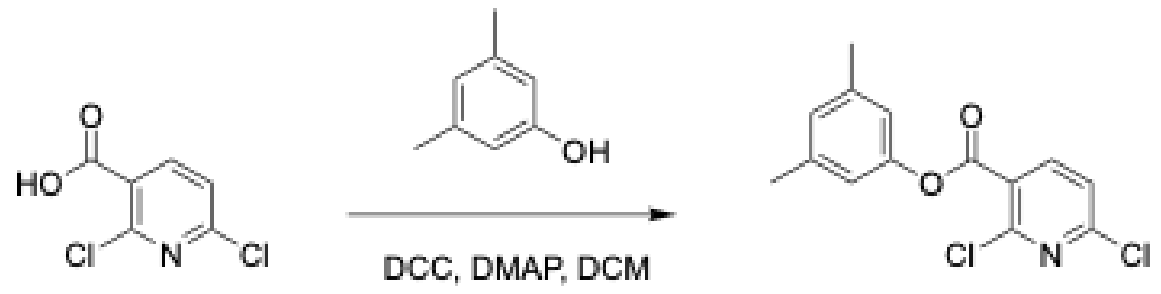

HY-60

(3, 5-dimethylphenyl) 2, 6-dichloropyridine-3-carboxylate (HY-60)

HY-60 was synthesized similarly as M12. MS (m/z) ($M+H^+$): found 296.0, calculated 295.0. ^1H NMR (400 MHz, DMSO-d_6) δ (ppm): 8.55 (d, J = 8.4 Hz, 1H), 7.81 (d, J = 8.0 Hz, 1H), 6.98 (s, 1H), 6.94 (s, 2H), 2.31 (s, 6H).

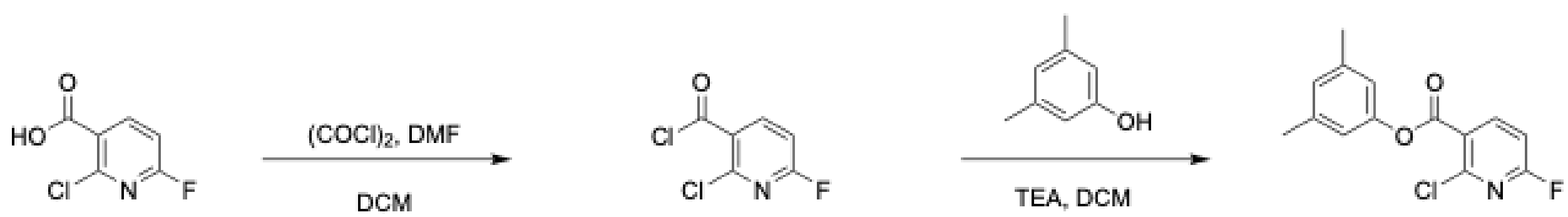

HY-1A

(3, 5-dimethylphenyl) 2-chloro-6-fluoro-pyridine-3-carboxylate (HY-1A)

- <u>Step 1: 2-chloro-6-fluoro-pyridine-3-carbonyl chloride</u>

To a solution of 2-chloro-6-fluoro-pyridine-3-carboxylic acid (300 mg, 1.71 mmol, 1.0 *eq*) in DCM (3 mL) was added $(COCl)_2$ (522 mg, 4.11 mmol, 360 µL, 2.4 *eq*) and DMF (12.5 mg, 171 µmol, 13.2 µL, 0.1 *eq*) at 0 °C. The mixture was stirred at 20 °C for 1 h. The reaction mixture was concentrated under reduced pressure. Compound 2-chloro-6-fluoro-pyridine-3-carbonyl chloride (300 mg, crude) was obtained as a yellow oil.

- <u>Step 2: (3, 5-dimethylphenyl) 2-chloro-6-fluoro-pyridine-3-carboxylate</u>

To a solution of 3, 5-dimethylphenol (90 mg, 736 µmol, 1.0 eq) in DCM (1 mL) was added TEA (111 mg, 1.10 mmol, 153 µL, 1.5 eq) and 2-chloro-6-fluoro-pyridine-3-carbonyl chloride (158 mg, 814 µmol, 1.1 eq) at 0 °C. The mixture was stirred at 20 °C for 1 h. The reaction mixture was poured into H_2O (5 mL) and extracted with DCM (5 mL*3). The combined organic layers were washed with brine (5 mL), dried over Na_2SO_4, filtered and concentrated under reduced pressure to give a residue, which was purified by column chromatography (SiO_2, Commercial hexanes/Ethyl acetate = 100/1 to 1/1). Compound (3, 5-dimethylphenyl) 2-chloro-6-fluoro-pyridine-3-carboxylate (75 mg, 268 µmol, 36% yield, 100% purity) was obtained as a white solid. MS (m/z)

(M+H+): found 280.0, calculated 280.0. ^{1}H NMR (400 MHz, DMSO-d_6) δ (ppm): 8.7-8.8 (m, 1H), 7.5-7.5 (m, 1H), 6.98 (s, 1H), 6.94 (s, 2H), 2.3-2.3 (s, 6H).

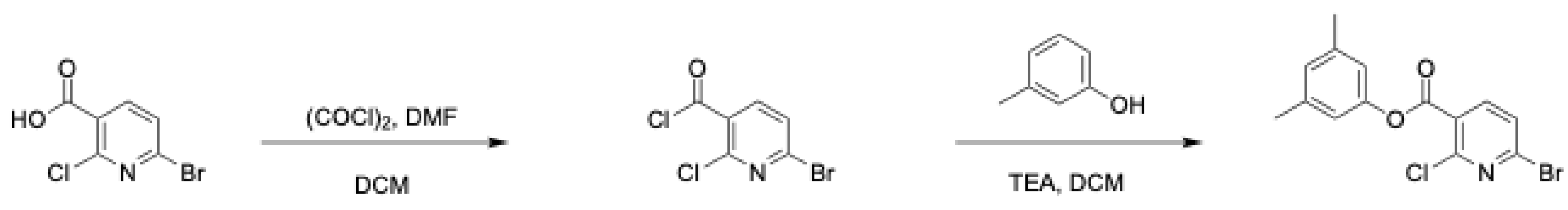

HY-2A

(3, 5-dimethylphenyl) 6-bromo-2-chloro-pyridine-3-carboxylate (HY-2A)

HY-2A was synthesized similarly as HY-1A. MS (m/z) ($M+H^+$): found 340.0, calculated 340.0. ^{1}H NMR (400 MHz, DMSO-d_6) δ (ppm): δ 8.44-8.42 (d, 1H, J=8 Hz), 7.95-7.93 (d, 1H, J=8 Hz), 6.98-6.94 (m, 3H), 2.31 (s, 6H).

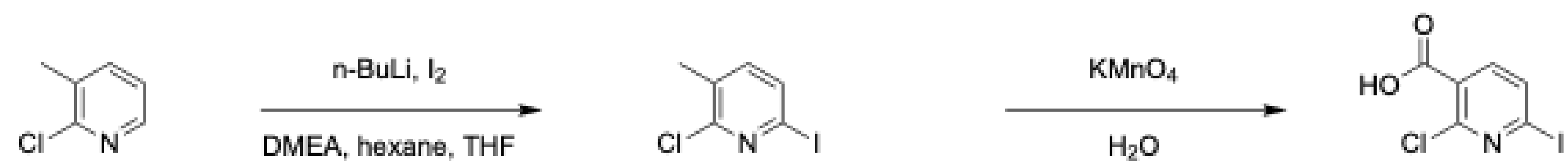

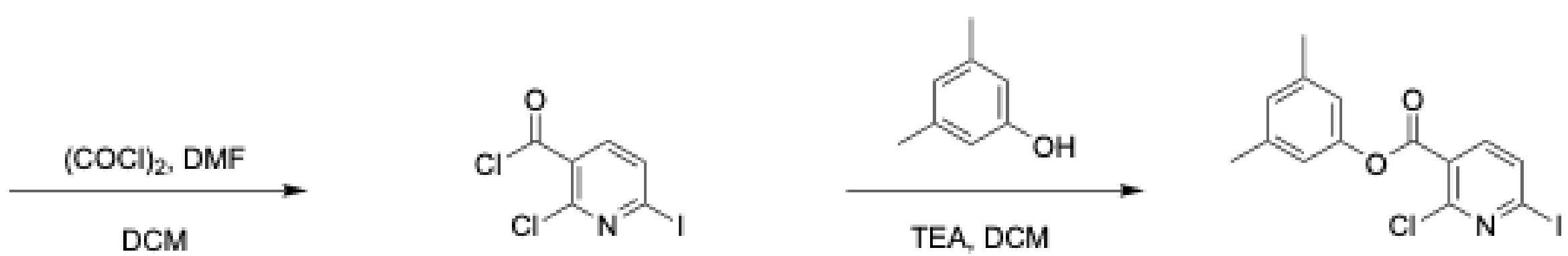

HY-3A

(3, 5-dimethylphenyl) 2-chloro-6-iodo-pyridine-3-carboxylate (HY-3A)

- Step 1: 2-chloro-6-iodo-3-methyl-pyridine

A mixture of 2-(dimethylamino)ethanol (9.5 g, 107 mmol, 10.7 mL, 2.7 eq) in hexane (60 mL) was degassed and purged with N_2 for 3 times. The mixture was cooled to -5 °C. Then n-BuLi (1.6 M, 147 mL, 6.0 eq) was added dropwise into the above mixture dropwise at -5 °C under N_2 atmosphere and stirred at -5 °C for 45 mins. Then the mixture was cooled to -75 °C. A solution of 2-chloro-3-methyl-pyridine (5 g, 39.2 mmol, 4.3 mL, 1.0 eq) in hexane (60 mL) was added dropwise into the above mixture at -75 °C and stirred at -75 °C for 2 hrs. Then a solution of I_2 (40 g, 158 mmol, 31.7 mL, 4.0 eq) in THF (270 mL) was added into the above mixture dropwise at -75 °C and stirred at -75 °C for 2 hrs. Then the mixture was warmed to 20 °C and stirred at 20 °C

for 12 hrs. The reaction mixture was cooled to 0 °C and water (100 mL) was added dropwise. The resulting mixture was extracted with EtOAc (100 mL*3). The organic layers were combined, dried over magnesium sulfate, filtered and concentrated under vacuo. The residue was purified by column chromatography (SiO_2, Commercial hexanes/Ethyl acetate = 100/1 to 1/1). Compound 2-chloro-6-iodo-3-methyl-pyridine (8 g, 31.6 mmol, 80% yield) was obtained as a yellow solid. ^{1}H NMR (400 MHz, Chloroform-*d*) δ (ppm): 7.58 - 7.56 (d, 1H, J=8 Hz), 7.20 - 7.18 (d, 1H, J=8 Hz), 2.33 (s, 3H).

- Step 2: 2-chloro-6-iodo-pyridine-3-carboxylic acid

To a solution of 2-chloro-6-iodo-3-methyl-pyridine (8 g, 31.6 mmol, 1.0 eq) in H_2O (128 mL) was added $KMnO_4$ (12 g, 75.9 mmol, 2.4 eq) at 20 °C. The mixture was stirred at 100 °C for 18 hrs. The mixture was cooled to room temperature and filtered. The filtrate was extracted with MTBE (10 mL). The aqueous layer was acidified with HCl (1M) to pH = 2 and extracted with EtOAc (50 mL*3). The combined organic layers were washed with brine (100 mL), dried over Na_2SO_4, filtered and concentrated under reduced pressure to give compound 2-chloro-6-iodo-pyridine-3-carboxylic acid (1.85 g, 6.53 mmol, 20% yield) was obtained as a white solid.

- Step 3: 2-chloro-6-iodo-pyridine-3-carbonyl chloride

To a solution of 2-chloro-6-iodo-pyridine-3-carboxylic acid (450 mg, 1.59 mmol, 1.0 eq) in DCM (5 mL) was added $(COCl)_2$ (486 mg, 3.83 mmol, 335 µL, 2.4 eq) and DMF (12 mg, 159 µmol, 12.2 µL, 0.1 eq) at 0 °C. The mixture was stirred at 20 °C for 0.5 h. The reaction mixture was concentrated under reduced pressure to give compound 2-chloro-6-iodo-pyridine-3-carbonyl chloride (470 mg, crude) was obtained as a yellow oil.

- Step 4: (3, 5-dimethylphenyl) 2-chloro-6-iodo-pyridine-3-carboxylate

To a solution of 2-chloro-6-iodo-pyridine-3-carbonyl chloride (435 mg, 1.44 mmol, 1.1 eq) in DCM (5 mL) was added TEA (663 mg, 6.55 mmol, 911 µL, 5.0 eq) and 3, 5-dimethylphenol (160 mg, 1.31 mmol, 1.0 eq) at 0 °C. The mixture was stirred at 20 °C for 1 h. The rection on ET89907-1136 (18 mg the staring material) was combined to this reaction for work-up. The combined reaction mixture was poured into H_2O (15 mL), and extracted with DCM (10 mL*3). The combined organic layers were washed with brine (15 mL), dried over Na_2SO_4, filtered and concentrated under reduced pressure to give a residue, which was purified by prep-HPLC (column: Phenomenex luna C18 100*40mm*5 um;mobile phase: [H_2O(0.04% HCl)-ACN];gradient:50%-80% B over 8.0 min). Compound (3, 5-dimethylphenyl) 2-chloro-6-iodo-pyridine-3-carboxylate

(278 mg, 717 μmol, 54% yield, 100% purity, as HCl salt) was obtained as a yellow oil. MS (m/z) ($M+H^+$): found 388.0, calculated 388.6. ^1H NMR (400 MHz, DMSO-d_6) δ (ppm): 8.18 - 8.08 (m, 2H), 7.00 - 6.87 (m, 3H), 2.31(s, 6H).

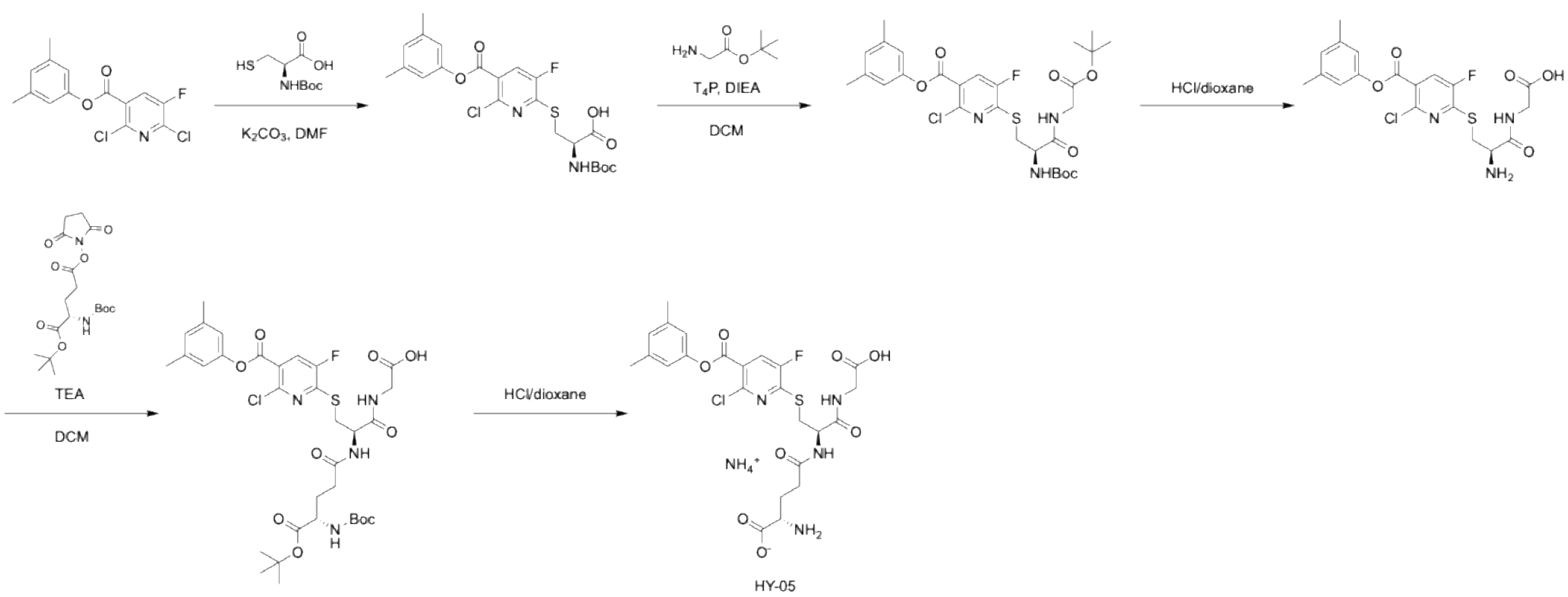

(2*S*)-2-amino-5-[[(1*R*)-2-(carboxymethylamino)-1-[[6-chloro-5-(3,5-dimethylphenoxy)carbonyl-3-fluoro-2-pyridyl]sulfanylmethyl]-2-oxo-ethyl]amino]-5-oxo-pentanoic acid (HY-05)

- Step 1: (2*R*)-2-(*tert*-butoxycarbonylamino)-3-[[6-chloro-5-(3,5-dimethylphenoxy)carbonyl-3-fluoro-2-pyridyl]sulfanyl]propanoic acid

To the mixture of (2*R*)-2-(*tert*-butoxycarbonylamino)-3-sulfanyl-propanoic acid (2.11 g, 9.55 mmol, 1 *eq*) in DMF (60 mL) was added K_2CO_3 (3.96 g, 28.65 mmol, 3 *eq*) and (3,5-dimethylphenyl)2,6-dichloro-5-fluoro-pyridine-3-carboxylate (3 g, 9.55 mmol, 1 *eq*). The mixture was stirred at 20 °C for 3 h. The residue was poured into water (100 mL) and extracted with ethyl acetate (100 mL). The aqueous phase was adjusted to pH=3 with HCl (2 N) and extracted with ethyl acetate (100 mL x 3). The combined organic phase was washed with brine (200 mL x 2), dried with anhydrous Na_2SO_4, filtered and concentrated in vacuum to obtain title compound (2.4 g, 3.75 mmol, 77.99% purity, Yield: 39.28%) as a yellow solid. MS (m/z) ($M+H^+$): found 443.0, calculated 498.1.

- Step 2: (3,5-dimethylphenyl) 6-[(2*R*)-2-(*tert*-butoxycarbonylamino)-3-[(2-*tert*-butoxy-2-oxo-ethyl)amino]-3-oxo-propyl]sulfanyl-2-chloro-5-fluoro-pyridine-3-carboxylate

To the mixture of (2*R*)-2-(*tert*-butoxycarbonylamino)-3-[[6-chloro-5-(3,5-dimethylphenoxy) carbonyl-3-fluoro-2-pyridyl]sulfanyl]propanoic acid (1 g, 2.00 mmol, 1 *eq*) in DCM (20 mL) was added T_4P (2.17 g, 3.01 mmol, 50% purity, 1.5 *eq*), DIEA (777.08 mg, 6.01 mmol, 1.05 mL, 3 *eq*) and *tert*-butyl 2-aminoacetate (262.90 mg, 2.00 mmol, 1 *eq*). The mixture was stirred at 20 °C for 12 h. The residue was poured into water (20 mL) and extracted with DCM (20 mL x 3). The combined organic phase was washed with brine (50 mL x 2), dried with anhydrous Na_2SO_4, filtered and concentrated in vacuum. The residue was purified by flash silica gel chromatography (ISCO®; 12 g SepaFlash® Silica Flash Column, Eluent of 0~50% Ethyl acetate/Hexane gradient @ 40 mL/min) to obtain the title compound (750 mg, 1.02 mmol, 50.74% yield, 83% purity) as a white solid. MS (m/z) ($M+H^+$): found 612.1, calculated 612.1. ^1H NMR (400 MHz, DMSO-d_6) δ (ppm): 8.36 (d, *J* = 9.2 Hz, 1H), 8.28 (t, *J* = 5.6 Hz, 1H), 7.20 (d, *J* = 8.8 Hz, 1H), 6.97 (s, 1H), 6.92 (s, 2H), 4.41 - 4.26 (m, 1H), 3.80 - 3.69 (m, 3H), 3.39 - 3.33 (m, 1H), 2.30 (s, 6H), 1.41 (s, 9H), 1.37 (s, 9H).

- Step 3: 2-[[(2*R*)-2-amino-3-[[6-chloro-5-(3,5-dimethylphenoxy)carbonyl-3-fluoro-2-pyridyl] sulfanyl]propanoyl]amino]acetic acid

The mixture of (3,5-dimethylphenyl)6-[(2*R*)-2-(*tert*-butoxycarbonylamino)-3-[(2-*tert*-butoxy-2-oxo-ethyl)amino]-3-oxo-propyl]sulfanyl-2-chloro-5-fluoro-pyridine-3-carboxylate (750 mg, 1.23 mmol, 1 *eq*) in HCl/dioxane (4M, 10 mL) was stirred at 20 °C for 2 h. The residue was concentrated in vacuum to obtain the title compound (600 mg, 1.01 mmol, 83.07% purity, Yield: 82.62%, HCl salt) as a yellow solid. ^1H NMR (400 MHz, DMSO-d_6) δ (ppm): 12.8 (s, 1H), 8.97 (t, *J* = 5.6 Hz, 1H), 8.62 (m, 4H), 8.41 (d, *J* = 9.2 Hz, 1H), 6.98 (s, 1H), 6.93 (s, 2H), 4.28 (m, 1H), 3.83 – 3.80 (m, 3H), 3.76 - 3.70 (m, 2H), 3.38 (s, 2H), 2.31 (s, 6H).

- Step 4: 2-[[(2*R*)-2-[[(4*S*)-5-*tert*-butoxy-4-(*tert*-butoxycarbonylamino)-5-oxo-pentanoyl] amino]-3-[[6-chloro-5-(3,5-dimethylphenoxy)carbonyl-3-fluoro-2-pyridyl]sulfanyl]propanoyl]amino]acetic acid

To the mixture of O1-*tert*-butyl O5-(2,5-dioxopyrrolidin-1-yl)(2*S*)-2-(tert-butoxycarbonylamino) pentanedioate (40.66 mg, 101.55 µmol, 1 *eq*) in DCM (2 mL) was added TEA (51.38 mg, 507.77 µmol, 70.68 µL, 5 *eq*) and 2-[[(2*R*)-2-amino-3-[[6-chloro-5-(3,5-dimethylphenoxy)carbonyl-3-fluoro-2-pyridyl]sulfanyl]propanoyl]amino]acetic acid (50 mg, 101.55 µmol, 1 *eq*, HCl). The mixture was stirred at 20 °C for 2 h. The residue was concentrated in vacuum to obtain the title

compound (70 mg, 94.44 μmol, 92.99% yield) as a yellow oil. MS (m/z) ($M+H^+$): found 741.2, calculated 741.2.

- Step 5: (2*S*)-2-amino-5-[[(1*R*)-2-(carboxymethylamino)-1-[[6-chloro-5-(3,5-dimethylphenoxy)carbonyl-3-fluoro-2-pyridyl]sulfanylmethyl]-2-oxo-ethyl]amino]-5-oxo-pentanoic acid

The mixture of 2-[[(2*R*)-2-[[(4*S*)-5-tert-butoxy-4-(tert-butoxycarbonylamino)-5-oxo-pentanoyl]-amino]-3-[[6-chloro-5-(3,5-dimethylphenoxy)carbonyl-3-fluoro-2-pyridyl]sulfanyl]propanoyl]-amino]acetic acid (70 mg, 94.44 μmol, 1 *eq*) in HCl/dioxane (4M, 2 mL) was stirred at 20 °C for 12 h. The residue was concentrated in vacuum. The residue was purified by prep-HPLC (column: 2_Phenomenex Gemini C18 75 * 40 mm * 5 um; mobile phase: [H_2O (10 mM NH_4HCO_3)-ACN]; gradient:10%-40% B over 8.0 min) to obtain the title compound (15.0 mg, 25.47 μmol, 99.33% purity, Yield: 26.97%, $NH4^+$ salt) as a white solid. MS (m/z) ($M+H^+$): found 585.1, calculated 585.1. ^{1}H NMR (400 MHz, DMSO-d_6) δ (ppm): 8.71(d, J = 0.8 Hz, 1H), 8.35 (d, J = 8.9 Hz, 2H), 6.97 (s, 1H), 6.93 (s, 2H), 4.62 (m, 1H), 3.82 (m, 1H), 3.55 (t, J = 4.8 Hz, 2H), 3.40 - 3.35 (m, 2H), 3.25 (m, 2H), 2.37 - 2.31 (m, 2H), 2.31 (s, 6H), 1.95 - 1.79 (m, 2H).

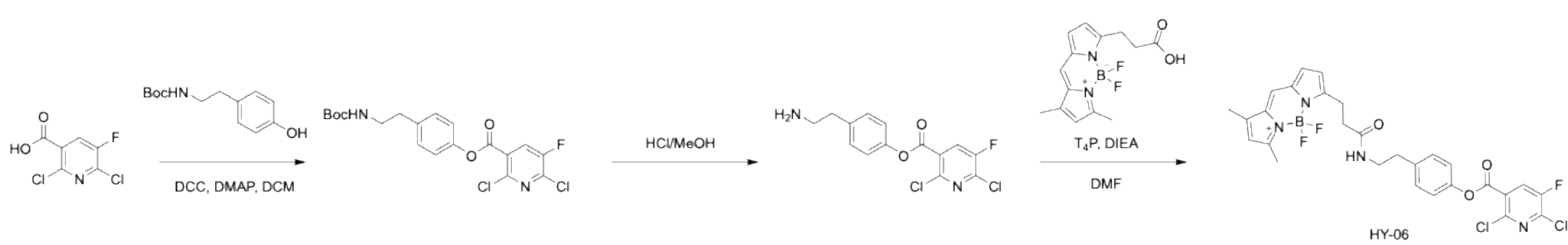

[4-[2-[3-(2,2-difluoro-10,12-dimethyl-3-aza-1-azonia-2-boranuidatricyclo[7.3.0.03,7] dodeca-1(12),4,6,8,10-pentaen-4-yl)propanoylamino]ethyl]phenyl] 2,6-dichloro-5-fluoro-pyridine-3-carboxylate (HY-06)

- Step 1: [4-[2-(*tert*-butoxycarbonylamino)ethyl]phenyl]2,6-dichloro-5-fluoro-pyridine-3-carboxylate

To the mixture of 2,6-dichloro-5-fluoro-pyridine-3-carboxylic acid (1 g, 4.76 mmol, 1 eq) and tert-butyl *N*-[2-(4-hydroxyphenyl)ethyl]carbamate (1.13 g, 4.76 mmol, 1 eq) in DCM (20 mL) was added DCC (1.47 g, 7.14 mmol, 1.44 mL, 1.5 eq) and DMAP (58.18 mg, 476.21 μmol, 0.1 eq). The mixture was stirred at 20 °C for 2 h. The residue was filtered and concentrated in vacuum. The residue was purified by flash silica gel chromatography (ISCO®; 4 g SepaFlash® Silica Flash Column, Eluent of 0~15% Ethyl acetate/Commercial hexanes gradient @ 30 mL/min) to obtain title compound (1.6 g, 3.73 mmol, 78.27% yield) as a white solid. MS (m/z) ($M+H^+$): found 372.9,

calculated 428.0. ^{1}H NMR (400 MHz, DMSO-d_6) δ (ppm): 8.73 (d, J = 8.0 Hz, 1H), 7.31 (d, J = 8.4 Hz, 2H), 7.24 (d, J = 8.4 Hz, 2H), 6.91 (t, J = 5.2 Hz, 1H), 3.19 - 3.14 (q, J = 6.8 Hz, 2H), 2.73 (t, J = 7.2 Hz, 2H), 1.37 (s, 9H).

- Step 2: [4-(2-aminoethyl)phenyl] 2,6-dichloro-5-fluoro-pyridine-3-carboxylate

The mixture of [4-[2-(*tert*-butoxycarbonylamino)ethyl]phenyl] 2,6-dichloro-5-fluoro-pyridine-3-carboxylate (600 mg, 1.33 mmol, 1 eq) in HCl/dioxane (5 mL) was stirred at 20 °C for 2 h. The residue was filtered out to title compound (450 mg, 1.23 mmol, 92.69% yield, HCl) as a white solid. MS (m/z) ($M+H^+$): found 329.0, calculated 328.0. ^{1}H NMR (400 MHz, DMSO-d_6) δ (ppm): 8.75 (d, J = 8.0 Hz, 1H), 8.17 (s, 3H), 740 (d, J = 8.4 Hz, 2H), 7.30 (d, J = 8.4 Hz, 2H), 3.19 - 3.14 (q, J = 6.0 Hz, 2H), 2.73 (t, J = 8.4 Hz, 2H).

- Step 3: [4-[2-[3-(2,2-difluoro-10,12-dimethyl-3-aza-1-azonia-2-boranuidatricyclo[7.3.0.03,7] dodeca-1(12),4,6,8,10-pentaen-4-yl)propanoylamino]ethyl]phenyl] 2,6-dichloro-5-fluoro-pyridine-3-carboxylate

To the mixture of [4-(2-aminoethyl)phenyl] 2,6-dichloro-5-fluoro-pyridine-3-carboxylate (62.59 mg, 171.18 µmol, 1 *eq*, HCl) in DMF (3 mL) was added 3-(2,2-difluoro-10,12-dimethyl-3-aza-1-azonia-2-boranuidatricyclo[7.3.0.03,7]dodeca-1(12),4,6,8,10-pentaen-4-yl)propanoic acid (50 mg, 171.18 µmol, 1 *eq*), DIEA (176.99 mg, 1.37 mmol, 238.53 µL, 8 *eq*) and T_4P (185.01 mg, 256.77 µmol, 50% purity, 1.5 *eq*). The mixture was stirred at 20 °C for 1 h. The mixture was combined with another batch (20 mg). The residue was poured into water (30 mL) and extracted with ethyl acetate (30 mL x 3). The combined organic phase was washed with brine (100 mL x 2), dried with anhydrous Na_2SO_4, filtered and concentrated in vacuum. The residue was purified by prep-HPLC (column: WePure Biotech XP tC18 150 * 40 * 10 um; mobile phase: [H_2O (10mM NH_4HCO_3)-ACN]; gradient:48%-78% B over 8.0 min) to obtain title compound (97.48% purity) (58.5 mg) as a yellow solid. MS (m/z)_ ($M+H^+$): found 583.1, calculated 602.1.^{1}H NMR (400 MHz, $CDCl_3$) δ (ppm): 8.19 (d, J = 7.2 Hz, 1H), 7.22 (d, J = 8.4 Hz, 2H), 7.13-7.11 (m, 2H), 6.89 (d, J = 3.6 Hz, 1H), 6.30 (d, J = 4.0 Hz, 1H), 6.13 (s, 1H), 5.76 (s, 1H), 3.49 (q, J = 6.8 Hz, 2H), 3.27 (t, J = 7.2 Hz, 2H), 2.78 (t, J = 7.2 Hz, 2H), 2.63 (t, J = 7.2 Hz, 2H), 2.57 (s, 3H), 2.27 (s, 3H).

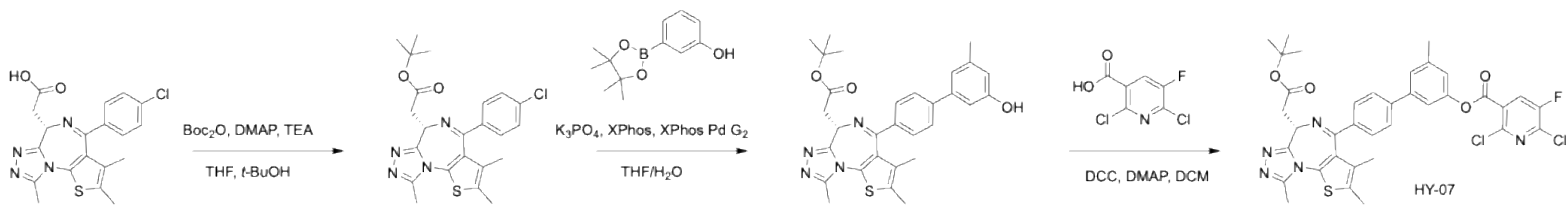

[3-[4-[(9*S*)-9-(2-tert-butoxy-2-oxo-ethyl)-4,5,13-trimethyl-3-thia-1,8,11,12-tetrazatricyclo[8.3.0.02,6]trideca-2(6),4,7,10,12-pentaen-7-yl]phenyl]-5-methyl-phenyl] 2,6-dichloro-5-fluoro-pyridine-3-carboxylate (HY-07)

- Step 1: *tert*-butyl 2-[(9*S*)-7-(4-chlorophenyl)-4,5,13-trimethyl-3-thia-1,8,11,12-tetrazatricyclo[8.3.0.02,6]trideca-2(6),4,7,10,12-pentaen-9-yl]acetate

To the mixture of 2-[(9*S*)-7-(4-chlorophenyl)-4,5,13-trimethyl-3-thia-1,8,11,12-tetrazatricyclo [8.3.0.02,6]trideca-2(6),4,7,10,12-pentaen-9-yl]acetic acid (500 mg, 1.25 mmol, 1 *eq*) in THF (10 mL) and *t*-BuOH (10 mL) was added DMAP (15.24 mg, 124.73 µmol, 0.1 *eq*), TEA (252.42 mg, 2.49 mmol, 347.20 µL, 2 *eq*) Boc_2O (326.65 mg, 1.50 mmol, 343.84 µL, 1.2 *eq*) at 50 °C. The mixture was stirred at 50 °C for 2 h. The residue was poured into water (50 mL) and extracted with ethyl acetate (50 mL*3). The combined organic phase was washed with brine (50 mL*2), dried with anhydrous Na_2SO_4, filtered and concentrated in vacuum. The residue was purified by flash silica gel chromatography (ISCO®; 12 g SepaFlash® Silica Flash Column, Eluent of 0~100% Ethyl acetate/Hexane gradient @ 40 mL/min) to obtain the title compound (200 mg, 425.70 µmol, 34.13% yield, 97.27% purity) as yellow oil. MS (m/z)_ ($M+H^+$): found 457.2, calculated 457.1. ^1H NMR (400 MHz, DMSO-d_6) δ (ppm): 7.55 - 7.47 (m, 2H), 7.46 - 7.39 (m, 2H), 5.75 (s, 1H), 4.42 (dd, J = 6.3, 8.1 Hz, 1H), 3.34 (s, 1H), 2.60 (s, 3H), 2.42 (s, 3H), 1.63 (s, 3H), 1.42 (s, 9H).

- Step 2: *tert*-butyl 2-[(9*S*)-7-[4-(3-hydroxy-5-methyl-phenyl)phenyl]-4,5,13-trimethyl-3-thia-1,8,11,12-tetrazatricyclo[8.3.0.02,6]trideca-2(6),4,7,10,12-pentaen-9-yl]acetate

To the mixture of *tert*-butyl 2-[(9*S*)-7-(4-chlorophenyl)-4,5,13-trimethyl-3-thia-1,8,11,12-tetrazatricyclo[8.3.0.02,6]trideca-2(6),4,7,10,12-pentaen-9-yl]acetate (150 mg, 328.24 µmol, 1 *eq*) in THF (6 mL) and H_2O (0.6 mL) was added K_3PO_4 (348.37 mg, 1.64 mmol, 5 *eq*) , Xphos (18.78 mg, 39.39 µmol, 0.12 *eq*), Xphos-Pd-G2 (25.83 mg, 32.82 µmol, 0.1 *eq*) and (3-hydroxy-5-methyl-phenyl)boronic acid (59.85 mg, 393.88 µmol, 1.2 *eq*). The mixture was degassed and purged with N_2 for 3 times, and then the mixture was stirred at 90 °C for 3 h. The residue was poured into water (50 mL) and extracted with ethyl acetate (50 mL*3). The combined organic phase was washed with brine (50 mL*2), dried with anhydrous Na_2SO_4, filtered and concentrated in vacuum. The residue was purified by flash silica gel chromatography (ISCO®; 4 g SepaFlash® Silica Flash

Column, Eluent of 0~100% Ethyl acetate/Hexane gradient @ 40 mL/min) to obtain the title compound (150 mg) as a white solid. MS (m/z) ($M+H^+$): found 529.3, calculated 529.2. ^{1}H NMR (400 MHz, DMSO-d_6) δ (ppm): 9.43 (s, 1H), 7.64 (d, J = 8.5 Hz, 2H), 7.48 (d, J = 8.3 Hz, 2H), 6.93 (s, 1H), 6.84 (s, 1H), 6.61 (s, 1H), 4.43 (t, J = 7.3 Hz, 1H), 3.36 - 3.32 (m, 2H), 2.61 (s, 3H), 2.43 (s, 3H), 2.28 (s, 3H), 1.68 (s, 3H), 1.44 (s, 9H).

- Step 3: [3-[4-[(9*S*)-9-(2-tert-butoxy-2-oxo-ethyl)-4,5,13-trimethyl-3-thia-1,8,11,12-tetrazatricyclo[8.3.0.02,6]trideca-2(6),4,7,10,12-pentaen-7-yl]phenyl]-5-methyl-phenyl] 2,6-dichloro-5-fluoro-pyridine-3-carboxylate

To the mixture of 2,6-dichloro-5-fluoro-pyridine-3-carboxylic acid (39.72 mg, 189.16 µmol, 1 *eq*) in DCM (2 mL) was added DCC (58.54 mg, 283.73 µmol, 57.39 µL, 1.5 *eq*), DMAP (2.31 mg, 18.92 µmol, 0.1 *eq*) and *tert*-butyl 2-[(9*S*)-7-[4-(3-hydroxy-5-methyl-phenyl)phenyl]-4,5,13-trimethyl-3-thia-1,8,11,12-tetrazatricyclo[8.3.0.02,6]trideca-2(6),4,7,10,12-pentaen-9-yl]acetate (100 mg, 189.16 µmol, 1 *eq*). The mixture was stirred at 20 °C for 2 h. The residue was filtered and concentrated in vacuum. The residue was purified by prep-TLC (SiO_2, Hexane/Ethyl acetate=0/1, Rf=0.43) to obtain the title compound (24.2 mg, 31.80 µmol, 16.81% yield, 94.70% purity) as a white solid. MS (m/z) ($M+H^+$): found 720.1, calculated 720.1. ^{1}H NMR (400 MHz, DMSO-d_6) δ (ppm): 8.77 (d, J = 8.0 Hz, 1H), 7.77 (d, J = 8.5 Hz, 2H), 7.54 (d, J = 5.1 Hz, 2H), 7.51 (s, 2H), 7.20 (s, 1H), 4.44 (t, J = 7.3 Hz, 1H), 3.36 - 3.32 (m, 2H), 2.62 (s, 3H), 2.43 (s, 6H), 1.67 (s, 3H), 1.44 (s, 9H).

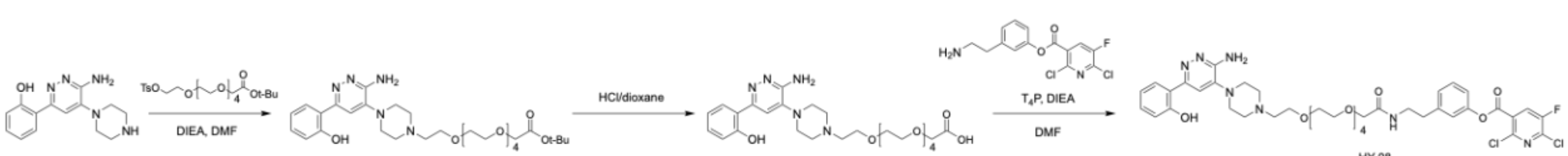

[3-[2-[[2-[2-[2-[2-[2-[2-[4-[3-amino-6-(2-hydroxyphenyl)pyridazin-4-yl]piperazin-1-yl]ethoxy]ethoxy]ethoxy]ethoxy]ethoxy]acetyl]amino]ethyl]phenyl] 2,6-dichloropyridine-3-carboxylate (HY-08)

- Step 1: *tert*-butyl 2-[2-[2-[2-[2-[2-[4-[3-amino-6-(2-hydroxyphenyl)pyridazin-4-yl]piperazin-1-yl]ethoxy]ethoxy]ethoxy]ethoxy]ethoxy]acetate

To a solution of 2-(6-amino-5-piperazin-1-yl-pyridazin-3-yl)phenol (50 mg, 162.45 µmol, 1 eq, HCl) in DMF (3 mL) was added DIEA (104.98 mg, 812.27 µmol, 141.48 µL, 5 eq) and *tert*-butyl 2-[2-[2-[2-[2-(*p*-tolylsulfonyloxy)ethoxy]ethoxy]ethoxy]ethoxy]acetate (82.30 mg, 162.45 µmol, 1 eq). The mixture was stirred at 90 °C for 4 h. To the mixture was added tert-butyl

2-[2-[2-[2-[2-[2-(p-tolylsulfonyloxy)ethoxy]ethoxy]ethoxy]ethoxy]ethoxy]acetate (41.15 mg, 81.23 µmol, 0.5 eq). The mixture was stirred at 90 °C for 2 h. The reaction mixture was quenched by addition H_2O 10 mL and extracted with EtOAc 30 mL (10 mL x 3). The combined organic layers were concentrated under reduced pressure to give a residue. The residue was purified by p-TLC (SiO_2, Ethyl acetate: Methanol=5:1) to obtain title compound (20 mg, 29.72 µmol, 18.29% yield, 90% purity) as a yellow oil. MS (m/z) ($M+H^+$): found 606.5, calculated 605.3.

- <u>Step 2: 2-[2-[2-[2-[2-[2-[4-[3-amino-6-(2-hydroxyphenyl)pyridazin-4-yl]piperazin-1-yl]ethoxy]ethoxy]ethoxy]ethoxy]ethoxy]acetic acid</u>

A solution of *tert*-butyl 2-[2-[2-[2-[2-[2-[4-[3-amino-6-(2-hydroxyphenyl)pyridazin-4-yl]pipera zin-1-yl]ethoxy]ethoxy]ethoxy]ethoxy]ethoxy]acetate (20 mg, 33.02 µmol, 1 eq) in HCl/dioxane (4 mL) was stirred at 20 °C for 3 hrs. The reaction mixture was filtered and concentrated under reduced pressure to give the title compound (20 mg, crude, HCl) as a white solid.

- <u>Step 3: [3-[2-[[2-[2-[2-[2-[2-[2-[4-[3-amino-6-(2-hydroxyphenyl)pyridazin-4-yl]piperazin-1-yl]ethoxy]ethoxy]ethoxy]ethoxy]ethoxy]acetyl]amino]ethyl]phenyl] 2,6-dichloropyridine-3-carboxylate</u>

To a solution of 2-[2-[2-[2-[2-[2-[4-[3-amino-6-(2-hydroxyphenyl)pyridazin-4-yl]piperazin-1-yl]ethoxy]ethoxy]ethoxy]ethoxy]ethoxy]acetic acid (18 mg, 30.71 µmol, 1 eq, HCl) and [3-(2-aminoethyl)phenyl]2,6-dichloropyridine-3-carboxylate (10.68 mg, 30.71 µmol, 1 eq, HCl) in DCM (4 mL) was added T_4P (33.19 mg, 46.07 µmol, 50% purity, 1.5 eq) and DIEA (23.82 mg, 184.28 µmol, 32.10 µL, 6 eq). The mixture was stirred at 20 °C for 2 hrs. The reaction mixture was concentrated under reduced pressure to remove solvent. The residue was purified by prep-HPLC (column: Waters Xbridge BEH C18 100 * 25 mm * 10 um; mobile phase: [H_2O (10 mM NH_4HCO_3)-ACN]; gradient:40%-75% B over 8.0 min) to obtain title compound (4.6 mg, 4.77 µmol, 15.53% yield, 87.41% purity) as a red solid. MS (m/z) ($M+H^+$): found 842.3, calculated 842.3. ^1H NMR (400 MHz, DMSO-d_6) δ (ppm): 14.24 (br s, 1H), 8.58 (d, J = 8.0 Hz, 1H), 7.94 - 7.88 (m, 1H), 7.84 - 7.74 (m, 2H), 7.49 (s, 1H), 7.44 - 7.37 (m, 1H), 7.27 - 7.14 (m, 4H), 6.92 - 6.85 (m, 2H), 6.24 (br s, 2H), 3.85 (s, 2H), 3.61 - 3.44 (m, 18H), 3.40 (m, 2H), 3.09 (m, 4H), 2.80 (t, J = 7.2 Hz, 2H), 2.66 (m, 4H), 2.55 (m, 2H).

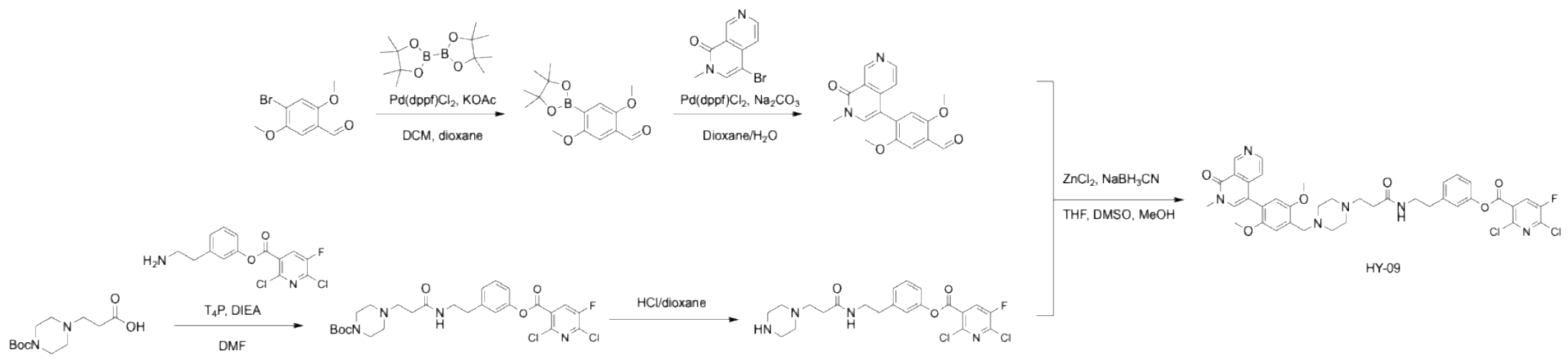

[3-[2-[3-[4-[[2,5-dimethoxy-4-(2-methyl-1-oxo-2,7-naphthyridin-4-yl)phenyl]methyl] piperazin-1-yl]propanoylamino]ethyl]phenyl] 2,6-dichloropyridine-3-carboxylate (HY-09)

- Step 1: 2,5-dimethoxy-4-(4,4,5,5-tetramethyl-1,3,2-dioxaborolan-2-yl)benzaldehyde

To the mixture of 4-bromo-2,5-dimethoxy-benzaldehyde (2 g, 8.16 mmol, 1 *eq*) in dioxane (20 mL) was added KOAc (2.40 g, 24.48 mmol, 3 *eq*), 4,4,5,5-tetramethyl-2-(4,4,5,5-tetramethyl-1,3,2-dioxaborolan-2-yl)-1,3,2-dioxaborolane (2.49 g, 9.79 mmol, 1.2 *eq*) and Pd(dppf)Cl_2.CH_2Cl_2 (333.23 mg, 408.05 μmol, 0.05 *eq*). The mixture was degassed and purged with N_2 for 3 times, and then the mixture was stirred at 90 °C for 12 h. The residue was concentrated in vacuum. The residue was purified by flash silica gel chromatography (ISCO®; 12 g SepaFlash® Silica Flash Column, Eluent of 0~30% MeOH/DCM gradient @ 40 mL/min) to obtain the title compound (2.2 g, 7.53 mmol, yield: 92.28%) as a yellow solid. ^{1}H NMR (400 MHz, DMSO-d_6) δ (ppm): 10.3 (s, 1H), 7.27 (s, 1H), 7.17 (s, 1H), 3.88 (s, 3H), 3.73 (s, 3H), 1.30 (s, 12H).

- Step 2: 2,5-dimethoxy-4-(2-methyl-1-oxo-2,7-naphthyridin-4-yl)benzaldehyde

To the mixture of 4-bromo-2-methyl-2,7-naphthyridin-1-one (300 mg, 1.25 mmol, 1 *eq*) in dioxane (8 mL) and H_2O (2 mL) was added 2,5-dimethoxy-4-(4,4,5,5-tetramethyl-1,3,2-dioxaborolan-2-yl)benzaldehyde (549.89 mg, 1.88 mmol, 1.5 *eq*), Na_2CO_3 (399.01 mg, 3.76 mmol, 3 *eq*) and Pd(dppf)Cl_2 (45.91 mg, 62.74 μmol, 0.05 *eq*). The mixture was stirred at 100 °C for 2 h. The residue was poured into water (100 mL) and extracted with DCM (100 mL x 3). The combined organic phase was washed with brine (200 mL x 2), dried with anhydrous Na_2SO_4, filtered and concentrated in vacuum. The crude product was triturated with MTBE (20 mL) at 20 ^{o}C for 30 min. The brown precipitate was collected in a Büchner funnel under suction filtration, and concentrated under reduced pressure to obtain the title compound (300 mg, 749.24 μmol, 59.71% yield, 81% purity) as a brown solid. MS (m/z) (M+H^+): found 325.2, calculated 325.2. ^{1}H NMR (400 MHz, DMSO-d_6) δ (ppm): 10.4 (s, 1H), 9.42 (s, 1H), 8.65 (d, J = 5.6 Hz, 1H), 7.85 (s, 1H), 7.37 (s, 1H), 7.26 (s, 1H), 7.04 (d, J = 5.6 Hz, 1H), 3.92 (s, 3H), 3.70 (s, 3H), 3.59 (s, 3H).

- Step 3: *tert*-butyl 4-[3-[2-[3-(2,6-dichloropyridine-3-carbonyl)oxyphenyl]ethylamino]-3-oxo-propyl]piperazine-1-carboxylate

To a solution of 3-(4-tert-butoxycarbonylpiperazin-1-yl)propanoic acid (100 mg, 387.13 µmol, 1 *eq*) in DCM (4 mL) was added T_4P (418.40 mg, 580.69 µmol, 50% purity, 1.5 *eq*) and DIPEA (150.10 mg, 1.16 mmol, 202.29 µL, 3 *eq*) and [3-(2-aminoethyl)phenyl] 2,6-dichloropyridine-3-carboxylate (134.57 mg, 387.13 µmol, 1 *eq*, HCl). The mixture was stirred at 20 °C for 2 h. The reaction mixture was quenched by addition H_2O 20 mL and extracted with EtOAc (20 mL x 4). The combined organic layers were dried over Na_2SO_4, filtered and concentrated under reduced pressure to obtain the title compound (180 mg, 326.41 µmol, yield: 84.31%) as a yellow oil. ^1H NMR (400 MHz, MeOH) δ (ppm): 8.50 (d, J = 8.0 Hz, 1H), 7.64 (d, J = 5.2 Hz, 1H), 7.38 (t, J = 8.0 Hz, 1H), 7.22 (d, J = 7.6 Hz, 1H), 7.16 (s, 1H), 7.13 (d, J = 8.0 Hz, 1H), 3.52 - 3.48 (m, 6H), 3.01 (s, 2H), 2.88 (t, J = 6.8 Hz, 2H), 2.86 - 2.82 (m, 4H), 2.57 -2.53 (m, 2H), 1.45 (s, 9H).

- Step 4: [3-[2-(3-piperazin-1-ylpropanoylamino)ethyl]phenyl] 2,6-dichloropyridine-3-carboxylate

A solution of *tert*-butyl 4-[3-[2-[3-(2,6-dichloropyridine-3-carbonyl)oxyphenyl]ethylamino]-3-oxo-propyl]piperazine-1-carboxylate (180 mg, 326.41 µmol, 1 *eq*) in HCl/dioxane (4M, 5 mL) was stirred at 20 °C for 2 h. The reaction mixture was concentrated under reduced pressure to remove solvent. The crude product was triturated with EtOAc (3 mL) at 20 °C for 2 min. The white precipitate was collected in a Büchner funnel under suction filtration, and concentrated under reduced pressure to obtain the title compound (120 mg, 265.87 µmol, yield: 81.45%, HCl salt) as a white solid. ^1H NMR (400 MHz, MeOH) δ (ppm): 8.51 (d, J = 8.0 Hz, 1H), 7.64 (d, J = 8.0 Hz, 1H), 7.40 (t, J = 7.6 Hz, 1H), 7.22 (d, J = 7.6 Hz, 1H), 7.17 (s, 1H), 7.13 (d, J = 8.0 Hz,1H), 3.59 - 3.31 (m, 12H), 2.88 (t, J = 6.8 Hz, 2H), 2.76 (t, J = 6.8 Hz, 2H).

- Step 5: [3-[2-[3-[4-[[2,5-dimethoxy-4-(2-methyl-1-oxo-2,7-naphthyridin-4-yl)phenyl]methyl] piperazin-1-yl]propanoylamino]ethyl]phenyl] 2,6-dichloropyridine-3-carboxylate

To the mixture of 2,5-dimethoxy-4-(2-methyl-1-oxo-2,7-naphthyridin-4-yl)benzaldehyde (20 mg, 61.67 µmol, 1 *eq*) and [3-[2-(3-piperazin-1-ylpropanoylamino)ethyl]phenyl] 2,6-dichloropyridine-3-carboxylate (27.83 mg, 61.67 µmol, 1 *eq*) in DMSO (0.5 mL) was added $ZnCl_2$ (1 M, 92.50 µL, 1.5 *eq*) in THF (0.1 mL) and stirred at 20 °C for 1 h. The mixture was added $NaBH_3CN$ (15.50 mg, 246.66 µmol, 4 *eq*) in MeOH (0.2 mL) and stirred at 20 °C for 12 h. The residue was poured

into water (10 mL) and extracted with ethyl acetate (10 mL x 3). The combined organic phase was washed with brine (20 mL x 2), dried with anhydrous Na_2SO_4, filtered and concentrated in vacuum. T he residue was purified by prep-HPLC (column: WePure Biotech XP tC18 150 * 40 * 7 um; mobile phase: [H_2O (10 mM NH_4HCO_3)-ACN]; gradient:35%-75% B over 8.0 min) to obtain the title compound (5.1 mg, 6.57 µmol, 97.87% purity, yield: 10.65%) as a white solid. MS (m/z) ($M+H^+$): found 759.2, calculated 759.2. ^1H NMR (400 MHz, DMSO-d_6) δ (ppm): 9.40 (s, 1H), 8.63 (d, *J* = 5.6 Hz, 1H), 8.58 (d, *J* = 8.4 Hz, 1H), 8.05 (t, *J* = 5.6 Hz, 1H), 7.81 (d, *J* = 8.0 Hz, 1H), 7.73 (s, 1H), 7.45 - 7.38 (m, 1H), 7.23 - 7.15 (m, 3H), 7.11 (s, 1H), 7.03 (d, *J* = 5.6 Hz, 1H), 6.92 (s, 1H), 3.74 (s, 3H), 3.62 (s, 3H), 3.57 (s, 3H), 3.50 (s, 2H), 3.35 - 3.32 (m, 2H), 3.32 - 3.28 (m, 4H), 2.77 (t, *J* = 6.8 Hz, 2H), 2.48 - 2.34 (m, 6H), 2.22 (t, J = 7.2 Hz, 2H).

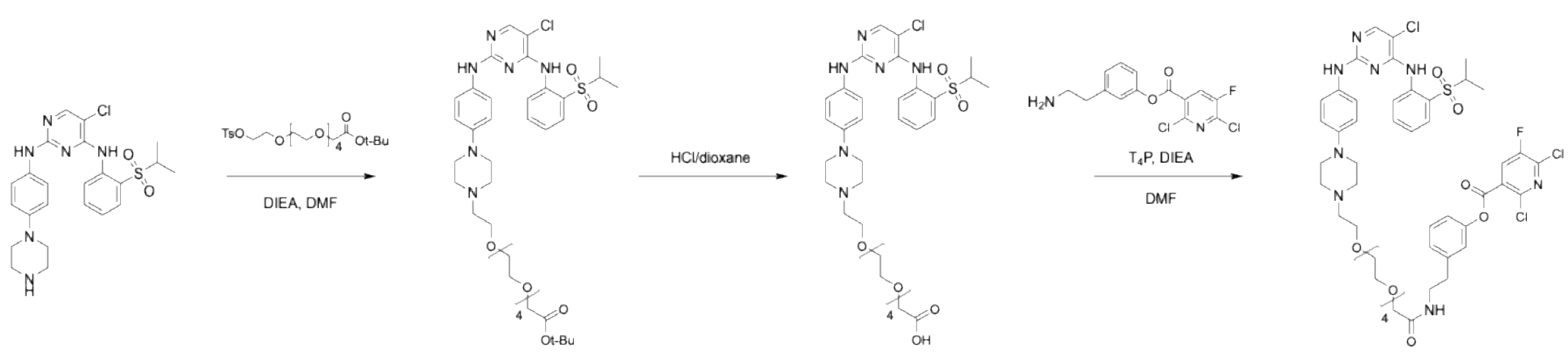

HY-10

[3-[2-[[2-[2-[2-[2-[2-[2-[4-[4-[[5-chloro-4-(2-isopropylsulfonylanilino)pyrimidin-2-yl]amino]phenyl]piperazin-1-yl]ethoxy]ethoxy]ethoxy]ethoxy]ethoxy]acetyl]amino]ethyl] phenyl] 2,6-dichloropyridine-3-carboxylate (HY-10)

- Step 1: *tert*-butyl 2-[2-[2-[2-[2-[2-[4-[4-[[5-chloro-4-(2-isopropylsulfonylanilino)pyrimidin-2-yl]amino]phenyl]piperazin-1-yl]ethoxy]ethoxy]ethoxy]ethoxy]ethoxy]acetate

To the mixture of 5-chloro-N_4-(2-isopropylsulfonylphenyl)-N_2-(4-piperazin-1-ylphenyl)pyrimidine-2,4-diamine (100 mg, 191.03 µmol, 1 eq, HCl) in DMF (2 mL) was added DIEA (123.45 mg, 955.15 µmol, 166.37 µL, 5 eq) and tert-butyl 2-[2-[2-[2-[2-[2-(p-tolylsulfonyloxy) ethoxy]ethoxy] ethoxy]ethoxy]ethoxy]acetate (116.13 mg, 229.24 µmol, 1.2 eq). The mixture was stirred at 90 °C for 2 h. The residue was poured into water (20 mL) and extracted with ethyl acetate (20 mL x 3). The combined organic phase was washed with brine (50 mL x 2), dried with anhydrous Na_2SO_4, filtered and concentrated in vacuum to obtain the title compound (100 mg, 121.74 µmol, yield: 63.73%) as a brown solid. MS (m/z) ($M+H^+$): found 821.4, calculated 821.4.

- Step 2: 2-[2-[2-[2-[2-[2-[4-[4-[[5-chloro-4-(2-isopropylsulfonylanilino)pyrimidin-2-yl]amino]phenyl]piperazin-1-yl]ethoxy]ethoxy]ethoxy]ethoxy]ethoxy]acetic acid

The mixture of *tert*-butyl 2-[2-[2-[2-[2-[2-[4-[4-[[5-chloro-4-(2-isopropylsulfonylanilino)pyrimidin-2-yl]amino]phenyl]piperazin-1-yl]ethoxy]ethoxy]ethoxy]ethoxy]ethoxy]acetate (100 mg, 122 µmol, 1 *eq*) in HCl/dioxane (4M, 2 mL) was stirred at 20 °C for 1 h. The residue was poured into water (20 mL) and extracted with ethyl acetate (20 mL x 3). The combined organic phase was washed with brine (50 mL x 2), dried with anhydrous Na_2SO_4, filtered and concentrated in vacuum to obtain title compound (70 mg, 87.31 µmol, yield: 71.72%, HCl salt) as a brown oil. MS (m/z) ($M+H^+$): found 765.3, calculated 765.3.

- Step 3: [3-[2-[[2-[2-[2-[2-[2-[2-[4-[4-[[5-chloro-4-(2-isopropylsulfonylanilino)pyrimidin-2-yl]amino]phenyl]piperazin-1-yl]ethoxy]ethoxy]ethoxy]ethoxy]ethoxy]acetyl]amino]ethyl] phenyl] 2,6-dichloropyridine-3-carboxylate

To the mixture of 2-[2-[2-[2-[2-[2-[4-[4-[[5-chloro-4-(2-isopropylsulfonylanilino)pyrimidin-2-yl] amino]phenyl]piperazin-1-yl]ethoxy]ethoxy]ethoxy]ethoxy]ethoxy]acetic acid (60 mg, 74.83 µmol, 1 *eq*, HCl) in DMF (1 mL) was added HATU (42.68 mg, 112.25 µmol, 1.5 *eq*), DIEA (29.02 mg, 224.50 µmol, 39.10 µL, 3 *eq*) and [3-(2-aminoethyl)phenyl] 2,6-dichloropyridine-3-carb oxylate (26.01 mg, 74.83 µmol, 1 *eq*, HCl). The mixture was stirred at 20 °C for 0.5 h. The residue was poured into water (20 mL) and extracted with ethyl acetate (20 mL x 3). The combined organic phase was washed with brine (50 mL x 2), dried with anhydrous Na_2SO_4, filtered and concentrated in vacuum. The residue was purified by prep-HPLC (column: WePure Biotech XP tC18 150 * 40 * 7 um; mobile phase: [H_2O (10 mM NH_4HCO_3)-ACN]; gradient:57%-87% B over 8.0 min) to obtain title compound (8.2 mg, 7.47 µmol, 96.47% purity, yield: 9.99%) as a white solid. MS (m/z) ($M+H^+$): found 1059.4, calculated 1059.2. ^1H NMR (400 MHz, DMSO-d_6) δ (ppm): 9.48 (s, 1H), 9.30 (s, 1H), 8.70 - 8.60 (m, 1H), 8.58 (d, *J* = 8.0 Hz, 1H), 8.23 (s, 1H), 7.86 - 7.79 (m, 2H), 7.79 - 7.68 (m, 2H), 7.45 - 7.38 (m, 3H), 7.38 - 7.34 (m, 1H), 7.24 - 7.11 (m, 3H), 6.85 (d, J = 9.2 Hz, 2H), 3.85 (s, 2H), 3.53 (s, 6H), 3.52 - 3.48 (m, 13H), 3.46 - 3.42 (m, 1H), 3.40 - 3.34 (m, 3H), 3.04 (m, 4H), 2.80 (t, *J* = 7.2 Hz, 2H), 2.56 - 2.53 (m, 4H), 1.16 (d, *J* = 6.8 Hz, 6H).

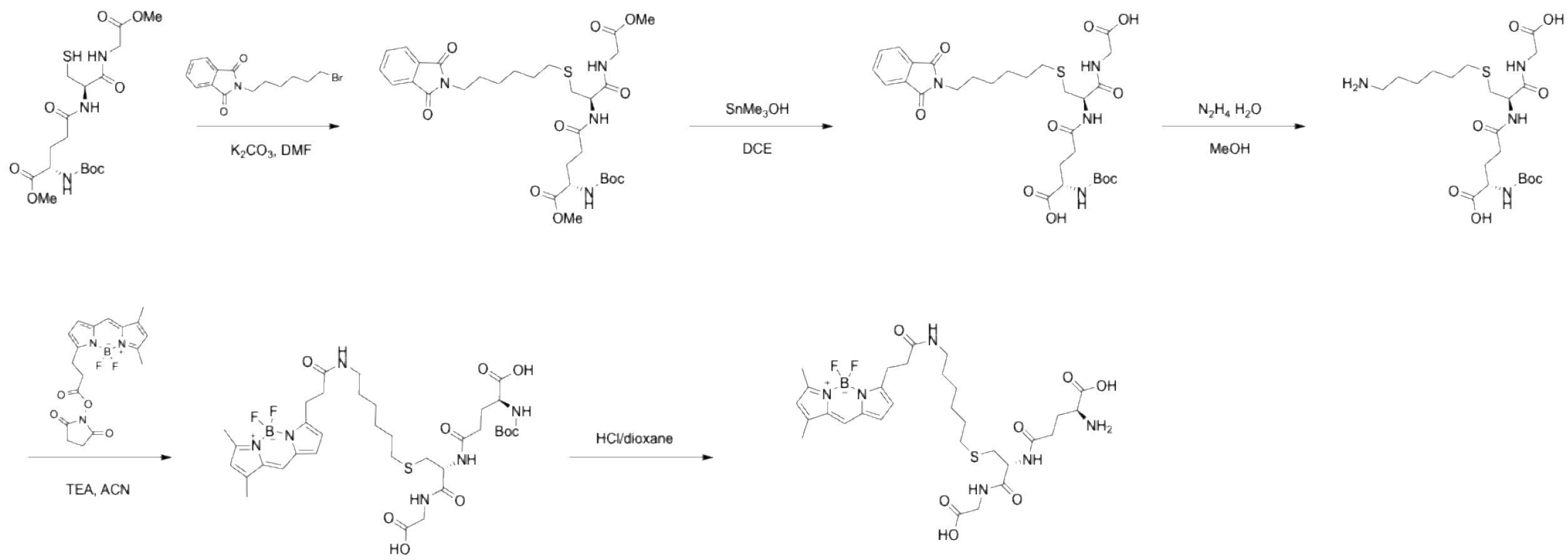

HY-11

(2S)-2-amino-5-[[(1R)-2-(carboxymethylamino)-1-[6-[3-(2,2-difluoro-10,12-dimethyl-1-aza-3-azonia-2-boranuidatricyclo[7.3.0.03,7]dodeca-3,5,7,9,11-pentaen-4-yl)propanoylamino]hexylsulfanylmethyl]-2-oxo-ethyl]amino]-5-oxo-pentanoic acid (HY-11)

- Step 1: Methyl (2S)-2-(tert-butoxycarbonylamino)-5-[[(1R)-1-[6-(1,3-dioxoisoindolin-2-yl)hexylsulfanylmethyl]-2-[(2-methoxy-2-oxo-ethyl)amino]-2-oxo-ethyl]amino]-5-oxo-pentanoate

To a solution of methyl (2S)-2-(tert-butoxycarbonylamino)-5-[[(1R)-2-[(2-methoxy-2-oxo-ethyl)amino]-2-oxo-1-(sulfanylmethyl)ethyl]amino]-5-oxo-pentanoate (500 mg, 1.15 mmol, 1.0 eq) in DMF (8 mL) was added K_2CO_3 (478 mg, 3.46 mmol, 3.0 eq) and 2-(6-bromohexyl)isoindoline-1,3-dione (534 mg, 1.72 mmol, 1.5 eq) at 20 °C. The mixture was stirred at 90 °C for 1 h. The combined mixtures were poured into H_2O (80 mL), and extracted with EtOAc (20 mL*3). The combined organic layers were washed with brine (30 mL), dried over Na_2SO_4, filtered and concentrated under reduced pressure to give a residue, which was purified by column chromatography (SiO_2, Commercial hexanes/Ethyl acetate = 100/1 to 0/1). Compound methyl (2S)-2-(tert-butoxycarbonylamino)-5-[[(1R)-1-[6-(1,3-dioxoisoindolin-2-yl)hexylsulfanylmethyl]-2-[(2-methoxy-2-oxo-ethyl)amino]-2-oxo-ethyl]amino]-5-oxo-pentanoate (1.3 g, 1.68 mmol, 73% yield, 86% purity) was obtained as a white solid. ^{1}H NMR (400 MHz, DMSO-d_6) δ (ppm): 8.48 - 8.45 (m, 1H), 8.10 - 8.08 (d, 1H, J=8 Hz), 7.89 - 7.83 (m, 4H), 7.26 - 7.24 (d, 1H, J=8 Hz), 4.46 - 4.44 (m, 1H), 1.01 - 3.98 (m, 1H), 3.85 - 3.85 (d, 2H, J=8 Hz), 3.62 (s, 6H), 3.58 - 3.55 (m, 2H), 2.83 - 2.78 (m, 1H), 2.60 - 2.55 (m, 1H), 2.49 (s, 2H), 2.25 - 2.19 (m, 2H), 1.93 - 1.90 (m, 1H), 1.78- 1.74 (m, 1H), 1.60 - 1.55 (m, 2H), 1.50 - 1.45 (m, 2H), 1.38 (s, 9H), 1.33 - 1.26 (m, 4H).

- Step 2&Step 3: (2S)-5-[[(1R)-1-(6-aminohexylsulfanylmethyl)-2-(carboxymethylamino) -2-oxo-ethyl]amino]-2-(tert-butoxycarbonylamino)-5-oxo-pentanoic acid

To a solution of methyl (2S)-2-(tert-butoxycarbonylamino)-5-[[(1R)-1-[6-(1,3-dioxoisoindolin-2-yl)hexylsulfanylmethyl]-2-[(2-methoxy-2-oxo-ethyl)amino]-2-oxo-ethyl] amino]-5-oxo-pentanoate (350 mg, 527 µmol, 1.0 eq) in DCE (4 mL) was added hydroxy(trimethyl)stannane (954 mg, 5.28 mmol, 10 eq) at 20 °C. The mixture was stirred at 90 °C for 1 hrs. The resulting mixture was cooled to 20 °C and diluted with MeOH (4 mL). Then $N_2H_4.H_2O$ (494 mg, 7.89 mmol, 478 µL, 80% purity, 15 eq) was added to the reaction mixture at 20 °C. The mixture was stirred at 70 °C for 1 h. The reaction mixture was filtered and the filter cake was washed with MeOH (5 mL). The filtrate was concentrated under reduced pressure to give a residue, which was purified by prep-HPLC (column: WePure Biotech XP tC18 100*30*7um; mobile phase: [H_2O (0.04% HCl)-ACN]; gradient: 5%-25% B over 12.0 min). Compound (2S)-5-[[(1R)-1-(6-aminohexylsulfanylmethyl)-2-(carboxymethylamino)-2-oxo-ethyl]amino]-2-(tert-butoxy carbonylamino)-5-oxo-pentanoic acid (180 mg, 355 µmol, 33% yield, 100% purity) was obtained as a white solid. ^{1}H NMR (400 MHz, DMSO-d_6) δ (ppm): 12.52 (s, 1H), 8.43 - 8.29 (m, 1H), 8.12 - 8.10 (d, 1H, J=8 Hz), 7.76 (s, 3H), 7.11-7.09 (d, J = 8.0 Hz, 1H), 4.47- 4.42 (m, 1H), 3.91 - 3.87 (m, 1H), 3.75 - 3.74 (m, 2H), 2.85 - 2.81 (m, 1H), 2.78 - 2.71 (m, 2H), 2.60 - 2.52 (m, 2H), 2.23 - 2.19 (m, 2H), 1.95 - 1.91 (m, 1H), 1.76 - 1.71 (m, 1H), 1.61 - 1.58 (m, 1H), 1.56 - 1.46 (m, 4H), 1.38 (s, 6H), 1.34 - 1.30 (m, 5H).

Step 4&Step 5: (2S)-2-amino-5-[[(1R)-2-(carboxymethylamino)-1-[6-[3-(2,2-difluoro-10,12-dimethyl-1-aza-3-azonia-2-boranuidatricyclo[7.3.0.03,7]dodeca-3,5,7,9,11-pentaen-4-yl)propanoylamino]hexylsulfanylmethyl]-2-oxo-ethyl]amino]-5-oxo-pentanoic acid (HY-11)

To a solution of (2S)-5-[[(1R)-1-(6-aminohexylsulfanylmethyl)-2-(carboxymethylamino)-2-oxo-ethyl]amino]-2-(tert-butoxycarbonylamino)-5-oxo-pentanoic acid (44 mg, 86.9 µmol, 1.0 eq) in MeCN (0.5 mL) was added TEA (44 mg, 435 µmol, 60.5 µL, 5.0 eq) and a solution of (2,5-dioxopyrrolidin-1-yl) 3-(2,2-difluoro-10,12-dimethyl-1-aza-3-azonia-2-boranuida tricyclo[7.3.0.03,7]dodeca-3,5,7,9,11-pentaen-4-yl)propanoate (35 mg, 90 µmol, 1.0 eq) in MeCN (0.5 mL) at 20 °C. The mixture was stirred at 20 °C for 1 h. Then HCl/dioxane (4 M, 429 µL, 20 eq) was added and stirred at 20 °C for 1 hr. The reaction mixture was concentrated under reduced

pressure to give a residue, which was purified by prep-HPLC (column: Phenomenex Luna C18 150*30mm*5um; mobile phase: [H_2O (0.04% HCl)-ACN]; gradient: 1%-50% B over 8.0 min). Compound (2S)-2-amino-5-[[(1R)-2-(carboxymethylamino)-1-[6-[3-(2,2-difluoro-10,12-dimethyl-1-aza-3-azonia-2-boranui datricyclo[7.3.0.03,7]dodeca-3,5,7,9,11-pentaen-4-yl)propanoylamino]hexylsulfanylmethyl] -2-oxo-ethyl]amino]-5-oxo-pentanoic acid (21 mg, 27.5 µmol, 32% yield, 94% purity, as HCl salt) was obtained as a red solid. MS (m/z) ($M+H^+$): found 681.3, calculated 681.6. ^{1}H NMR (400 MHz, Methanol-d_4) δ (ppm): 7.44 (s, 1H), 7.01 - 7.00 (d, 1H, J=4.0 Hz), 6.33 - 6.32 (d, 1H, J=4.0 Hz), 6.22 (s, 1H), 4.58 - 4.54 (m, 1H), 4.02 - 3.99 (m, 1H), 3.93 - 3.92 (m, 2H), 3.15-3.25 (m, 4H), 3.04 - 2.99 (m, 1H), 2.78 - 2.72 (m, 1H), 2.62 - 2.55 (m, 6H), 2.57 (s, 3H), 2.29 (s, 3H), 2.22 - 2.17 (m, 2H), 1.59 - 1.54 (m, 2H), 1.50 - 1.47(m, 2H), 1.42 - 1.36 (m, 2H), 1.34 - 1.29 (m, 2H).
