# Supplementary material for: DCAF11-dependent molecular glue degrader activated by glutathionylation
Source: Nature. 2026 Aug 5;657(8133):1045–52. doi: 10.1038/s41586-026-10873-1 (PMC13600927; doi:10.1038/s41586-026-10873-1)
Supplement: Supplementary file 2 [file 41586_2026_10873_MOESM2_ESM.pdf]

nature portfolio

Corresponding author(s): Benjamin L. Ebert and Eric S. Fischer

Last updated by author(s): Jun 14, 2026

Reporting Summary

Nature Portfolio wishes to improve the reproducibility of the work that we publish. This form provides structure for consistency and transparency in reporting. For further information on Nature Portfolio policies, see our Editorial Policies and the Editorial Policy Checklist.

Statistics

For all statistical analyses, confirm that the following items are present in the figure legend, table legend, main text, or Methods section.

n/a	Confirmed	
☐	☒	The exact sample size (*n*) for each experimental group/condition, given as a discrete number and unit of measurement
☐	☒	A statement on whether measurements were taken from distinct samples or whether the same sample was measured repeatedly
☐	☒	The statistical test(s) used AND whether they are one- or two-sided<br>*Only common tests should be described solely by name; describe more complex techniques in the Methods section.*
☒	☐	A description of all covariates tested
☐	☒	A description of any assumptions or corrections, such as tests of normality and adjustment for multiple comparisons
☐	☒	A full description of the statistical parameters including central tendency (e.g. means) or other basic estimates (e.g. regression coefficient) AND variation (e.g. standard deviation) or associated estimates of uncertainty (e.g. confidence intervals)
☒	☐	For null hypothesis testing, the test statistic (e.g. *F*, *t*, *r*) with confidence intervals, effect sizes, degrees of freedom and *P* value noted<br>*Give P values as exact values whenever suitable.*
☒	☐	For Bayesian analysis, information on the choice of priors and Markov chain Monte Carlo settings
☒	☐	For hierarchical and complex designs, identification of the appropriate level for tests and full reporting of outcomes
☒	☐	Estimates of effect sizes (e.g. Cohen's *d*, Pearson's *r*), indicating how they were calculated

Our web collection on statistics for biologists contains articles on many of the points above.

Software and code

Policy information about availability of computer code

Data collection	Western blot data were imaged on Odyssey Imaging System with Image Studio Software (v. 6.0, Li-COR).<br>SDS-page gel image was obtained on Gel Doc XR+ System (Bio-rad).<br>Flow data were collected on BD Symphony Cell Analyzer with BD FACSDiva 8.0 software (BD Biosciences).<br>TR-FRET data were acquired on a PHERAstar FS microplate reader (BMG Labtech).<br>Proteomics data were collected using an Orbitrap Exploris 480 mass spectrometer (Thermo Fisher Scientific) and a TimsTOF HT (Bruker).<br>qPCR data were collected using a QStudio 6 FLX Real-Time PCR System (Applied Biosystems).<br>Cryo movies were collected on a Titan Krios Transmission Electron Microscope (Thermo Fisher Scientific), with the EPU software (v3.8.1)<br>LCMS data were acquired on UPLC-MS/MS (Waters) and Nexera X2 UHPLC system(Shimadzu) coupled with a Sciex 5500 triple quadrupole mass spectrometer

Data analysis	CRISPR screen data analysis and visualization were done using the R programming language (v.4.5.11) and RStudio (v. 2025.05.1+513) with the following packages: tidyverse (v.2.0.0), ggrepel (v.0.9.8), GGally (v.2.4.0), dr4pl (v.2.0.0), ShortRead (Bioconductor v.3.2.4), and Limma. Custom R scripts used to analyze and visualize the data can be found at: https://github.com/Yoonlab-MDA/DCAF11_Nature2026<br><br>Proteome Discoverer 2.4 or 2.5 (Thermo Fisher Scientific) was used for .RAW file processing and controlling peptide and protein level false discovery rates, assembling proteins from peptides, and protein quantification from peptides. Structural data from cryo-EM were processed with cryoSPARC v4.5.3 and 4.6.2, ChimeraX 1.8, ISOLDE 1.8, PHENIX realspacerefine 1.21.2-5419, COOT (0.9.8.92 EL), and SBGrid.<br><br>Flow analysis was done with FlowJo 10.<br><br>All plots were made using Prism 10.

For manuscripts utilizing custom algorithms or software that are central to the research but not yet described in published literature, software must be made available to editors and reviewers. We strongly encourage code deposition in a community repository (e.g. GitHub). See the Nature Portfolio guidelines for submitting code & software for further information.

Data

Policy information about availability of data

All manuscripts must include a data availability statement. This statement should provide the following information, where applicable:

- Accession codes, unique identifiers, or web links for publicly available datasets
- A description of any restrictions on data availability
- For clinical datasets or third party data, please ensure that the statement adheres to our policy

The raw proteomics datasets generated during this study are available at PRIDE accessions: PXD067016, PXD067063, PXD067066, PXD067067, PXD067070, PXD067076. Cryo-EM data are available from the Electron Microscopy Data Bank and the RCSB Protein Data Bank under the accession code numbers: EMD-71847, EMD-71833, EMD-71834 [https://www.ebi.ac.uk/pdbe/entry/emdb/EMD-71847], PDB-9PTU [https://doi.org/10.2210/pdb9PTU/pdb]. PDB-5FQD [https://doi.org/10.2210/pdb5FQD/pdb]. Synthetic procedures are provided in Supplementary Information.

Research involving human participants, their data, or biological material

Policy information about studies with human participants or human data. See also policy information about sex, gender (identity/presentation), and sexual orientation and race, ethnicity and racism.

Reporting on sex and gender	Sex and gender information was not collected in this study
Reporting on race, ethnicity, or other socially relevant groupings	Race and ethnicity information was not collected in this study
Population characteristics	This study included no human participants
Recruitment	This study included no human participants
Ethics oversight	This study included no human participants

Note that full information on the approval of the study protocol must also be provided in the manuscript.

Field-specific reporting

Please select the one below that is the best fit for your research. If you are not sure, read the appropriate sections before making your selection.

☒ Life sciences ☐ Behavioural & social sciences ☐ Ecological, evolutionary & environmental sciences

For a reference copy of the document with all sections, see nature.com/documents/nr-reporting-summary-flat.pdf

Life sciences study design

All studies must disclose on these points even when the disclosure is negative.

Sample size	For proteomic experiments, replicate numbers of initial experiments were based on prior experience. Based on the variation of initial experiments, a sample size of n = 3 or 4 were estimated to be required for detecting reproducible hits.<br><br>For CRISPR screening, number of sgRNAs and replicates were based on prior experience with CRISPR screening.<br><br>For biochemical and cellular assays, the sample sizes were chosen based on previous experience with similar experiments. The sample sizes were sufficient to detect biologically meaningful differences and were consistent with those commonly used in the literature.
Data exclusions	No data was excluded from the analysis

Replication	Number of replicates is described in the figure legends, where applicable
Randomization	No randomization was performed because no risk of systematic selection bias was expected. Covariates were not considered applicable to this study, as all samples were processed under identical experimental conditions and no adjustment for covariates was necessary. The proteomics and functional genomics experiments were performed in a pooled setup – which is intrinsically randomized (i.e. all sgRNAs are expressed in random cells depending on which cell was infected with which sgRNA containing lentivirus).
Blinding	All measurements were performed automatically by devices (flow cytometers, gel scanners, mass spectrometers or NGS devices). NO manual counting or measurements were performed. Therefore, no blinding was required.

Reporting for specific materials, systems and methods

We require information from authors about some types of materials, experimental systems and methods used in many studies. Here, indicate whether each material, system or method listed is relevant to your study. If you are not sure if a list item applies to your research, read the appropriate section before selecting a response.

Materials & experimental systems

n/a	Involved in the study	
☐	☒	Antibodies
☐	☒	Eukaryotic cell lines
☒	☐	Palaeontology and archaeology
☒	☐	Animals and other organisms
☒	☐	Clinical data
☒	☐	Dual use research of concern
☒	☐	Plants

Methods

n/a	Involved in the study	
☒	☐	ChIP-seq
☐	☒	Flow cytometry
☒	☐	MRI-based neuroimaging

Antibodies

Antibodies used

FLAG (CST 14793S), GFP (CST 2555S), β-actin (CST 3700S), Tubulin (Sigma T9026), BRD4 (Bethyl A301-985A-T), DDX18 (GeneTex GTX103392), BRD9 (Bethyl, A303-781A-T), SMARCA2 (CST 8829S), LIMK2 (CST 3845T), WEE1 (CST 4936S), CDK7 (Proteintech 27027-1-AP), cyclin H (Proteintech 67065-1-lg), MNAT1 (Proteintech 11719-1-AP), DCAF11 (Novus Biologicals NBP2-92244), IRDye® 800CW Goat anti-Rabbit IgG Secondary Antibody (LI-COR 926-32211), IRDye® 680LT Goat anti-Rabbit IgG Secondary Antibody (LI-COR 925-68021). All primary antibodies were used at a 1:1,000 dilution, and all secondary antibodies were used at a 1:10,000 dilution.

Validation

All antibodies are commercially available.

anti-FLAG (Cell Signaling Technology, 14793S) was tested by the manufacturer by western blot analysis of extracts from cells expressing DYKDDDDK (FLAG)-tagged proteins.
https://www.cellsignal.com/products/primary-antibodies/dykddddk-tag-d6w5b-rabbit-monoclonal-antibody-binds-to-same-epitope-as-sigma-aldrich-anti-flag-m2-antibody/14793

anti-GFP (Cell Signaling Technology, 2555S) was tested by the manufacturer by western blot analysis of extracts from COS-7 cells mock transfected or transfected with a GFP-tagged fusion protein.
https://www.cellsignal.com/products/primary-antibodies/gfp-antibody/2555

anti-β-actin (Cell Signaling Technology, 3700S) was tested by the manufacturer by western blot analysis of extracts from various cell lines.
https://www.cellsignal.com/products/primary-antibodies/beta-actin-8h10d10-mouse-monoclonal-antibody/3700

anti-α-tubulin (Sigma-Aldrich, T9026) was tested by the manufacturer by western blot analysis of human and chicken fibroblasts.
https://www.sigmaaldrich.com/en/product/sigma/t9026

anti-BRD4 (Bethyl Laboratories, A301-985A-T) was tested by the manufacturer by western blot analysis of endogenous BRD4 in whole-cell lysates from HeLa, HEK293T, MDA-MB-231, Hep-G2, A-549, SW620, and SK-MEL-28
https://www.thermofisher.com/antibody/product/BRD4-Antibody-Polyclonal/A301-985A100

anti-DDX18 (GeneTex, GTX103392) was tested by the manufacturer by western blot analysis of Jurkat, Raji, and NCI H929 lysates
https://www.genetex.com/Product/Detail/DDX18-antibody/GTX103392

anti-BRD9 (Bethyl Laboratories, A303-781A-T) was tested by the manufacturer by western blot analysis of endogenous BRD9 in whole-cell lysates from heLa, K-562, MCF-7, Jurkat, and HEK293T
https://www.thermofisher.com/antibody/product/BRD9-Antibody-Polyclonal/A303-781A

anti-SMARCA2/BRM (Bethyl Laboratories, A301-015A-T) was tested by the manufacturer by western blot analysis of whole cell lysates from Jurkat, HeLa, HEK293T, LNCaP, and Hep-G2 cells prepared using NETN lysis buffer
https://www.thermofisher.com/antibody/product/SMARCA2-BRM-Antibody-Polyclonal/A301-015A-T

anti-LIMK2 (Cell Signaling Technology, 3845T) was tested by the manufacturer by western blot analysis of Colo201 and Jurkat cell

lysates.
https://www.cellsignal.com/products/primary-antibodies/limk2-8c11-rabbit-monoclonal-antibody/3845
anti-WEE1 (Cell Signaling Technology, 4936S) was tested by the manufacturer by western blot analysis of HeLa cell lysates with and without λ-phosphatase
https://www.cellsignal.com/products/primary-antibodies/wee1-antibody/4936

anti-CDK7 (Proteintech, 27027-1-AP) was tested by the manufacturer by western blot analysis of HepG2 and mouse embryo cell lysates.
https://www.ptglab.com/products/CDK7-Antibody-27027-1-AP.htm

anti-Cyclin H (Proteintech, 67065-1-Ig) was tested by the manufacturer by western blot analysis of various cell lysates including HeLa, HEK293T, HepG2, Jurkat, etc.
https://www.ptglab.com/products/Cyclin-H-Antibody-67065-1-Ig.htm

anti-MNAT1 (Proteintech, 11719-1-AP) was tested by the manufacturer by western blot analysis of A375, A431, and HeLa cell lysates.
https://www.ptglab.com/products/MNAT1-Antibody-11719-1-AP.htm

anti-DCAF11 (Novus Biologicals, NBP2-92244) was tested by the manufacturer by western blot analysis of endogenous DCAF11 in mouse liver lysates.
https://www.novusbio.com/products/dcaf11-antibody_nbp2-92244?srsltid=AfmBOopcsbX0zVA7SbLbfdHYJbaOX4Xbp2wzJn5xiwgIhvgJpuirV4Va

IRDye® 800CW Goat anti-Rabbit IgG Secondary Antibody (LI-COR Biosciences, 926-32211) was tested by the manufacturer by dot blot and/or solid-phase adsorption for minimal cross-reactivity with human, mouse, rat, sheep, and chicken serum proteins and was qualified for Western blot and In-Cell Western™ Assay applications.
https://shop.licorbio.com/reagents/irdye-800cw-goat-anti-rabbit-igg-secondary-antibody/

IRDye® 680LT Goat anti-Rabbit IgG Secondary Antibody (LI-COR Biosciences, 925-68021) was tested by the manufacturer by dot blot and solid-phase adsorption assays demonstrating minimal cross-reactivity with human, mouse, rat, sheep, and chicken serum proteins and was validated for Western blot and In-Cell Western™ applications.
https://www.licor.com/bio/reagents/irdye-680lt-goat-anti-rabbit-igg-secondary-antibody

Eukaryotic cell lines

Policy information about cell lines and Sex and Gender in Research

Cell line source(s)

The human HEK293T (CRL-3216) and MOLT4 (CRL-1582) were purchased from ATCC. HEK293T-Cas9, Jurkat cells were provided by the Genetic Perturbation Platform, Broad Institute. K562-Cas9 cells were provided by Zuzana Tothova (Dana-Farber Cancer Institute). Jurkat DCAF11 KO cells were provided by Nathanael Gray (Stanford University). Spodoptera frugiperda (Sf9) insect cells were purchased from Expression Systems (94-001F). Trichoplusia ni High Five insect cells were purchased from Thermo Fisher Scientific (85502)

Authentication

HEK293T, HEK293T-Cas9, and K562-Cas9 cell lines were authenticated by STR profiling. MOLT4, Hi5, and Sf9 cells were not authenticated.

Mycoplasma contamination

Mycoplasma negative (PCR)

Commonly misidentified lines
(See ICLAC register)

None of commonly misidentified lines were used in this study

Plants

Seed stocks

This study did not involve plant seeds

Novel plant genotypes

This study did not involve plants.

Authentication

This study did not involve plants.

Flow Cytometry

Plots

Confirm that:

☒ The axis labels state the marker and fluorochrome used (e.g. CD4-FITC).

☒ The axis scales are clearly visible. Include numbers along axes only for bottom left plot of group (a 'group' is an analysis of identical markers).

☒ All plots are contour plots with outliers or pseudocolor plots.

☒ A numerical value for number of cells or percentage (with statistics) is provided.

Methodology

Sample preparation	Suspension cells were washed with PBS or directly subjected to analysis without fixation
Instrument	MA900 Cell Sorter (Sony) for sorting screens, and BD Symphony Cell Analyzer (BD Biosciences) for flow analysis
Software	BD FACSDiva 8.0 (BD Biosciences), FlowJo 10 (BD Biosciences)
Cell population abundance	Round cells (population with forward and side scatter properties consistent with the alive, non-treated cell line) were usually >50% in most measurements (rarely lower due to drug toxicity), singlets were >90%. For reporter assays, mCherry positive cells >50%
Gating strategy	Cells were first gated for live cells based on forward and side scatter. Single cells were discriminated based on the area vs height of the side scatter. Finally, reporter positive cells were gated based on the mCherry expression.<br>Gating/sorting strategy can also be found in the supplementary figure 2.

☒ Tick this box to confirm that a figure exemplifying the gating strategy is provided in the Supplementary Information.
