# Supplementary material for: DCAF11-dependent molecular glue degrader activated by glutathionylation
Source: Nature. 2026 Aug 5;657(8133):1045–52. doi: 10.1038/s41586-026-10873-1 (PMC13600927; doi:10.1038/s41586-026-10873-1)
Supplement: Supplementary file 3 [file 41586_2026_10873_MOESM4_ESM.pdf]

nature portfolio

Peer Review File

DCAF11-dependent molecular glue degrader activated by glutathionylation

Corresponding Author: Dr Benjamin Ebert

Any redactions in this file are there to maintain patient confidentiality, the confidentiality of unpublished data, or to remove third-party material.

This file contains all reviewer reports in order by version, followed by all author rebuttals in order by version.

Version 0:

Reviewer comments:

Referee #1

(Remarks to the Author)
Approaches for the systematic discovery of molecular glue degraders remain a major bottleneck in the field of targeted protein degradation. In this study, Yoon H. and Wachter F. et al. report a multiplexed, mass spectrometry-based chemical screening approach for the unbiased discovery of molecular glues. By integrating chemoproteomics, genetic screens, and structural studies, they identified M12, a molecular glue that reprograms the E3 ligase substrate adaptor DCAF11 to degrade DDX18, an ATP-dependent RNA helicase. Interestingly, they discovered that M12 functions as a prodrug activated through glutathionylation, which directly enables engagement with DCAF11, promoting DDX18 recruitment, ubiquitination, and subsequent proteasomal degradation. Furthermore, by hijacking DCAF11 via M12, the authors demonstrated that this approach can be extended to degrade a range of proteins through PROTAC-mediated mechanisms. While the findings are intriguing, the data quality is not appropriate in many figures and several questions need to be addressed to improve this manuscript.

1. The cut-off used to identify neosubstrates in the IP–MS experiments shown in Figures 1B, 1D, and Extended Figure 1D appears inconsistent. Can the authors standardize it as applied in Figure 1B? Additionally, could the authors elaborate on the significantly enriched proteins (other than DDX18) identified in Extended Figure 1D and comment on their fate in the global proteomics experiment presented in Figure 2A?
2. Do the authors identify Glutathione S-Transferases (GSTs) as hits in the genome-wide M12 resistance screens? If so, does chemical inhibition or depletion of GSTs (or of specific isoforms, if identified) confer resistance to DDX18 degradation? Additionally, can the authors demonstrate rescue of degradation by treating cells with GSH–M12 (HY-05)?
3. Authors should examine the effect of DDX18 degradation upon depletion of other components of the DCAF11-associated E3 ligase machinery. Evidence confirming DCAF11 knockout is missing throughout the manuscript. Furthermore, the authors should perform a rescue experiment by reintroducing wild-type DCAF11?
4. To further strengthen the biochemical data, the authors should reconstitute all components and perform an in vitro ubiquitination assay for DDX18?
5. Do DCAF11 and DDX18 display intrinsic affinity in the absence of M12 in vitro, and by what magnitude does M12 strengthens the complex formation? Can the authors perform ITC or TR-FRET experiments to rule this out?
6. Does binding of M12 to DCAF11 induce any conformational change in the ligase or its substrate adaptor interface? What proportion of M12 contributes to the neosubstrate footprint between DCAF11 and DDX18? The authors should also discuss the feasibility of employing M12 analogues to modulate neosubstrate specificity.
7. Several xenobiotic compounds are known to undergo glutathionylation in the literature. Can the authors test whether these compounds also engage DCAF11 and exhibit molecular glue activity? Could DCAF11 glues therefore be more widespread than currently recognized?
8. The GSH-binding site of DCAF11 appears highly conserved across evolution, as nicely demonstrated in the manuscript. Can the authors investigate whether the GSH moiety alone, or GSH conjugated to diverse chemical scaffolds, is sufficient to recruit DCAF11 and induce DDX18 degradation? Additionally, does substitution of the reactive chloride (6-Cl) with bromine or other halogens also support GSH–M12 formation in cells?
9. Many cellular proteins undergo glutathionylated under redox stress. Could glutathionylation itself serve as a mark for targeted protein degradation in cells?

10. Most of the molecular glue degraders induce proteasomal degradation of their substrates in short time frame (30mins to 8hrs). however, DDX18 degradation requires high concentration of M12 (10-20 M) and prolonged incubation (~18h, except Figure 2A). Can authors examine and comment on whether these factors contribute to the relatively low cellular activity of M12?
a. What percentage of M12 is converted into GSH-M12 in cells, and is GSH-M12 the only product derived from M12?
b. Since glutathionylation often detoxifies xenobiotic compounds and promote their excretion, does most of the GSH-M12 gets exported out of cells, thus reducing its availability for DCAF11 engagement?
c. Given that glutathione is typically abundant in cells, could free GSH alone occupy the DCAF11 cavity, thereby necessitating higher M12 concentrations and longer exposure times for effective degradation?
11. It is surprising that four independent groups have published studies employing DCAF11; however, none of these works are cited, nor is the identified DCAF11-binding handle compared with previously reported ones. The authors should include a discussion and contextual comparison with these studies.
12. Authors nicely demonstrate dose dependent DDX18 degradation. It would further strengthen the study to include a time-course experiment to better understand the kinetics of DDX18 degradation. Additionally, what is the impact of DDX18 degradation on cellular proliferation?
13. Does DDX18 function as a natural substrate of DCAF11? What is the effect of M12 on endogenous substrates of DCAF11, do the authors observe any change in their degradation or affinity in the MS experiments? Since DCAF11 recognizes phosphorylated degrons (e.g., CENP-A, SLBP), can the authors comment on whether M12 requires or bypasses phosphorylation on DDX18?
14. In Figure 2A, the whole-cell proteomics experiment includes only n = 2 replicates for the M12-treated group versus n = 4 for DMSO. This low replicate number limits statistical confidence. The authors are advised to include at least n = 3 biological replicates per condition.
15. The corresponding text describing Figures 1G, 1H, and 1I, as well as the relevant citations, appears to be missing from the manuscript. The authors should include these details in the main text.

Referee #2

(Remarks to the Author)
In this study, the authors established a new platform for systematic discovery of molecular glues across diverse E3 ligases. By immobilizing multiple E3 ligases on beads and incubating with pooled compounds and cell lysate, this multiplexed immunoprecipitation workflow identifies M12 as a molecular glue that co-opts the E3 ligase DCAF11 to degrade DDX18. Conceivably, this platform could be expanded and applied to other E3 ligases to discover degraders, or other protein-of-interests for the discovery of non-degrader glues.

Another novel finding is that M12 requires the glutathione-S-transferase activity in the cell lysate to become functionalized. Through a series of biochemical dissection, the authors demonstrate that M12 undergoes glutathionylation and the resulting product GSH-M12 fully reconstitutes the glue activity in vitro. This result highlights how drug metabolism can be leveraged for glue discovery.

Finally, structural studies provide mechanistic insights about the interface of the M12-DDX18-DCAF11 complex. Interestingly, GSH-M12 is recognized by a highly conserved pocket on DCAF11, hinting that the physiological function of DCAF11 might be related to GSH regulation. The tight binding of GSH-M12 to DCAF11 also prompts the authors to develop M12-based PROTACs, expanding the current toolbox in targeted protein degradation.

The reviewer finds this study exciting, rigorous, and of immediate interest to a broad audience. It is recommended for publication in Nature, with minor revisions.

1. While the authors discussed that “correlating the cytotoxicity of M12 could lead to advances in the development of antitumor agents with improved selectivity”, the cytotoxicity of M12 has not been shown or investigated in the study. If there is any cytotoxicity, it would be informative to perform a dose-response curve of M12 and test if DDX18 degradation is responsible for M12 cytotoxicity.

2. In Fig. 4e, the authors mutated residues on DCAF11 extensively to validate the ternary interface. Similar mutation studies should also be done on DDX18. Additionally, DDX18 mutants should be evaluated if they confer resistance to M12 induced degradation, as well as cytotoxicity if there is any.

3. It will be informative to measure the binding affinity between DDX18 and DCAF11 with or without GSH-M12, which will help substantiate the glue action in promoting ternary complex formation.

4. Fig 1g-I were not referenced in the main text.

Referee #3

(Remarks to the Author)
I co-reviewed this manuscript with one of the reviewers who provided the listed reports.

Referee #4

(Remarks to the Author)
Yoon et al. describe a new approach for the identification of molecular glue degraders, based on simultaneously multiplexing ligases and compounds in a cellular lysate-based IP-MS screen, aiming to expand the repertoire of E3 ligases that can be employed for the targeted degradation of neo-substrates. The approach is demonstrated using a large compound library screened against a mix of seven E3 ligases, which results in the identification of one compound, M12, that recruits the helicase DDX18 to the E3 ligase DCAF11 for degradation. The authors further show that M12 must undergo glutathionylation by GST in order to act as a molecular glue, and that binding of the glutathionylated M12 to DCAF11 proceeds via a highly conserved binding pocket in DCAF11. These results provide the basis for a novel strategy for targeted protein degradation, using a small molecule prodrug that becomes a molecular glue in vivo by co-opting a specific enzymatic modification, e.g., glutathionylation. The feasibility of this strategy is then demonstrated with the design of other molecular glues, combining glutathionylated M12 with known ligands of a range other proteins of interest, enabling their degradation.

Specific comments
1. That the multiplexed approach greatly increases the potential to discover modes to recruit additional E3 ligases is not really demonstrated. This study reports one very specific - and certainly very interesting - example, but does not provide enough information to claim that the approach could greatly accelerate discovery of E3 ligase binders.

The authors state that the identified molecular glue, M12, would have been unlikely to get identified using other approaches – but this is not due to the multiplexing, but rather the result of using complete cell lysates, containing the enzymes necessary for the glutathione modification.

2. The rationale of multiplexing seven ligases in the screen does not seem to be well supported. Multiplexing often comes with a cost, and I wonder how sensitivity was affected by combining seven E3 ligases? What limited the number of ligases? In this context, the use of only two ligases for the proof-of-principle experiment is surprising - if this was meant as a control experiment, why not use seven ligases, including DCAF15 and CRBN?

Moreover, it seems unclear in what way the initial combined screen of seven E3 ligases conferred any advantages, as opposed to screening the individual E3 ligases, since following hit detection (e.g., of DDX18) one still has to go back and re-run the assay using the compound pool against each ligase individually. Given that identifying the specific compound responsible for mediating the ligase-DDX18 interaction required another series of IP experiments using progressively smaller compound pools, the ligase multiplexing at the beginning of the pipeline does not seem to provide much of an overall benefit, if any, given that there may be sensitivity-related drawbacks of multiplexing, which remain unexplored.

3. Serendipitously, the authors found that DCAF11 may bind specifically to diverse glutathionylated species via a highly conserved GSH binding site. This is a highly significant result that should be highlighted more prominently, also in the abstract. In fact, I think this finding is more significant, in terms of potential impact on drug development and its implications for the biology of E3 ligases, than the rest of the paper, and correspondingly should be explored more thoroughly. For example, does pull-down of DCAF-11 from cell lysate result in enrichment of glutathionylated endogenous metabolites or proteins?

4. It remains unclear whether part or the entirety of the structure of M12 itself has any part in mediating the interactions of the various glutathione conjugates used as DCAF11-based PROTACs with DCAF-11. Is a glutathione moiety alone sufficient? This would be of interest also in the context of the very interesting hypothesis that DCAF11 has evolved to recognize glutathionylated substrates.
Based on the presented results, it seems likely that the structure of M12, beyond its facility to get glutathionylated, is needed only to confer specificity to DDX18. The authors should demonstrate whether, as proposed, glutathionylation alone is sufficient to get DCAF11 binding.
Relatedly, the structures of HY-08, 09, and 10 do not appear to contain M12, as suggested in the text, but rather integrate modified versions lacking the fluoride moiety and a methyl group (unless these are errors in the structures shown in the Figures).

Minor points

1. Methods for the synthesis and spectroscopic characterization for HY-05 and the other M12-derivatives seem to be missing.

2. There seems to be no mention of what DDX18 actually is. While not material to the narrative, the authors should add that this is a DEAD-box RNA helicase that plays a key role in RNA metabolism affecting, ribosome biogenesis, cell cycle progression, and maintenance of stem cell pluripotency.

3. Figure 3: The Figure should show the actual "exact mass" of either GSH-M12, 584.11439, or, more appropriately, of the corresponding m/z as detected in ESI+ mode, m/z 585.12167 (and analogously for M12 itself). the authors should also clarify whether these data were acquired in high-resolution/"exact" mass mode, or at low (unit-mass) resolution.

Version 1:

Reviewer comments:

Referee #1

(Remarks to the Author)
The authors have carefully addressed comments from the first round of review. I would like to acknowledge the considerable effort that has gone into revising the manuscript. The additional experiments, thoughtful rewriting, and improved presentation have strengthened the work and made the main conclusions more compelling. Given that both xenobiotic compounds and endogenous metabolites can engage DCAF11 through glutathionylation, these findings suggest that DCAF11-dependent molecular glue mechanisms may be more widespread than currently recognized. Congratulations to this impressive study and the important discoveries reported here.

I have only a few minor points to consider.

1. The authors demonstrate that DCAF11 binds GSH. It would be interesting to know whether the GSH-binding pocket is playing a regulatory role in DCAF11 ubiquitination activity or contributing primarily to substrate recognition, or both. A brief discussion of this point would be helpful.
2. Considering the high levels of GSH in tumor cells and its ability to bind DCAF11, how feasible would it be to employ DCAF11 as an E3 ligase for oncology therapeutics?

Referee #2

(Remarks to the Author)
The authors have adequately addressed the concerns from this reviewer. Congratulations to the authors again for the exciting story.

Referee #3

(Remarks to the Author)
I co-reviewed this manuscript with one of the reviewers who provided the listed reports.

Referee #4

(Remarks to the Author)
The authors have comprehensively addressed most of my concerns. The added data significantly strengthen the narrative, and the changes to the abstract as well as the added paragraph in the Discussion highlighting the implication of the finding that DCAF11 may bind specifically to diverse glutathionylated species via a highly conserved GSH binding site better highlight broader implications.

Minor remaining concerns:

- I still find the focus on multiplexing in the initial part of the Results distracting. The authors seem to agree that any advantages of multiplexing may be minor and diminishing as MS costs come down, so there’s really no reason to elaborate much on this. Further, the critical use of cell lysate in the assay is not highlighted, which, while not unique, distinguishes the screen in this paper from others.

- Figure 3f does not show the synthesis of GSH-M12, only its structure.

- Assay concentrations of M12 and GSH-M12 are missing in many Figures, e.g., AR Figure 4.

Open Access This Peer Review File is licensed under a Creative Commons Attribution 4.0 International License, which permits use, sharing, adaptation, distribution and reproduction in any medium or format, as long as you give appropriate credit to the original author(s) and the source, provide a link to the Creative Commons license, and indicate if changes were made.
In cases where reviewers are anonymous, credit should be given to 'Anonymous Referee' and the source.
The images or other third party material in this Peer Review File are included in the article's Creative Commons license, unless indicated otherwise in a credit line to the material. If material is not included in the article's Creative Commons license and your intended use is not permitted by statutory regulation or exceeds the permitted use, you will need to obtain permission directly from the copyright holder.
To view a copy of this license, visit https://creativecommons.org/licenses/by/4.0/

Reviewer comments:

Referee #1 (Remarks to the Author):

Approaches for the systematic discovery of molecular glue degraders remain a major bottleneck in the field of targeted protein degradation. In this study, Yoon H. and Wachter F. et al. report a multiplexed, mass spectrometry-based chemical screening approach for the unbiased discovery of molecular glues. By integrating chemoproteomics, genetic screens, and structural studies, they identified M12, a molecular glue that reprograms the E3 ligase substrate adaptor DCAF11 to degrade DDX18, an ATP-dependent RNA helicase. Interestingly, they discovered that M12 functions as a prodrug activated through glutathionylation, which directly enables engagement with DCAF11, promoting DDX18 recruitment, ubiquitination, and subsequent proteasomal degradation. Furthermore, by hijacking DCAF11 via M12, the authors demonstrated that this approach can be extended to degrade a range of proteins through PROTAC-mediated mechanisms. While the findings are intriguing, the data quality is not appropriate in many figures and several questions need to be addressed to improve this manuscript.

Author Response: We thank the reviewer for carefully reviewing our manuscript, and we appreciate the thoughtful comments and insightful questions, which we believe have substantially improved our manuscript. During the revision, we conducted extensive additional studies demonstrating that glutathione directly binds to DCAF11. These findings allowed us to more clearly define the role of glutathione in DCAF11-dependent substrate recognition and degradation. The following experiments and figures have been added to the revised manuscript in response to the reviewers' questions:

- Whole-proteome quantification following M12 treatment (Fig. 2a and Extended Data Fig. 2a)
- Time-course of DDX18 degradation by M12 (Extended Data Fig. 2d)
- The effect of DDX18 degradation on cell viability (Extended Data Fig. 2e)
- The effect of DCAF11 KO on M12-induced DDX18 degradation (Fig. 2g and Extended Data Fig. 2h-j)
- Conversion of M12 to GSH-M12 in cell (Extended Data Fig. 3d)
- Binding of GFP-DDX18 to DCAF11 by TR FRET in the presence of M12/lysate and GSH-M12 (TR-FRET) (Extended Data Fig. 3h)
- *In vitro* ubiquitination of DDX18 by DCAF11 in the presence of GSH-M12 (Extended Data Fig. 3i)
- DDX18 mutants degradation by M12 (Extended Data Fig. 4g)
- Binding of GSH to DCAF11 (TR-FRET) (Extended Data Fig. 5a,b)
- Inhibition of M12 mediated DCAF11/DDX18 complex formation by GSH (TR-FRET and co-IP) (Extended Data Fig. 5c-e)
- Binding of xenobiotic compounds to DCAF11 (Extended Data Fig. 5f,g)
- Halogen-substituted analogs (Br, F, I) bind to DCAF11 in accordance with their expected GSH/GST-mediated nucleophilic reactivity (Extended Data Fig. 6c-e)

1. The cut-off used to identify neosubstrates in the IP–MS experiments shown in Figures 1B, 1D, and Extended Figure 1D appears inconsistent. Can the authors standardize it as applied in Figure 1B? Additionally, could the authors elaborate on the significantly enriched proteins (other than DDX18) identified in Extended Figure 1D and comment on their fate in the global proteomics experiment presented in Figure 2A?

Author Response: We thank the reviewer for pointing this out. We now apply the same cutoff across all IP-MS experiments (Hits: LogFC > 3, *p*-value <0.001) in the manuscript and have updated the revised manuscript with new plots (**Fig 1d**; **Extended Data Fig. 1a, d**). The only potential other candidate enriched in addition to DDX18 in our IP-MS is HMGB1, but it did not find any decreased expression of HMGB1 in our global proteomics (Hits: Fold-change > 2, *p*-value <0.001) (**Author Response Fig. 1b,c**).

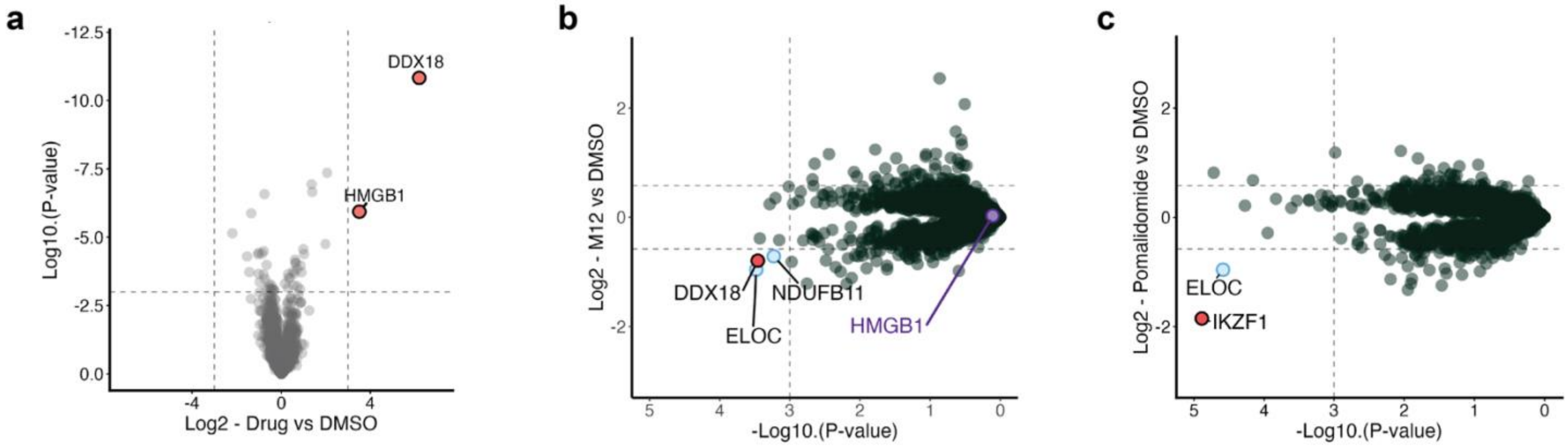

Author Response Figure 1. a, IP-MS: DCAF11 IP followed by mass spectrometry using Molt4 cell lysates, treated with either DMSO (*n* = 4) or 10 µM M12 (*n* = 4). **b.** Whole-proteome quantification of Jurkat cells treated with 20 µM M12 (*n* = 3) or DMSO (*n* = 3) for 6h. **c.** Whole-proteome quantification of Jurkat cells treated with 1 µM pomalidomide (*n* = 3) or DMSO (*n* = 3) for 6h. ELOC is a common contaminant in both proteomics experiments.

2. Do the authors identify Glutathione S-Transferases (GSTs) as hits in the genome-wide M12 resistance screens? If so, does chemical inhibition or depletion of GSTs (or of specific isoforms, if identified) confer resistance to DDX18 degradation? Additionally, can the authors demonstrate rescue of degradation by treating cells with GSH–M12 (HY-05)?

Author Response: We thank the reviewer for these insightful questions. Likely due to multiple isoforms and functional redundancy, GSTs do not score in our genome-wide screens to identify sgRNAs that confer resistance to M12-induced DDX18 degradation. Available inhibitors do not comprehensively block all GST isoenzymes, limiting their impact on degradation. The impact of genetic inactivation of GSTs in our genome-wide screen are shown in the vocano plot in which we annotated all identified GSTs (**Author Response Fig. 2a**). There are five major classes of GST isoforms (A, M, P, T, and Z), which also have subfamilies (1,2,3…), thus comprising over 20 GST enzymes in the human genome[1], many of which share highly similar enzymatic functions. Therefore, we speculate that multiple GST enzymes may be able to act redundantly to mediate the glutathionylation of M12. Consistent with this, we tested several GST enzyme inhibitors (**Author Response Fig. 2b**), but none showed a rescue effect upon treatment.

Additionally, we observed efficient DDX18-DCAF11 complex formation both in mammalian cells and insect cells, suggesting GSTs from both species are capable of glutathionylating M12. In our *in vitro* conversion of M12 to GSH-M12 (Fig. 3h and Extended Data Fig 3d), we used recombinant *S.japonicum* GST protein (Uniprot ID# P08515) and observed efficient conversion of M12 to GSH-M12, which further demonstrates that diverse GST isoforms have capacity for the conversion of GSH-M12. Of note, Synthetic GSH-M12 (HY-05) is unable to penetrate the cell membrane due to its highly-charged nature (**Author Response Fig. 2c**).

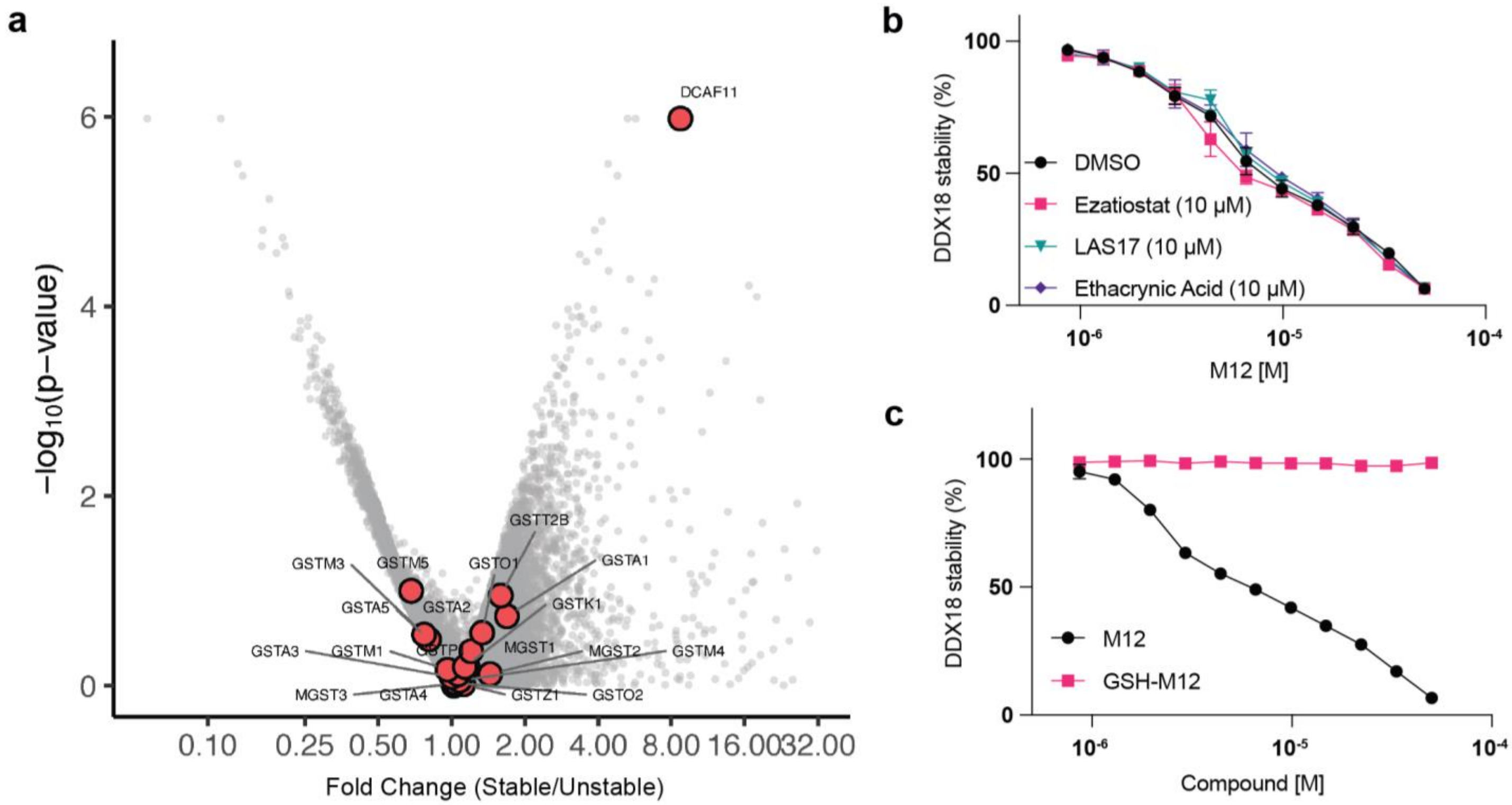

Author Response Figure 2. a. The same volcano plot of a genome-wide CRISPR screen for M12-mediated DDX18 degradation as shown in **Extended Data Fig. 2f,g** with all annotated GSTs marked on the plot. **b**. Flow cytometry analysis of DDX18-eGFP reporters in $K562_{cas9}$ cells with M12 + DMSO or GST inhibitors (Ethacrynic acid: Pan-GSTs inhibitor; LAS17&Ezatiostat: GSTP1 inhibitor). GST inhibitors were treated 1h before M12 treatment, and the cells were incubated for 18h after M12 treatment. DDX18 stability was calculated as the eGFP/mCherry fluorescence ratio. **c**. Flow cytometry analysis of DDX18-eGFP reporters in $K562_{cas9}$ cells with M12 or GSH-M12 for 18 h. DDX18 stability was calculated as the eGFP/mCherry fluorescence ratio.

3. Authors should examine the effect of DDX18 degradation upon depletion of other components of the DCAF11-associated E3 ligase machinery. Evidence confirming DCAF11 knockout is missing throughout the manuscript. Furthermore, the authors should perform a rescue experiment by reintroducing wild-type DCAF11?

Author Response: We thank the reviewer for pointing out the missing controls confirming DCAF11 knockout. We have now updated **Fig. 2g** to include the immunoblot confirming DCAF11 knockout (also **Author Response Fig. 3a**). In addition, we performed a rescue experiment by reintroducing wild-type or mutant DCAF11 as suggested by the reviewer (**Author Response Fig. 3b**).

We also included a UPS-focused CRISPR degradation screen in DDX18-eGFP reporter cells treated with DMSO (**Author Response Fig. 3c; Extended Data Fig. 2h**). Normalized read count comparisions between M12 vs DMSO confirmed that sgRNAs targeting *DCAF11* and *UBE2G1* are enriched only in the M12-treated, eGFP-stable population **(Author Response Fig. 3d; Extended Data Fig. 2i**). Additionally, in our *in vitro* ubiquitination assay, reconstitution of $CRL4^{DCAF11}$ E3 ligase complex machinery is sufficient to drive ubiquitination of DDX18 (**Author Response Fig. 4**).

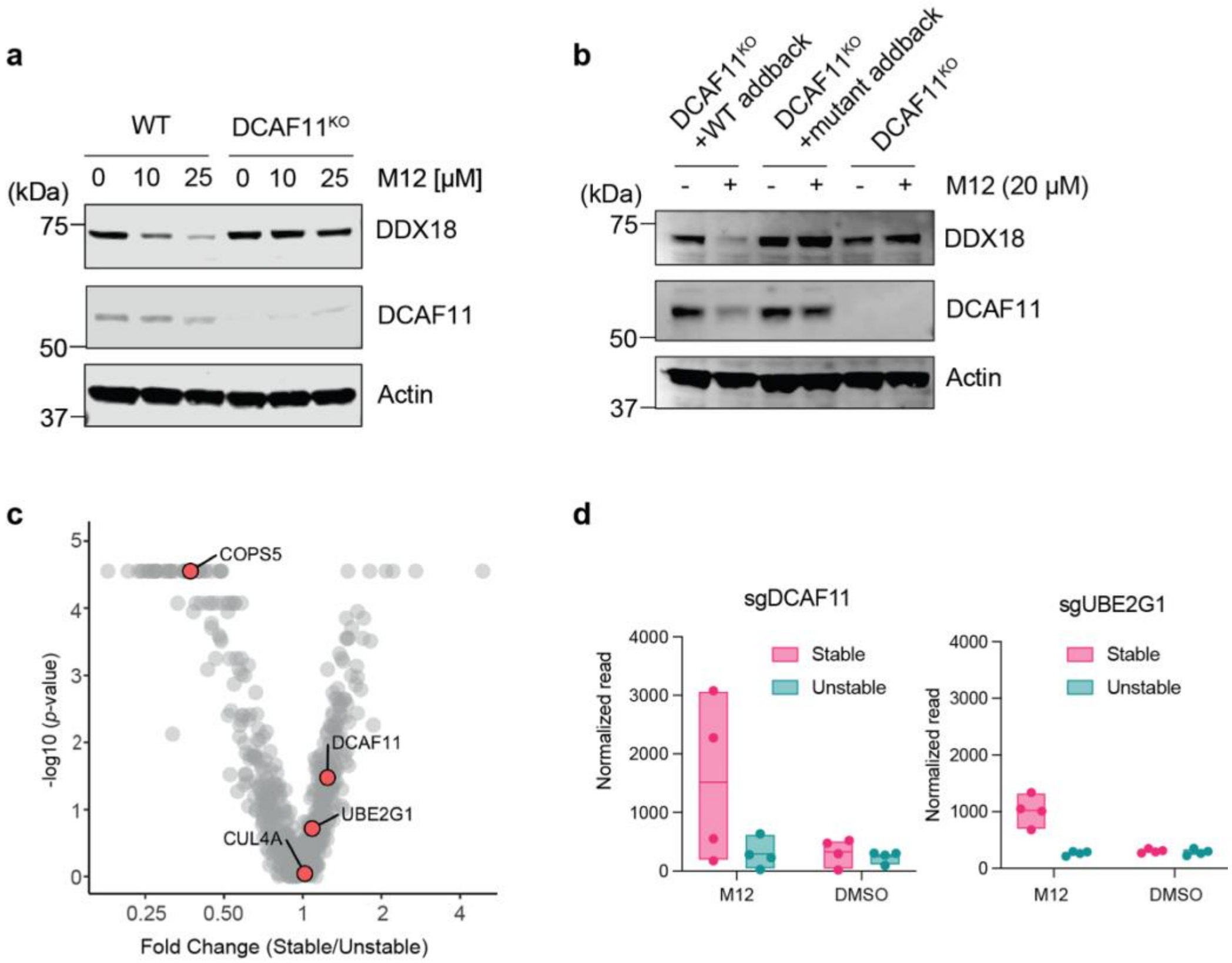

Auther Response Figure 3. a. New **Fig. 2g** demonstrating rescue of DDX18 degradation upon CRISPR mediated knock-out of DCAF11. Immunoblots for DCAF11 are now included **b**. Immunoblots from the lysate of $K562_{Cas9}$ cells with DCAF11 KO, DCAF11 KO + WT addback, and DCAF11 KO + GSH-binding defect mutant (D309A N311A D328A), treated with DMSO or 20 μM M12 for 6 hours. **c**. UPS-focused CRISPR degradation screen for DDX18-eGFP reporter in $K562_{cas9}$ cells treated with DMSO for 18 h (*n* = 3). **d**. UPS-focused CRISPR degradation screen for DDX18-eGFP reporter: Normalized read counts (M12 or DMSO) in each sorted gate for 4 sgRNAs targeting *DCAF11* and *UBE2G1*.

4. To further strengthen the biochemical data, the authors should reconstitute all components and perform an in vitro ubiquitination assay for DDX18?

Author Response: We thank the reviewer for this suggestion. We performed an *in vitro* ubiquitination assay showing robust polyubiquitination of DDX18 by DCAF11 only in the presence of GSH-M12 in a time dependent manner (**Author Response Fig**. **4**). This data has now also been included in the revised manuscript (**Extended Data Fig**. **3i**).

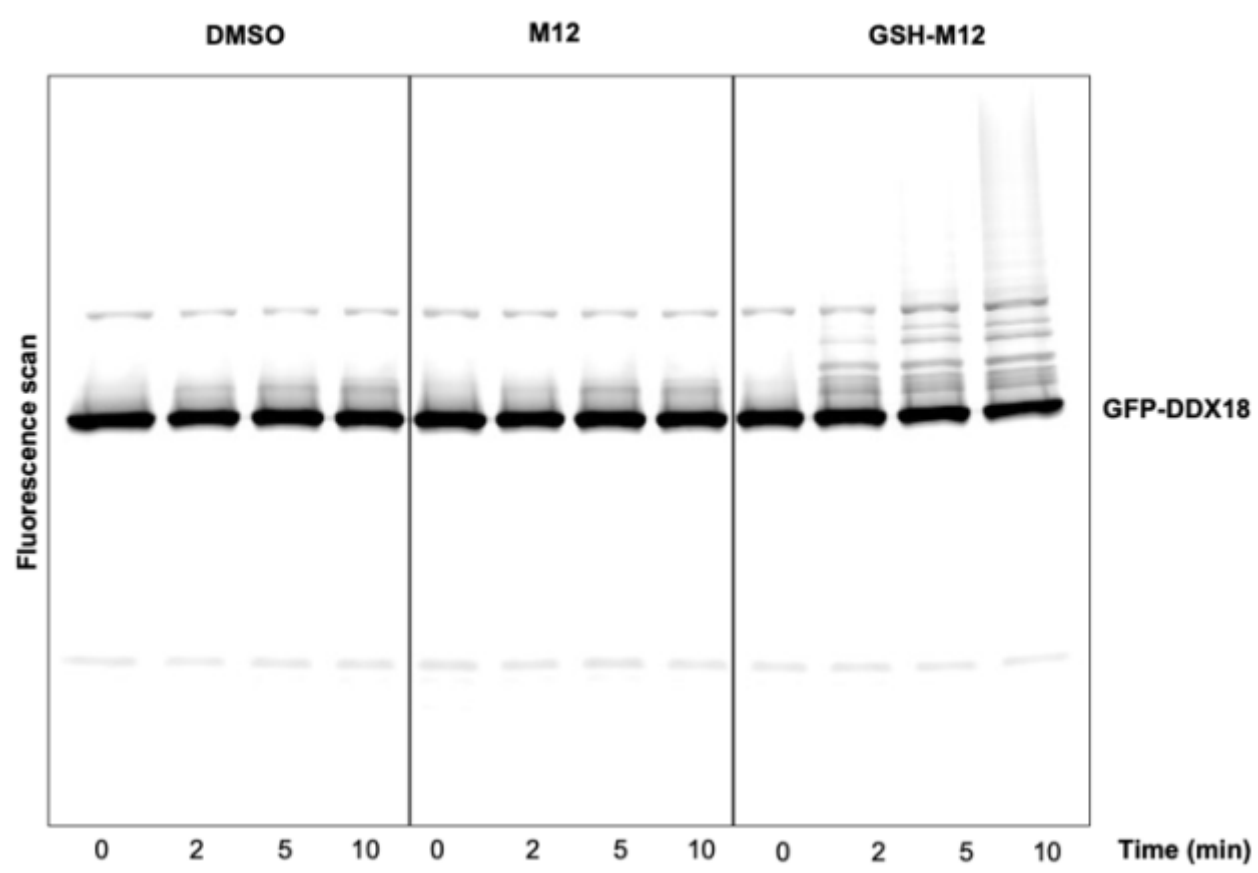

Auther Response Figure 4. *In vitro* ubiquitination assay. 500 nM neddylated CUL4-RBX1 was incubated with 700 nM DCAF11-DDB1-DDA1 and 1 μM GFP-DDX18 with buffer, M12, or GSH-M12 on ice for 20 minutes. Reaction was performed in 25 mM HEPES, 100 mM NaCl, 10 mM $MgCl_2$, 5 mM ATP, with 2 μM UBE2D, 2 μM UBE2G1, 0.2 μM UBA1, and was initiated by adding 60 μM ubiquitin. The gel was visualized on an Amersham Typhoon imager. Shown here 1 out of 2 replicates.

5. Do DCAF11 and DDX18 display intrinsic affinity in the absence of M12 in vitro, and by what magnitude does M12 strengthens the complex formation? Can the authors perform ITC or TR-FRET experiments to rule this out?

Author Response: We performed TR-FRET binding assays in the presence or absence of GSH-M12 and did not observe detectable binding between DCAF11 and DDX18 in the absence of GSH-M12 and at the concerntrations tested (**Author Response Fig. 5).** This data has now also been included in the revised manuscript (**Extended Data Fig. 3h**).

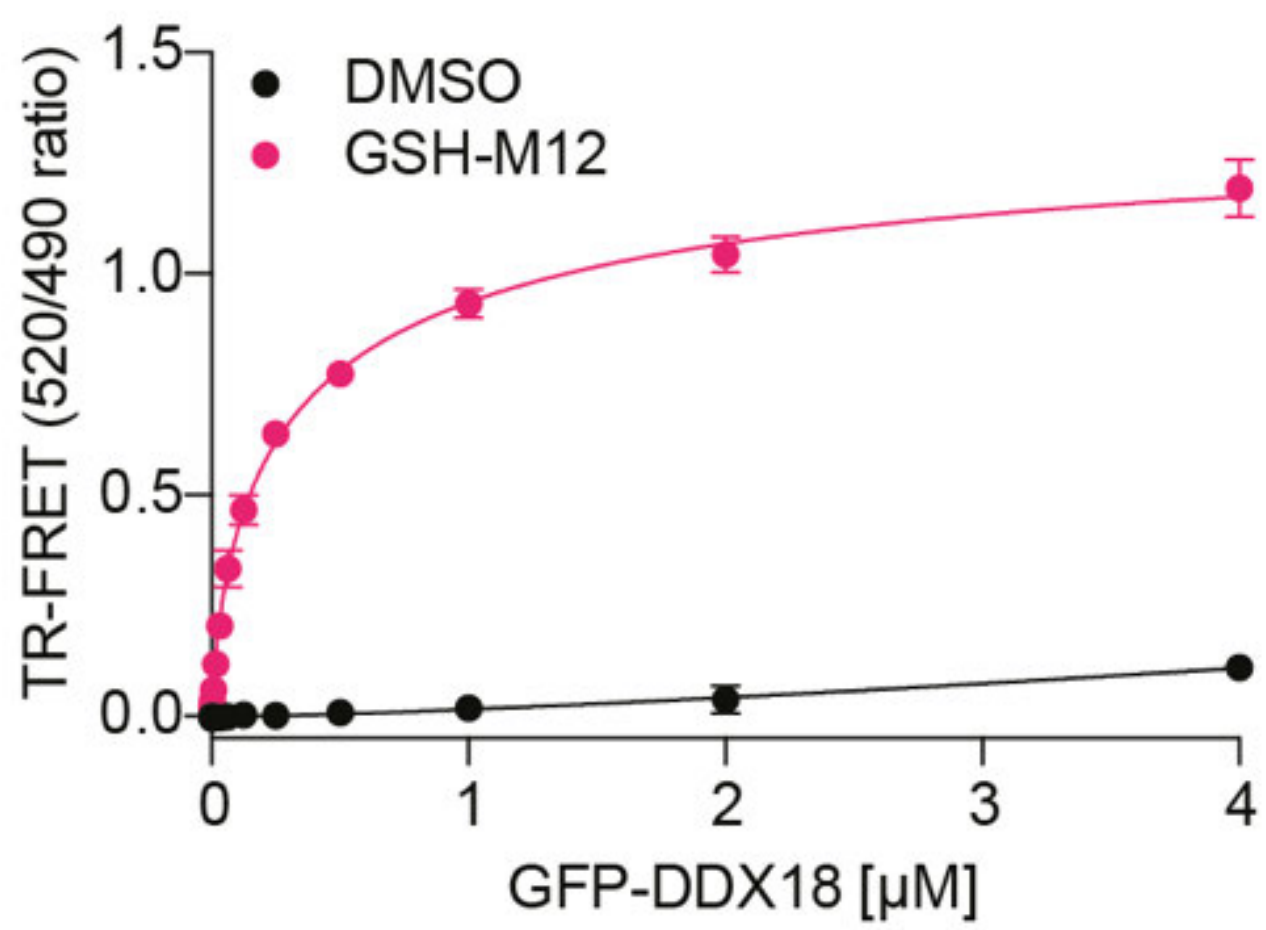

Author Response Figure 5. TR-FRET assay: Titration of DDX18 to terbium-labeled 100nM DCAF11 +/- 200nM GSH M12 (HY-05).

6. Does binding of M12 to DCAF11 induce any conformational change in the ligase or its substrate adaptor interface? What proportion of M12 contributes to the neosubstrate footprint between DCAF11 and DDX18? The authors should also discuss the feasibility of employing M12 analogues to modulate neosubstrate specificity.

Author Response: [redacted]

GSH-M12 occupies 240Å^2 on DDX18 compared to 507Å^2 for DCAF11, which is consistent with clearly defined interactions such as a hydrogen bond with R408 in DCAF11 via the nitrogen atom of its pyridine ring, and this in turn stabilizes a polar interaction network involving R408-D406-W409 in DCAF11. This network is likely to position DCAF11 W409 indole side chain, creating space that accommodates closer positioning of the DDX18 a-helix. Additionally, the carbonyl group of M12 forms a hydrogen bond with V406 of DDX18, and E550 of DDX18 forms a hydrogen bond with R408 of DCAF11 (**Fig. 4b**). The 5-F group of M12 appears critical for DDX18 recruitment. Analogs without the 5-F but bearing alternative halogen substitutions at the 6-position can still bind to DCAF11, but do not mediate DDX18 recruitment. (**Author Response Fig. 6b-d**). This data has now also been included in the revised manuscript (**Extended Data Fig. 6c-e**) and discuccsed in line 330 – *"Analogs without the 5-F but bearing alternative halogen substitutions at the 6-position can still bind to DCAF11, but do not mediate DDX18 recruitment"*

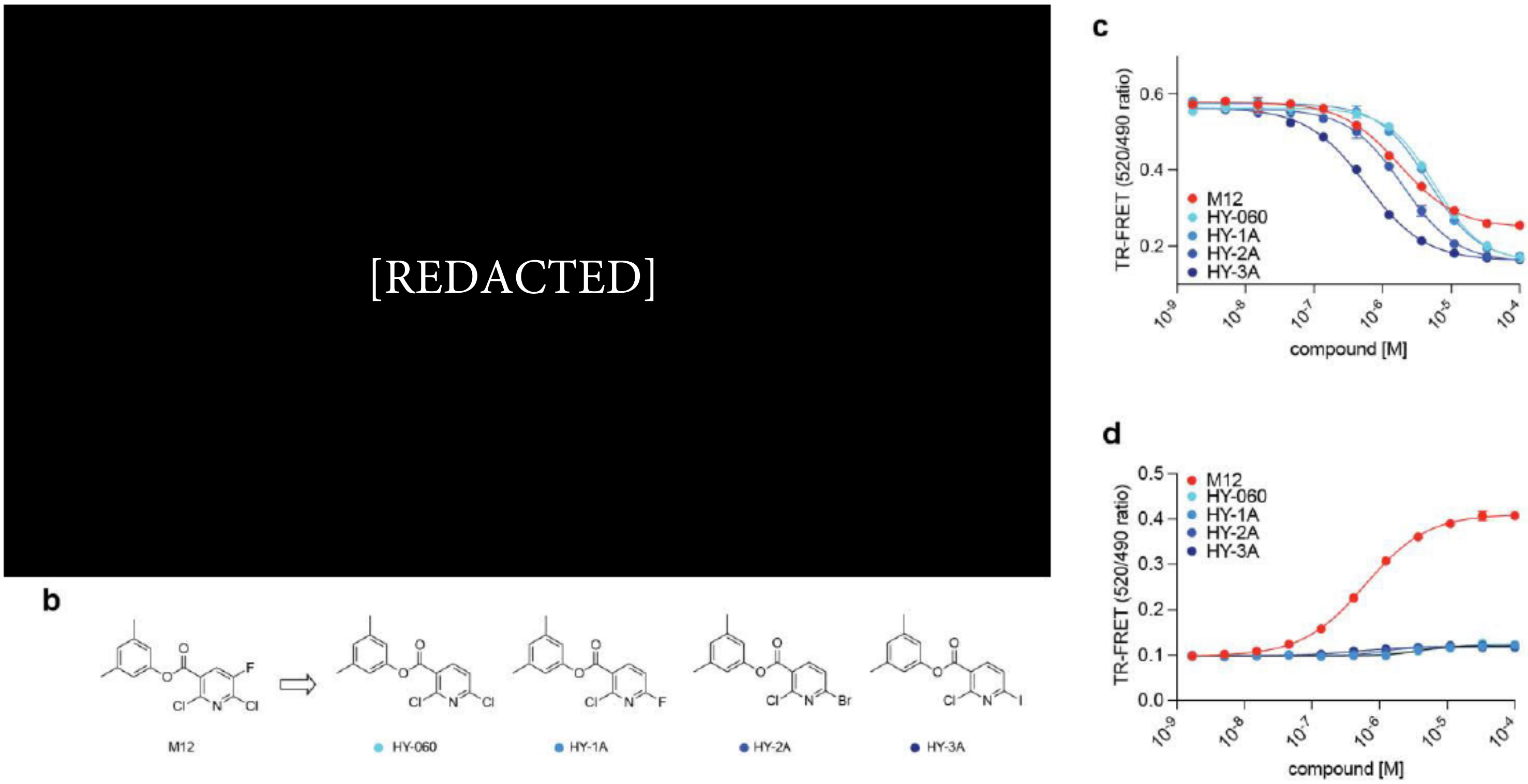

Auther Response Figure 6. [redacted] **b**. Chemical structure of M12 analogs containing other halogen atoms. **c**. TR-FRET: Pre-assembled 390 nM BODIPY-M12 (HY-06) and 100nM terbium-labeled DCAF11 were treated with M12 or its analogs in indicated concentrations. **d**. TR-FRET: Pre-assembled 300 nM GFP-DDX18 and 100 nM terbium-labeled DCAF11 were treated with M12 or its analogs.

7. Several xenobiotic compounds are known to undergo glutathionylation in the literature. Can the authors test whether these compounds also engage DCAF11 and exhibit molecular glue activity? Could DCAF11 glues therefore be more widespread than currently recognized?

Author Response: This is an excellent question. We performed experiments with multiple xenobiotic compounds and confirmed the reviewer's hypothesis that these would likely bind. We tested several xenobiotics known to be glutathionylated, including JS-K[2], CDNB[3], and azathioprine[3,4] (**Author Response Fig. 7a**), for binding to DCAF11 in the setting of lysate to allow glutathionylation as previously reported. JS-K, CDNB, and its glutathionylated active form L-glutamyl-S-(2,4-dinitrophenyl)-L-cyteinyl glycine displace BODIPY-M12 (HY-06) from DCAF11 in a dose-dependent manner, suggesting their glutathionylated species bind to DCAF11 and compete with glutathionylated BODIPY-M12 for DCAF11 binding at the same pocket (**Author Response Fig. 7b**).

We tested whether those DCAF11-binding xenobiotics exihibit molecular glue activity by IP-MS. All three xenobiotics with DCAF11 binding affinity showed enrichement of GSK3A/B and ZBTB18 (**Author Response Fig. 7c,d**), but none of them induced degradation of the recruited proteins (**Author Response Fig. 7e**). Given these inconclusive results, we decided against including it in the manuscript and further exploring the possibility of xenobiotic compounds acting as DCAF11 glues.

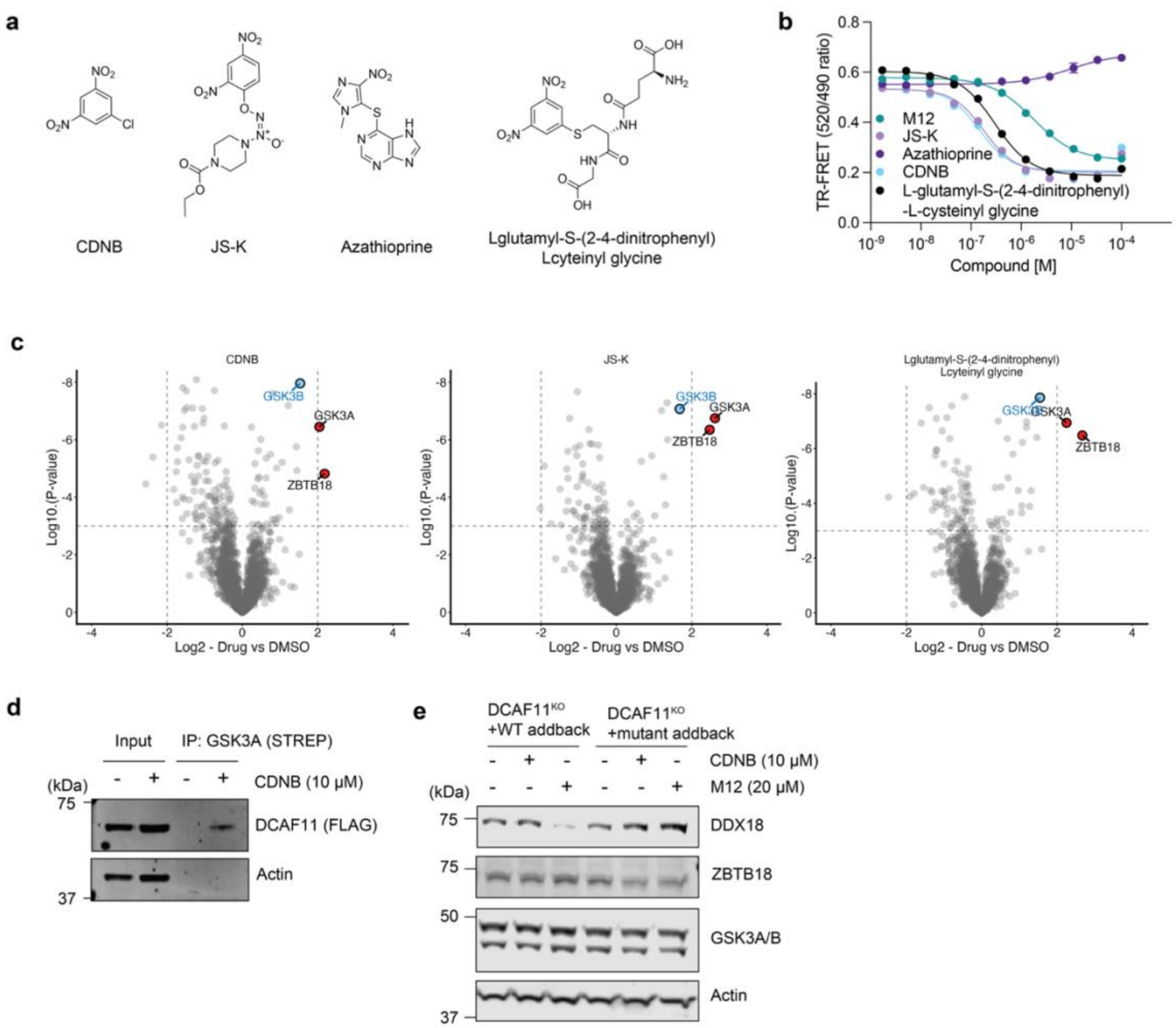

Author Response Figure 7. **a**. Chemical structure of the tested xenobiotics. **b**. TR-FRET assay: Pre-assmbled 390 nM BODIPY-M12 (HY-06) and 100 nM terbium-labeled DCAF11 were treated with M12 or xenobiotic compounds in lysate buffer. **c**. IP-MS: DCAF11 IP followed by mass spectrometry with Molt4 cellular lysate, treated with either DMSO (*n* = 4) or 10 μM xenobiotics (*n* = 4). **d**. DCAF11 IP followed by western blots in the presence of DMSO or 10 μM CDNB from 293T cells transfected with FLAG-DCAF11 and STREP-GSK3A constructs. **e**. Immunoblots of DDX18, ZBTB18, and GSK3A/B in DCAF11 KO K562 cells, or wild-type (WT) addback, DCAF11 mutant (D309A/N311A/D328A) addback. Cells were treated with DMSO, CDNB (10 μM) or M12 (20 μM) for 18 h, and DDX18 levels were assessed by immunoblot.

8. The GSH-binding site of DCAF11 appears highly conserved across evolution, as nicely demonstrated in the manuscript. Can the authors investigate whether the GSH moiety alone, or GSH conjugated to diverse chemical scaffolds, is sufficient to recruit DCAF11 and induce DDX18 degradation?

Author Response: To investigate whether GSH alone directly binds to DCAF11, we performed experiments, all of which demonstrate that GSH alone binds to DCAF11 (**Author Response Fig. 8a-e**). This data has been included in the revised manuscript (**Extended Data Fig. 5**).

1) We synthesized a BODIPY-labeled GSH probe and confirmed that the probe binds to DCAF11.
2) We also show that an increasing concentration of reduced GSH inhibitis M12-mediated DDX18 recruitment in both TR-FRET and IP-western blot.

Additionally, does substitution of the reactive chloride (6-Cl) with bromine or other halogens also support GSH–M12 formation in cells?

Author Response: Halogen-substituted analogs of M12 (Br, F, I) bind to DCAF11 in accordance with their expected GSH/GST-mediated nucleophilic reactivity (**Author Response Fig. 8f,g; Extended Data Fig. 6c,d**).

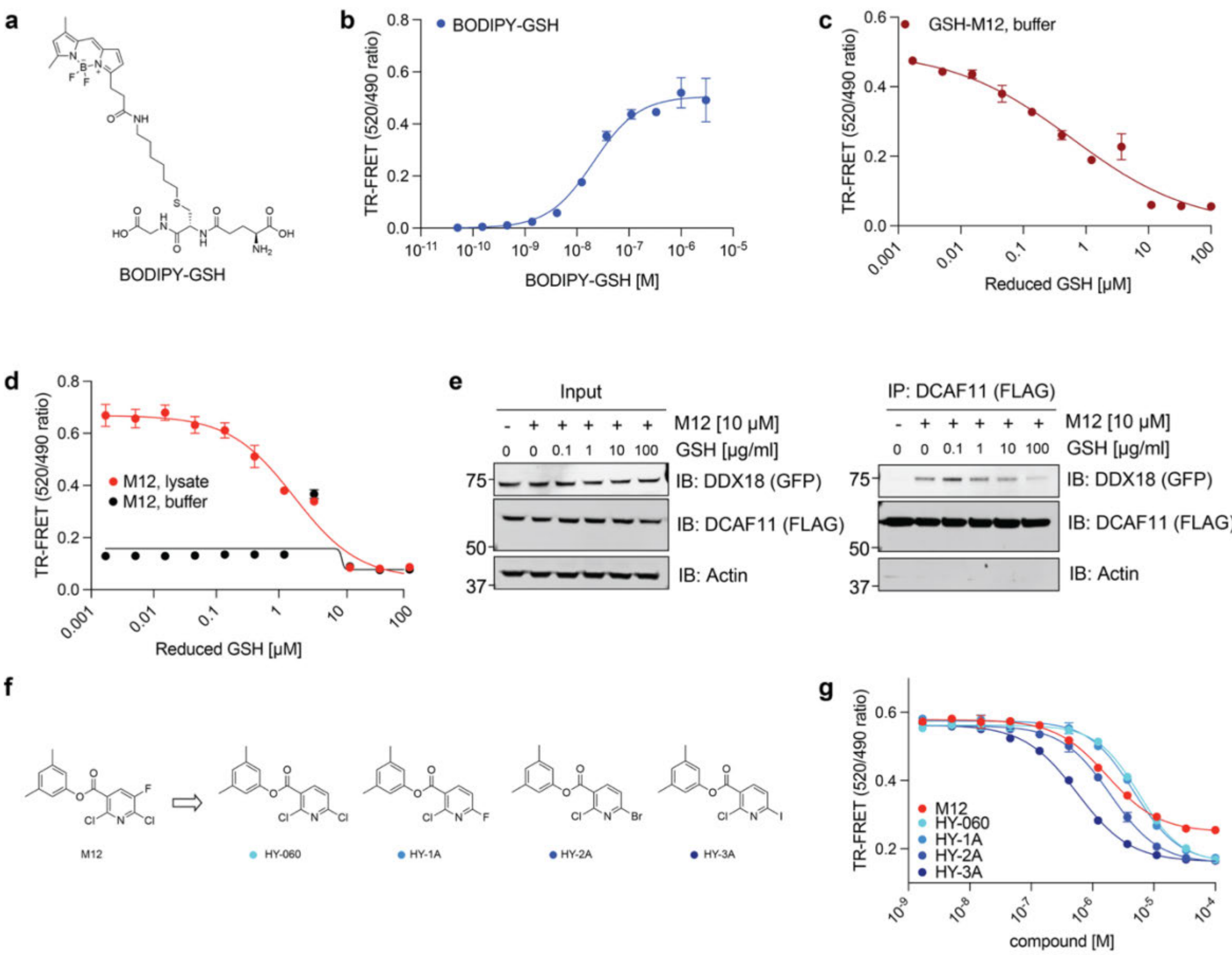

Author Response Figure 8. a. Chemical structure of BODIPY-GSH. **b**. TR-FRET: Titration of BODIPY-GSH to 100nM teribium-labeled DCAF11, demonstrating its direct binding to DCAF11. **c**. TR-FRET: Pre-assembled complex of 300 nM GFP-DDX18 and 100 nM terbium-labeled DCAF11 formed by 50 nM GSH-M12 in regular buffer were treated with an increasing concentration of reduced GSH. **d**. TR-FRET: Pre-assembled complex of 300 nM GFP-DDX18 and 100 nM terbium-labeled DCAF11 by 200 nM M12 in lysate or buffer conditions were treated with an increasing concentration of reduced GSH. **e**. Immunoblot of DCAF11 IP +/- M12, with increasing concentration of reduced GSH, using cell lysate expressing DDX18-eGFP. **f**. Chemical structure of M12 analogs

containing other halogen atoms. **g**. TR-FRET: Pre-assembled 390 nM BODIPY-M12 and 100 nM terbium-labeled DCAF11 in lysate buffer were treated with M12 or its analogs.

9. Many cellular proteins undergo glutathionylated under redox stress. Could glutathionylation itself serve as a mark for targeted protein degradation in cells?

Author Response: We agree with the reviewer that this is an intriguing and impactful question that we have considered deeply. Protein S-glutathionylation is a reversible post-translational modification where glutathione binds to protein's cysteine residues, particularly under redox stress, and the identified mechanism could serve as a quality control program for damaged proteins[5].

[redacted] Moreover, we have demonstrated in biochemical studies that glutathione itself directly binds to DCAF11 (**Author Response Fig. 8a-e**).

[redacted]

10. Most of the molecular glue degraders induce proteasomal degradation of their substrates in short time frame (30mins to 8hrs). however, DDX18 degradation requires high concentration of M12 (10-20 μM) and prolonged incubation (~18h, except Figure 2A). Can authors examine and comment on whether these factors contribute to the relatively low cellular activity of M12?

Author Response: We thank the reviewer for this insightful question. M12-mediated DDX18 degradation works through a unique, multi-step mechanism, which requires glutathionylation prior to binding to DCAF11, recruitment of DDX18, ubiquitylation and subsequent proteasomal degradation.

The relatively low cellular activity can be explained by several factors. First, the cellular conversion of M12 to GSH-M12 is relatively low (~10-15%, **Author Response Fig. 9a**), and the compound is also rapidly hydrolzyed due to its labile ester bond (**Author Response Fig. 9b,c**). Second, we have shown that DCAF11 can bind GSH alone or glutathionylated species (**Author Response Fig. 8a-e**). Given the relatively high intracellular concentration of GSH-containing molecules, it is likely that GSH-M12 competes with several endogenous molecules containing GSH moiety for DCAF11 binding. These factors likely contribute to the low cellular activity compared to the robust *in vitro* activity for DDX18 recruitment. Please also find our point-by-point responses to the specific questions below.

a. What percentage of M12 is converted into GSH-M12 in cells, and is GSH-M12 the only product derived from M12?

Author Response: We quantified intracellular GSH-M12 levels and its concentration in the media (to assess potential export, as suggested by the reviewer) using LC-HRMS, and used these measurements to calculate the total conversion rate over time. Based on our measurement, around 15% of M12 treated was converted to GSH-M12, which explains the relatively low cellular activity of M12 (**Author Response Fig. 9a**).

Additionally, we analyzed M12-derived products in the same samples (1h time point) using High-Resolution MS (HRMS). We identified GSH-M12 and a hydrolyzed form of GSH-M12. Hydrolyzed GSH-M12 as detectable products of M12 (**Author Response Fig. 9b,c**). Hydrolyzed M12 accounted for 16.7% of the detected M12-derived species (83.3% is GSH-M12), indicating that the ester bond is labile to hydrolysis. M12 was poorly ionized in HRMS thus was not included in the percentage calculation.

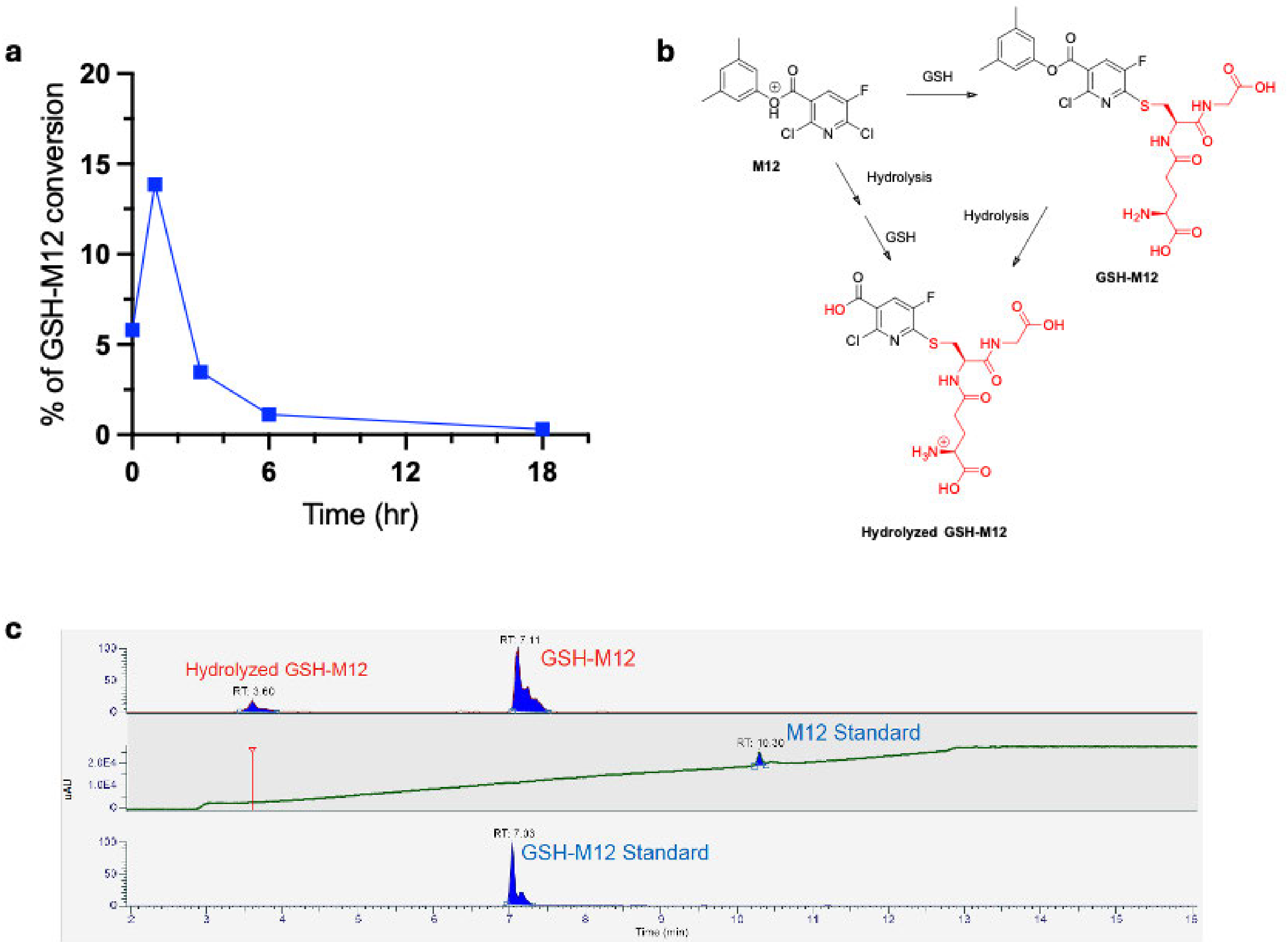

Author Response Figure 9. a. GSH-M12 converstion rate. K562 cells were treated with M12 (10 μM), and GSH-M12 formation was quantified by measuring its concentration in both media and cells. The conversion rate was calculated based on the total amount of the M12 treated (10 μM in 1 mL). b. Proposed metabolic pathways of M12. c.(Top) LC-HRMS chromatogram of cell lysate samples (1h), showing two major species: GSH-M12 and hydrolyzed GSH-M12. (Middle) LC-HRMS chromatogram of M12 standard. (Bottom) LC-HRMS chromatogram of a synthetic GSH-M12 standard (HY-05).

b. Since glutathionylation often detoxifies xenobiotic compounds and promote their excretion, does most of the GSH-M12 gets exported out of cells, thus reducing its availability for DCAF11 engagement?

Author Response: We detected a substantial portion of GSH-M12 in the cell media (total media volume 1 mL), with a rapid decrease in intraceullar GSH-M12 levels within 6 hours. In addition to the factors discussed above, this likely contributes to the low cellular activity of M12 in promoting DDX18 degradation.

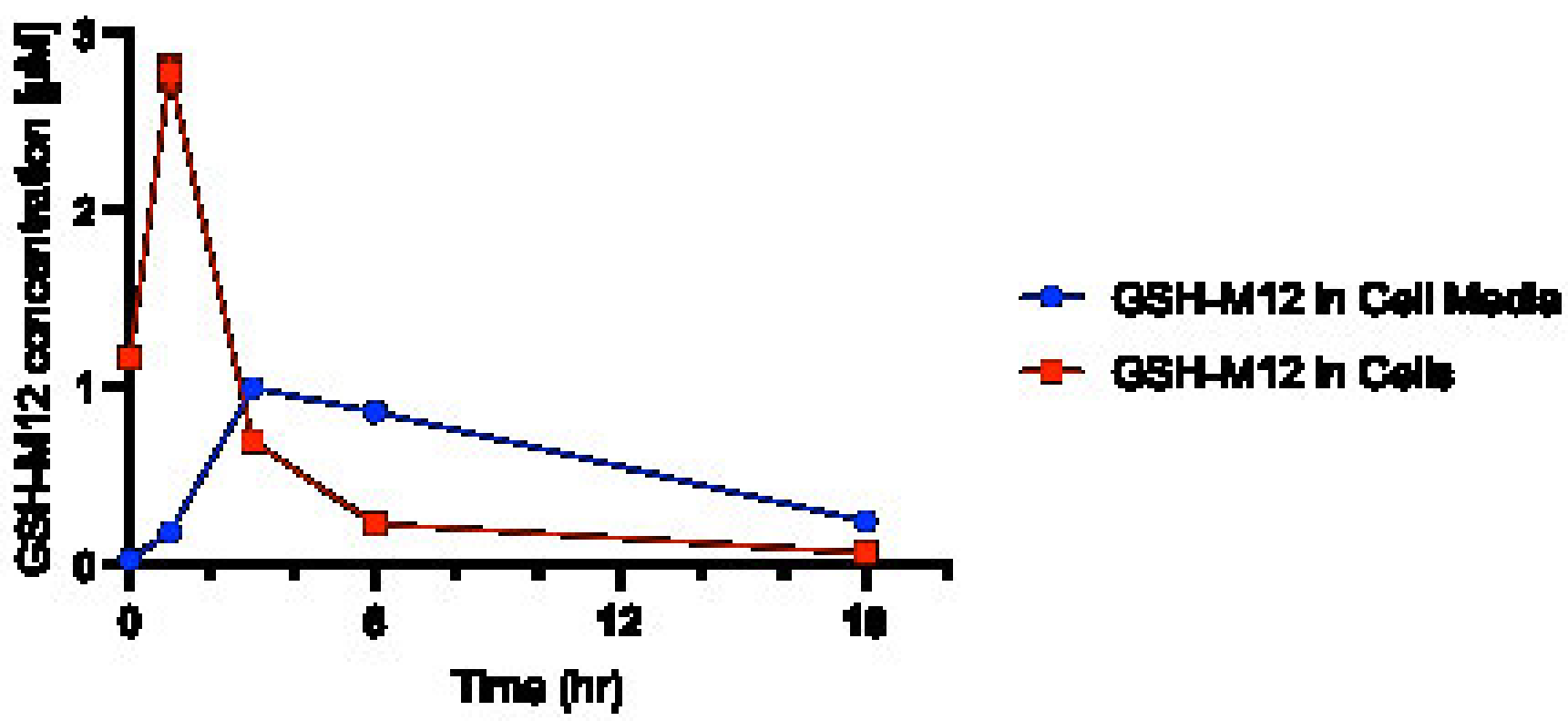

Author Response Figure 10. K562 cells were treated with M12 (10 µM), and GSH-M12 formation was quantified by measuring its concentration in both media and cells.

c. Given that glutathione is typically abundant in cells, could free GSH alone occupy the DCAF11 cavity, thereby necessitating higher M12 concentrations and longer exposure times for effective degradation?

Author Response: We agree with the reviewer and show that GSH alone (or other glutathionylated species) can occupy the binding pocket, thereby competing with GSH-M12 (see response to Question #8; Author Response Fig. 8).

11. It is surprising that four independent groups have published studies employing DCAF11; however, none of these works are cited, nor is the identified DCAF11-binding handle compared with previously reported ones. The authors should include a discussion and contextual comparison with these studies.

Author Response: We thank the reviewer for pointing this out and apologize for the oversight citing these studies and agree that while the other studies report different mechanism, it is important to put our findings in context. The mentioned studies are now referenced in the manuscript (Line 312).

"Several groups have reported DCAF11-recruiting molecules, establishing DCAF11 as an E3 ligase that can be reprogrammed for targeted protein degradation. Electrophilic PROTACs that covalently modify cysteines in DCAF11 have been shown to induce degradation of targets such as FKBP12, androgen receptor, and HDAC, demonstrating that DCAF11 is a ligandable E3 ligase. In addition, several Michael acceptor-containing ligands have been reported to enable DCAF11-mediated targeted degradation. Beyond classical Michael acceptor warheads, nucleophilic SNAr chemistry has been exploited to develop DCAF11-dependent degraders. Two additional DCAF11-mediated degraders lacking electrophilic moieties have also been reported. As these compounds do not contain electrophiles that can be modified by glutathione, they likely operate through mechanisms distinct from glutathionylation-mediated targeted protein degradation."

12. Authors nicely demonstrate dose dependent DDX18 degradation. It would further strengthen the study to include a time-course experiment to better understand the kinetics of DDX18 degradation. Additionally, what is the impact of DDX18 degradation on cellular proliferation?

Author Response: We thank the reviewer for the helpful suggestion. A time-course experiment has now been included in the revised manuscript (**Extended Data Fig. 2d; Author Response Fig. 11a**). DDX18 is a common-essential gene (**Author Response Fig. 11b**). Consistent with the cancer dependency data, M12 treatment induces cell death in K562 cells. The cell-killing effect associated with DDX18 degradation is attenuated upon pre-treatment with MLN4924, which block neddylation-dependent (in this case, presumably DCAF11-mediated) DDX18 degradation (**Author Response Fig. 11c**; **Fig. 2d,e; Extended Data Fig. 2e**).

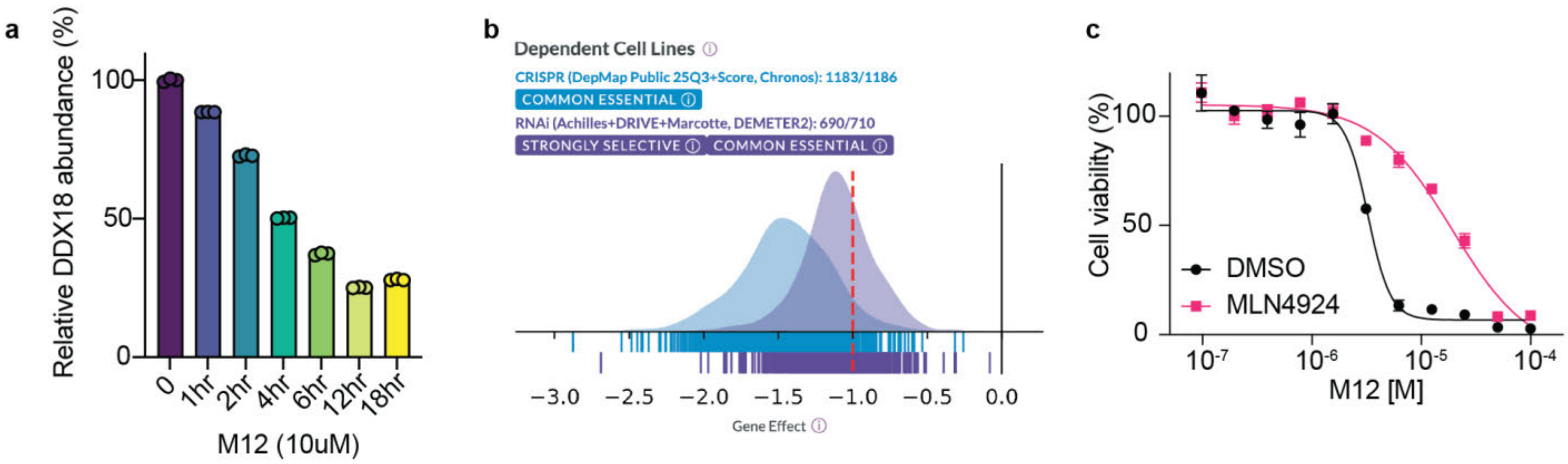

Author Response Figure 11. a. Flow cytometry analysis of DDX18-eGFP reporters in K562$_{cas9}$ cells with M12 in different time points. DDX18 stability was calculated as the eGFP/mCherry fluorescence ratio. **b**. DDX18 cancer dependency from DepMap portal (depmap.org). **c**. Cell viability measured by cell-titer glo assays (72h). Pretreatment of MLN4924 (1h, 500 nM) showed reduction of cytotoxicity by M12, indicating the mechanism of cell death reliant on ubiquitylation. IC_{50} (DMSO): 3.3 μM, IC_{50} (MLN4924): 19.3 μM.

13. Does DDX18 function as a natural substrate of DCAF11? What is the effect of M12 on endogenous substrates of DCAF11, do the authors observe any change in their degradation or affinity in the MS experiments? Since DCAF11 recognizes phosphorylated degrons (e.g., CENP-A, SLBP), can the authors comment on whether M12 requires or bypasses phosphorylation on DDX18?

Author response: We do not rule out the possibility that DDX18 is a natural substrate of DCAF11, however in our *in vitro* analysis, no measurable affinity was detected between DDX18 and DCAF11 (**Author Response Fig. 5**).

To address the reviewer's question regarding potential changes in affinity in DCAF11-IPMS, we re-performed DCAF11-IPMS experiments following M12 treatment (**Extended Data Fig. 1d**; **Author Response Fig. 12**). As shown, no proteins showed significant decrease in abundance upon significant level of DDX18 enrichment. Considering the potential role of DCAF11 in regulating gluthationylated proteins abundance, its activity is likely highly context-dependent, which may explain the lack of significantly downregulated proteins. This observation is consistent with our prior IPMS datasets on CRBN[8], in which proteins involved in protein quality control similarly do not display clear endogenous substrates when IMiD-dependent substrates are enriched.

To address the reviewer's question regarding phosphorylation on DDX18, we assessed potential phosphorylation of DDX18 in complex with GSH-M12 and DCAF11 using both in-solution and in-gel digestion approaches. We found no clear evidence indicating that DDX18 requires or byphasses phosphorylation to engage DCAF11.

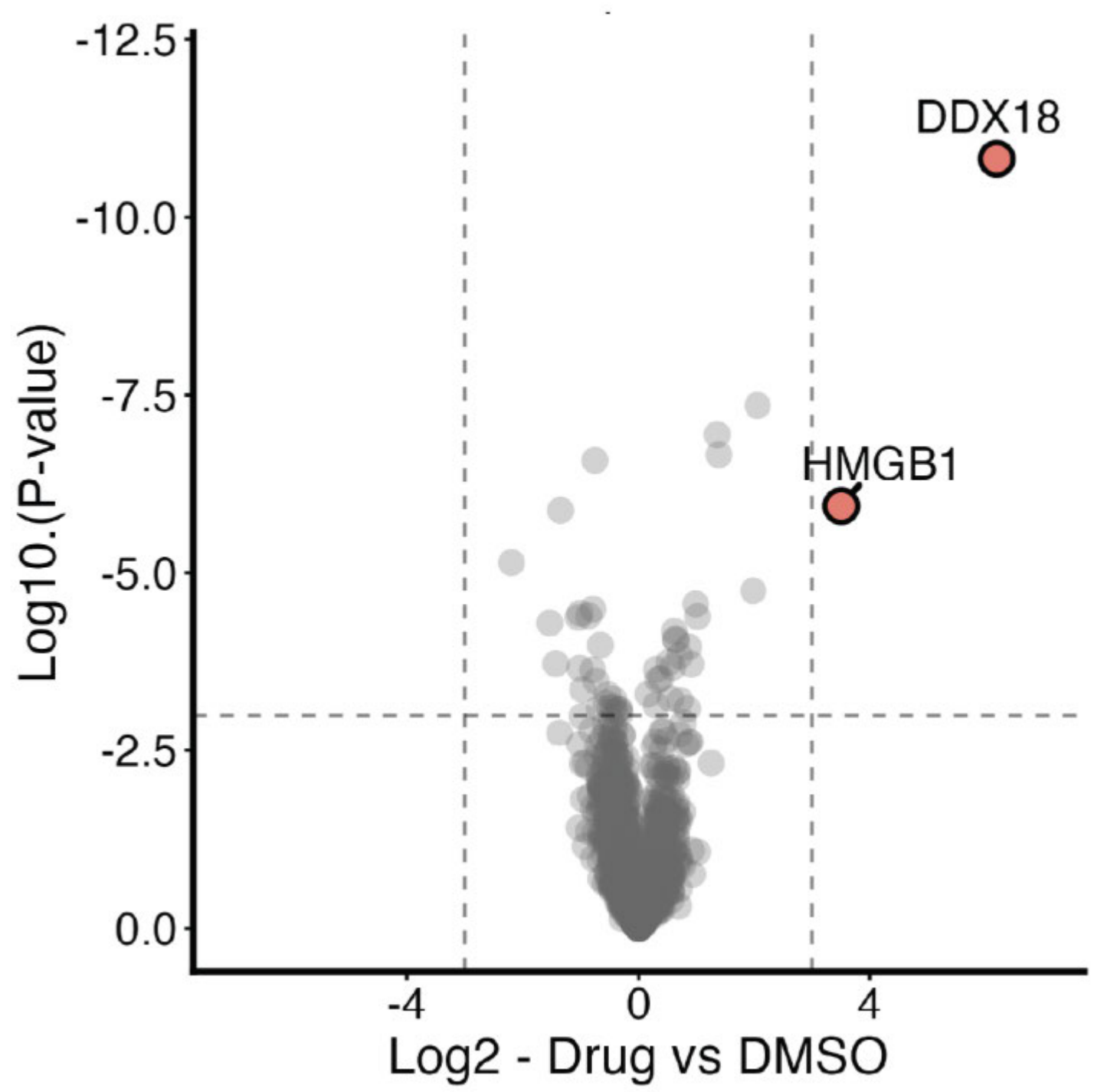

Author Response Figure 12. IP-MS: DCAF11 IP followed by mass spectrometry using Molt4 cell lysates, treated with either DMSO (*n* = 4) or 10 µM M12 (*n* = 4).

14. In Figure 2A, the whole-cell proteomics experiment includes only n = 2 replicates for the M12-treated group versus n = 4 for DMSO. This low replicate number limits statistical confidence. The authors are advised to include at least n = 3 biological replicates per condition.

Author Response: We have previously demonstrated that for profiling of molecular glues schemes like *n* = 2 for treatment and *n* = 4 for DMSO increases throughput without sacrificing data quality[9] and therefore frequently use such an experimental design. But we agree that *n* = 3 is a more widely accepted experimental design and therefore repeated the whole-cell proteomics experiment with *n* = 3 replicates, and have updated the revised manuscript with the new data (**Fig. 2a; Extended Data Fig. 2a; Author Response Fig. 13**), which aligns with the prior data.

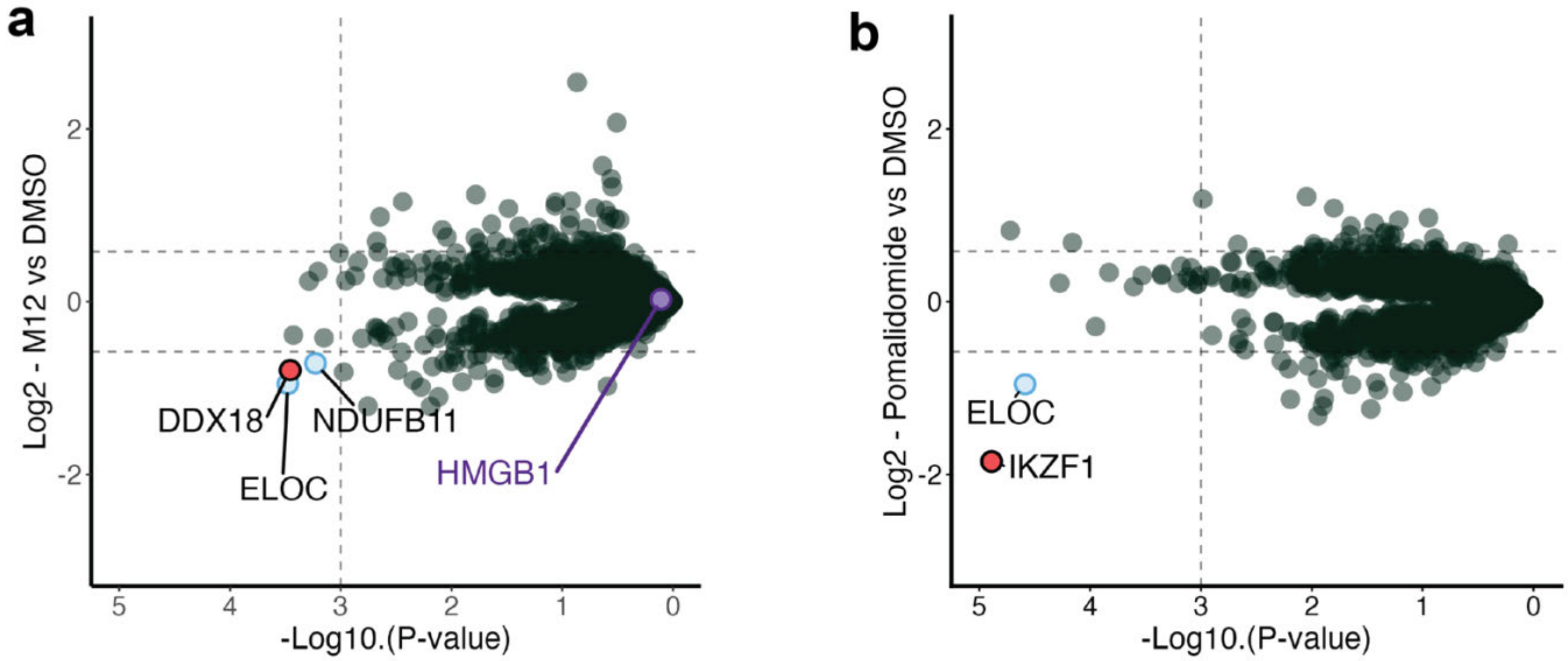

Author Response Figure 13. Whole-proteome quantification of Jurkat cells treated with DMSO, 1 μM pomalidomide, or 20 μM M12 for 6h (*n* = 3). *EloC is a common contaminant in this run, and enriched in both pomalidomide- and M12- treated samples.

15. The corresponding text describing Figures 1G, 1H, and 1I, as well as the relevant citations, appears to be missing from the manuscript. The authors should include these details in the main text.

Author response: We thank the reviewer for pointing out this. We have addressed the relevant citatiation in the revised manuscript (Line 110):

"To identify the small molecule required for DDX18 enrichment by DCAF11, we performed IP experiments with progressively smaller compound pools, revealing a single compound, M12, as the active compound (Fig. 1f-i and Extended Data Fig. 1b,c)."

Referee #2 (Remarks to thAuthor):

In this study, the authors established a new platform for systematic discovery of molecular glues across diverse E3 ligases. By immobilizing multiple E3 ligases on beads and incubating with pooled compounds and cell lysate, this multiplexed immunoprecipitation workflow identifies M12 as a molecular glue that co-opts the E3 ligase DCAF11 to degrade DDX18. Conceivably, this platform could be expanded and applied to other E3 ligases to discover degraders, or other protein-of-interests for the discovery of non-degrader glues.

Another novel finding is that M12 requires the glutathione-S-transferase activity in the cell lysate to become functionalized. Through a series of biochemical dissection, the authors demonstrate that M12 undergoes glutathionylation and the resulting product GSH-M12 fully reconstitutes the glue activity in vitro. This result highlights how drug metabolism can be leveraged for glue discovery.

Finally, structural studies provide mechanistic insights about the interface of the M12-DDX18-DCAF11 complex. Interestingly, GSH-M12 is recognized by a highly conserved pocket on DCAF11, hinting that the physiological function of DCAF11 might be related to GSH regulation. The tight binding of GSH-M12 to DCAF11 also prompts the authors to develop M12-based PROTACs, expanding the current toolbox in targeted protein degradation.

The reviewer finds this study exciting, rigorous, and of immediate interest to a broad audience. It is recommended for publication in Nature, with minor revisions.

Author Response: We thank the reviewer for the positive and encouraging comments of our work. We truly appreciate the time and effort to reviewing our manuscript. We are grateful for the recommendation for publication and we hope we have addressed the remaining questions below.

1. While the authors discussed that "correlating the cytotoxicity of M12 could lead to advances in the development of antitumor agents with improved selectivity", the cytotoxicity of M12 has not been shown or investigated in the study. If there is any cytotoxicity, it would be informative to perform a dose-response curve of M12 and test if DDX18 degradation is responsible for M12 cytotoxicity.

Author Response: We thank the reviewer for the suggestion. M12 treatment induces cell death in K562 cells. The cell-killing effect associated with DDX18 degradation is attenuated upon pre-treatment with MLN4924, which block neddylation-dependent (in this case, presumably DCAF11-mediated) DDX18 degradation (**Author Response Fig. 14**; **Fig. 2d, e; Extended Data Fig. 2e**).

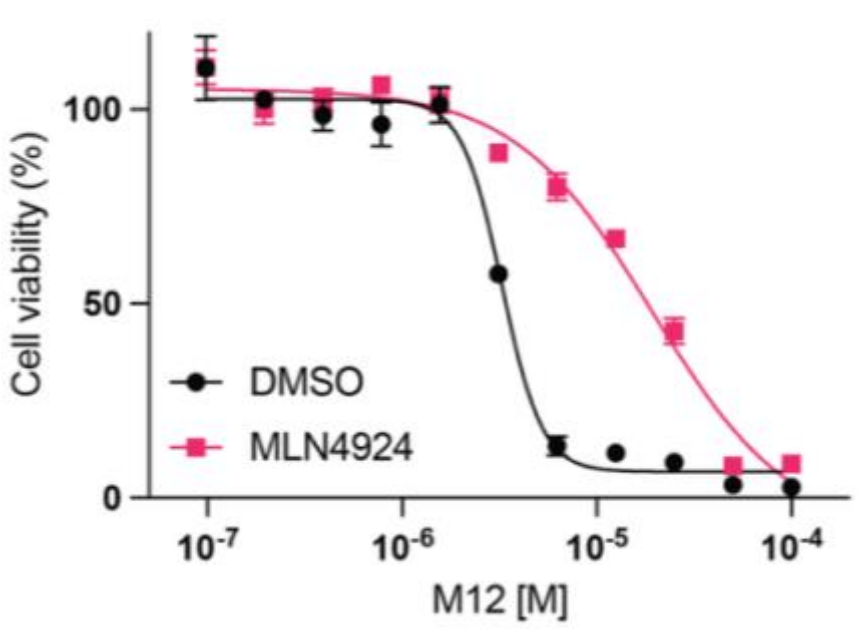

Author Response Figure 14. Cell viability measured by cell-titer glo assays (72h, *n* = 3). Pretreatment of MLN49249 (1h, 500 nM) showed reduction of cytotoxicity by M12, indicating the mechanism of cell death reliant on ubiquitylation. IC_{50} (DMSO): 3.3 μM, IC_{50} (MLN4924): 19.3 μM.

2. In Fig. 4e, the authors mutated residues on DCAF11 extensively to validate the ternary interface. Similar mutation studies should also be done on DDX18. Additionally, DDX18 mutants should be evaluated if they confer resistance to M12 induced degradation, as well as cytotoxicity if there is any.

Author Response: We have generated a number of alanine substitutions in DDX18 on residues that form polar interactions with DCAF11 based on the analysis of our cryo-EM structure. These mutants were evaluated for degradation using our GFP reporter system. All designed mutants showed <50% degradation at M12 concentration below 20 µM, indicating attenuated M12-medated DDX18 degradation (**Author Response Fig. 15a,b**). This data has now been included in the revised manuscript (**Extended Data Fig. 4g**). Due to the essential role of DDX18 in cancer cell viability (**Author Response Fig. 15c**), we were unable to correlate the extent of mutant degradation with M12-induced cytotoxicity with high reliability.

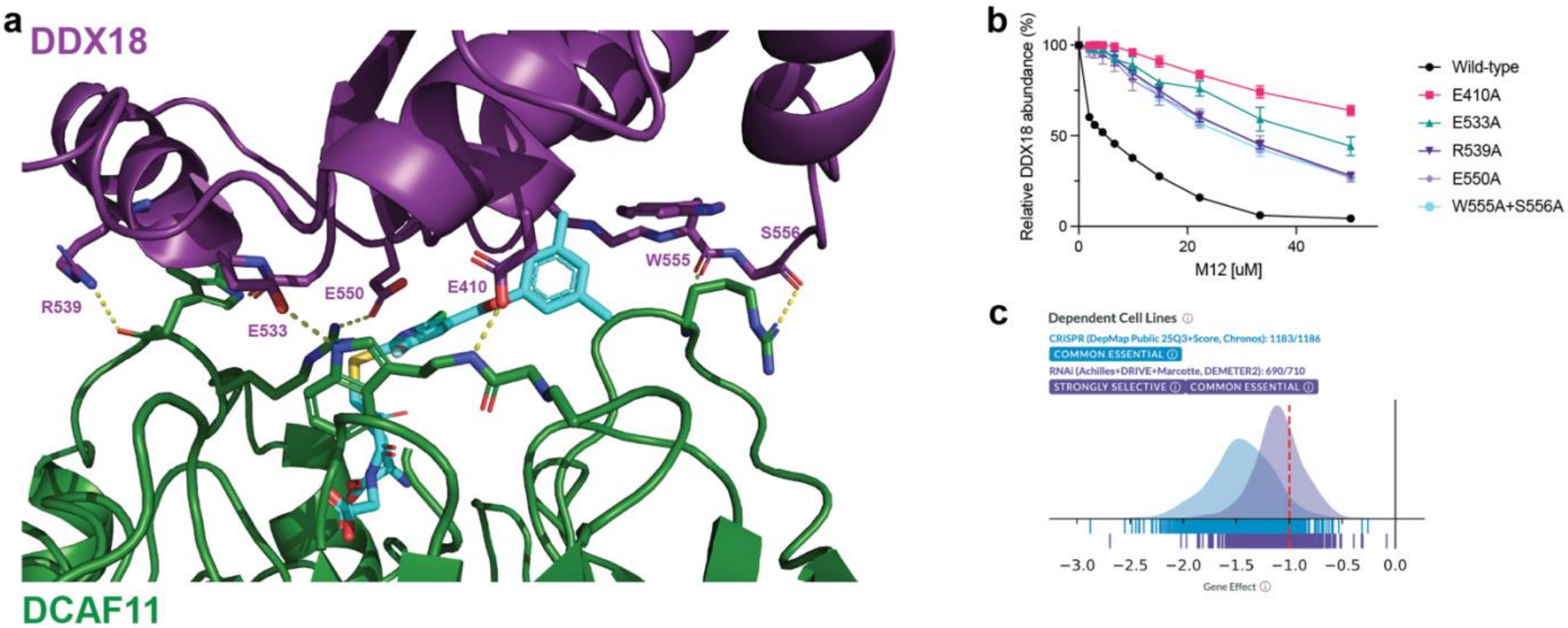

Author Response Figure 15. a. DDX18 forms several polar interactions with DCAF11 residues. **b**. Flow cytometry analysis of DDX18 variants-eGFP reporters in $K562_{cas9}$ cells with M12 for 18 h. DDX18 variants stability was calculated as the eGFP/mCherry fluorescence ratio. **c**. DDX18 cancer dependency from DepMap portal (depmap.org).

3. It will be informative to measure the binding affinity between DDX18 and DCAF11 with or without GSH-M12, which will help substantiate the glue action in promoting ternary complex formation.

Author Response: We thank the reviewer for the suggestion. We did not observe any measurable intrinsic affinity between DDX18 and DCAF11 in contrast to tight binding (K_d = 304 nM) in presence of GSH-M12 (**Author Response Fig. 16**). This data has now been included in the revised manuscript (**Extended Data Fig. 3h**)

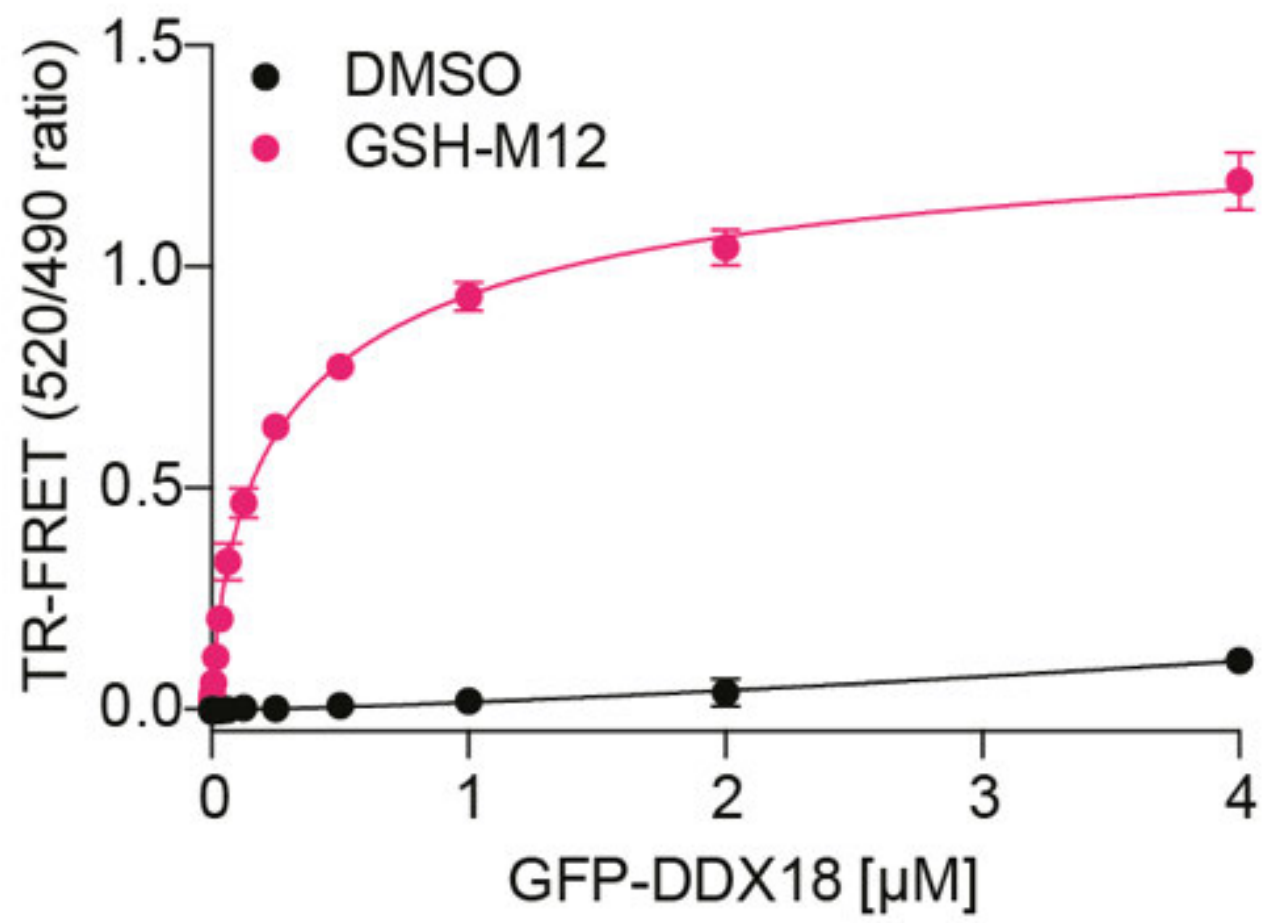

Author Response Figure 17. TR-FRET assay: Titration of DDX18 to teribium-labeled 100nM DCAF11 +/- 200nM GSH M12 (HY-05).

4. Fig 1g-I were not referenced in the main text.

Author response: We thank the reviewer for pointing out this. We have addressed the relevant citatiation in the revised manuscript (Line 110):

"To identify the small molecule required for DDX18 enrichment by DCAF11, we performed IP experiments with progressively smaller compound pools, revealing a single compound, M12, as the active compound (Fig. 1f-i and Extended Data Fig. 1b,c)."

Referee #3 (Remarks to the Author):

I co-reviewed this manuscript with one of the reviewers who provided the listed reports.

Referee #4 (Remarks to the Author):

Yoon et al. describe a new approach for the identification of molecular glue degraders, based on simultaneously multiplexing ligases and compounds in a cellular lysate-based IP-MS screen, aiming to expand the repertoire of E3 ligases that can be employed for the targeted degradation of neo-substrates. The approach is demonstrated using a large compound library screened against a mix of seven E3 ligases, which results in the identification of one compound, M12, that recruits the helicase DDX18 to the E3 ligase DCAF11 for degradation. The authors further show that M12 must undergo glutathionylation by GST in order to act as a molecular glue, and that binding of the glutathionylated M12 to DCAF11 proceeds via a highly conserved binding pocket in DCAF11. These results provide the basis for a novel strategy for targeted protein degradation, using a small molecule prodrug that becomes a molecular glue in vivo by co-opting a specific enzymatic modification, e.g., glutathionylation. The feasibility of this strategy is then demonstrated with the design of other molecular glues, combining glutathionylated M12 with known ligands of a range other proteins of interest, enabling their degradation.

Author Response: We thank the reviewer for the thoughtful evaluation of our manuscript and for the valuable comments and insightful questions, which have improved the work significantly. During the revision we have performed extensive additional experiments to address the reviewers comments as discussed below and in response to other reviewers.

Specific comments:

1. That the multiplexed approach greatly increases the potential to discover modes to recruit additional E3 ligases is not really demonstrated. This study reports one very specific - and certainly very interesting - example, but does not provide enough information to claim that the approach could greatly accelerate discovery of E3 ligase binders. The authors state that the identified molecular glue, M12, would have been unlikely to get identified using other approaches – but this is not due to the multiplexing, but rather the result of using complete cell lysates, containing the enzymes necessary for the glutathione modification.

Author Response: We appreciate the reviewer's positive comments, and thank the reviewer for raising this important point. We agree that the use of cell lysate was critical for the identification of M12 and glutathionylation mechanism as was the use of a diverse chemical library enabled by using compound pools. The value of multiplexing ligases is discussed in more detail in our response to comment #2.

2. The rationale of multiplexing seven ligases in the screen does not seem to be well supported. Multiplexing often comes with a cost, and I wonder how sensitivity was affected by combining seven E3 ligases? What limited the number of ligases? In this context, the use of only two ligases for the proof-of-principle experiment is surprising - if this was meant as a control experiment, why not use seven ligases, including DCAF15 and CRBN? Moreover, it seems unclear in what way the initial combined screen of seven E3 ligases conferred any advantages, as opposed to screening the individual E3 ligases, since following hit detection (e.g., of DDX18) one still has to go back and re-run the assay using the compound pool against each ligase individually. Given that identifying the specific compound responsible for mediating the ligase-DDX18 interaction required another series of IP experiments using progressively smaller compound pools, the ligase multiplexing at the beginning of the pipeline does not seem to provide much of an overall benefit, if any, given that there may be sensitivity-related drawbacks of multiplexing, which remain unexplored.

Author Response: We thank the reviewer for this valuable comment pointing out the inherent trade off between throughput (e.g. through multiplexing) and sensitivity to consider for any larger screening effort. We did not intend to say that multiplexing is the only possible approach, but rather intended to demonstrate the feasibility of a

multiplexed screen by IP-MS, which before conducting this study at least to us was questionable for two main reasons:

1) The loss of sensitivity in multiplexing ligases and hence reducing the number of copies of each bait.
2) The possibility of compounds in a compound pool (320 compounds / well) competing for the same binding site further reducing sensitivity.

Those two concerns are in our view mitigated by the data presented in this manuscript. While arguably signal to noise/sensitivity is reduced in the multiplexed format (**Author Response Fig. 17a**), the identification of true hits is not impaired as the fold-change enrichment is similar to a single ligase/compound validation experiment (**Author Response Fig. 17b**).

We acknowledge that cost considerations were a practical factor in applying a multiplexing strategy, but we continue to believe that multiplexing can offer advantages - without including multiplexing as our screening strategy, we would have needed to perform roughly seven times more quantitative mass spectrometry analysis. We also want to point out that, within the timeframe of us conducting this study, the throughput and cost of MS-based proteomics have evolved substantially due to advances in instrumentation and data analysis workflows. As a result, higher throughput, non-multiplexed approaches are now increasingly accessible and may be a viable alternative in future studies using a similar lysate based approach.

Regarding the deconvolution step: we appreciate the reviewer's valid question and acknowledge that indeed, addressing it would require individual immunoprecipitations. However, in our workflow, this is a straightforward experiment - seven immunoprecipitations followed by western blot, which can be completed within a day.

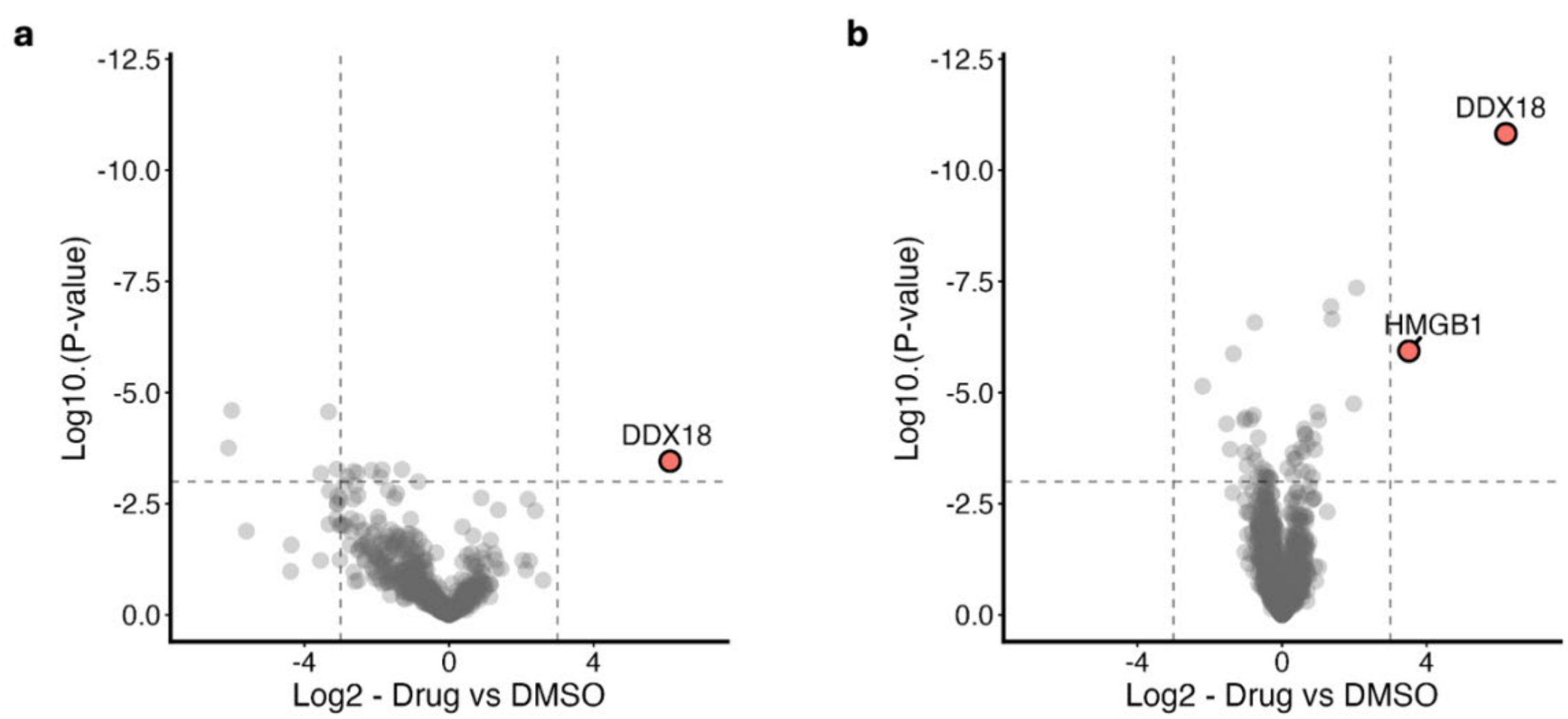

Author Response Figure 17. a. Seven E3 ligases with 320 compounds (1 µM each) (same data as **Fig. 1d**). **b**. A single E3 ligase (DCAF11) with compound M12 (1 µM) (same data as **Extended Data Fig. 1d**). Hits: Fold-change > 3, *p*-value <0.001.

3. Serendipitously, the authors found that DCAF11 may bind specifically to diverse glutathionylated species via a highly conserved GSH binding site. This is a highly significant result that should be highlighted more prominently, also in the abstract. In fact, I think this finding is more significant, in terms of potential impact on drug development and its implications for the biology of E3 ligases, than the rest of the paper, and correspondingly should be explored more thoroughly. For example, does pull-down of DCAF-11 from cell lysate result in enrichment of glutathionylated endogenous metabolites or proteins?

Author Response: We thank the reviewer for the valuable comments and suggestions. We have revised the manuscript and abstract accordingly to highlight the role of glutathionylation in DCAF11.

During the revision period, we have demonstrated in biochemical studies that glutathione itself directly binds to DCAF11 (**Author Response Fig. 18a-e**). [redacted]

[redacted]

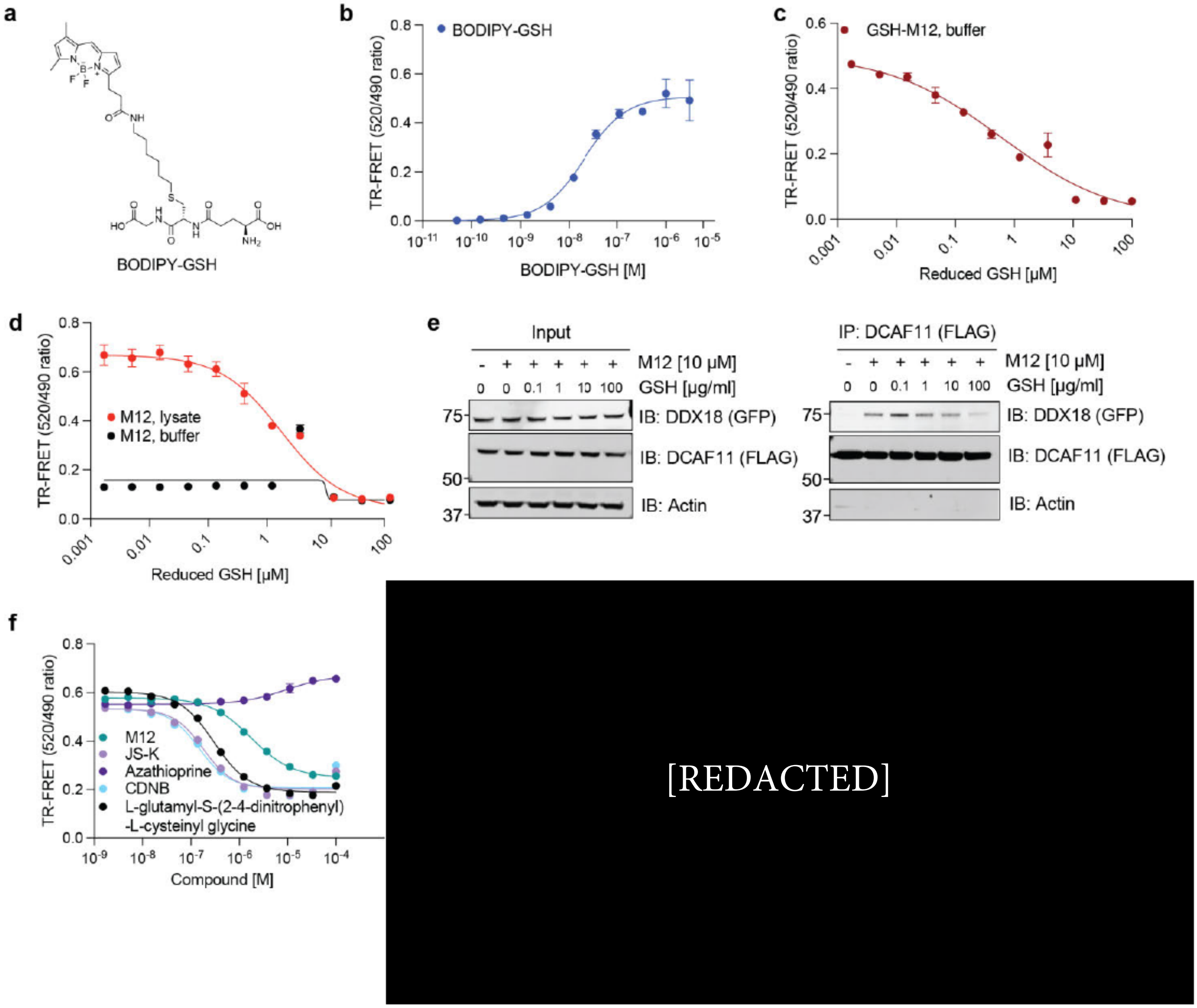

Author Response Figure 18. **a.** Chemical structure of BODIPY-GSH. **b**. TR-FRET: Titration of BODIPY-GSH to 100 nM teribium-labeled DCAF11, demonstrating its direct binding to DCAF11 **c**. TR-FRET: Pre-assembled complex of 300 nM GFP-DDX18 and 100 nM terbium-labeled DCAF11 formed by 50 nM GSH-M12 in regular buffer were treated with an increasing concentration of reduced GSH. **d**. TR-FRET: Pre-assembled complex of 300 nM GFP-DDX18 and 100 nM terbium-labeled DCAF11 by 200nM M12 in lysate or buffer conditions were treated with an increasing concentration of reduced GSH. **e**. Immunoblot of DCAF11 IP +/- M12, with increasing concentration of reduced GSH, using cell lysate expressing DDX18-eGFP. **f-h**. TR-FRET: Pre-assembled 390 nM BODIPY-M12 and 100 nM terbium-labeled DCAF11 in lysate buffer were treated with M12, xenobiotic compounds, [redacted]

4. It remains unclear whether part or the entirety of the structure of M12 itself has any part in mediating the interactions of the various glutathione conjugates used as DCAF11-based PROTACs with DCAF-11. Is a glutathione moiety alone sufficient? This would be of interest also in the context of the very interesting hypothesis that DCAF11 has evolved to recognize glutathionylated substrates. Based on the presented results, it seems likely that the structure of M12, beyond its facility to get glutathionylated, is needed only to confer specificity to

DDX18. The authors should demonstrate whether, as proposed, glutathionylation alone is sufficient to get DCAF11 binding.

Author Response: We appreciate the reviewer's suggestions. We have performed a series of experiements to address these points that have been added to the manuscript. To investigate whether GSH itself directly binds to DCAF11, we performed several experiments, all of which demonstrate that GSH alone binds to DCAF11. This data has been included in the revised manuscript (**Extended Data Fig. 5a-e** and **Author Response Fig. 18a-e**).

Relatedly, the structures of HY-08, 09, and 10 do not appear to contain M12, as suggested in the text, but rather integrate modified versions lacking the fluoride moiety and a methyl group (unless these are errors in the structures shown in the Figures).

Author Response: We thank the reviewer for pointing this out and apologize for the confusion regarding the structure. The HY-08, 09, and 10's structures shown in the figures are correct, and those PROTACS were designed based on our SAR study results, which were not included in the initially submitted manuscript. For DCAF11 binding, replacing *5*-F to hydrogen retains affinity to DCAF11 without recruiting DDX18 (**Author Response Fig. 19a-c**). Based on our structural analysis (**Fig. 4**), the removed methyl group is required for the interaction with DDX18 but is not essential for DCAF11 binding. Considering synthetic accessability and the SAR results, we selected the current scaffold lacking *5*-F in the pyridine and one methyl group in the phenyl ring, as shown in the manuscript.

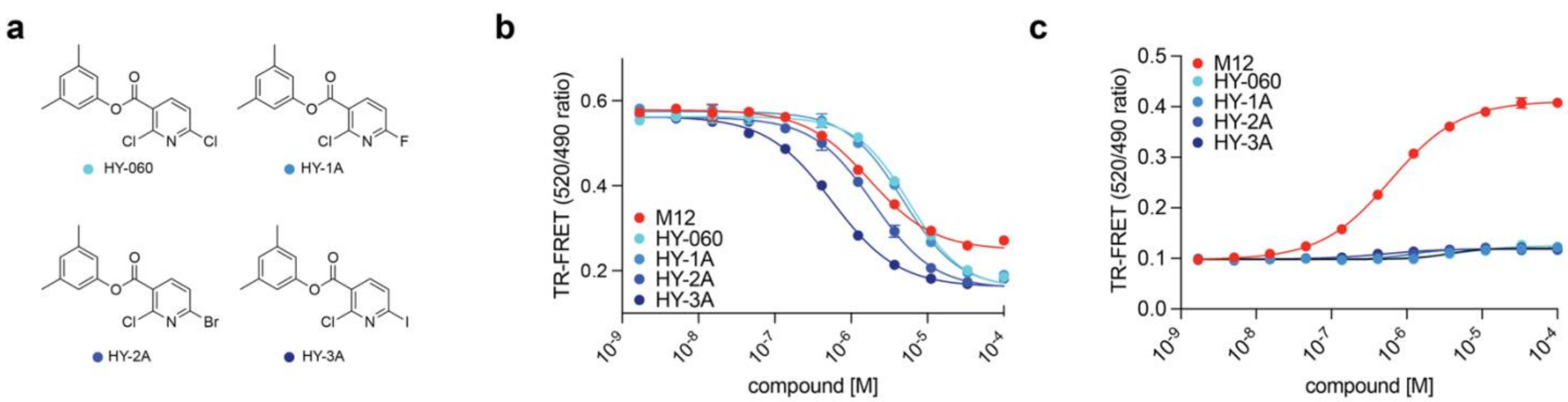

Author Response Fig. 19. a. Chemical structure of M12 analogs containing other halogen atoms. **b**. TR-FRET: Pre-assembled 390 nM BODIPY-M12 (HY-06) and 100 nM terbium-labeled DCAF11 were treated with M12 or its analogs. **c**. TR-FRET: Pre-assembled 300 nM GFP-DDX18 and 100 nM terbium-labeled DCAF11 were treated with M12 or its analogs.

Minor points

1. Methods for the synthesis and spectroscopic characterization for HY-05 and the other M12-derivatives seem to be missing.

Author Response: We thank the reviwer for pointing this out. We have now included all the synthetic procedures and compound charactersization in the supplementary materials.

2. There seems to be no mention of what DDX18 actually is. While not material to the narrative, the authors

should add that this is a DEAD-box RNA helicase that plays a key role in RNA metabolism affecting, ribosome biogenesis, cell cycle progression, and maintenance of stem cell pluripotency.

Author Response: We thank the reviewer for the suggestions. We have now included the description of DDX18 in our updated manuscript (line 105).

"Using IP-MS for each pool, we found that the DEAD-box RNA helicase DDX18, a key regulator of RNA metabolism, ribosome biogenesis, cell cycle progression, R-loop-mediated genome stability, and stem cell pluripotency"

3. Figure 3: The Figure should show the actual "exact mass" of either GSH-M12, 584.11439, or, more appropriately, of the corresponding m/z as detected in ESI+ mode, m/z 585.12167 (and analogously for M12 itself). the authors should also clarify whether these data were acquired in high-resolution/"exact" mass mode, or at low (unit-mass) resolution.

Author Response: We thank the reviewer for pointing this out. We have updated the figure with *m/z* values as suggested. The data in Fig. 3 were acquired at low (unit-mass) resolution, which is now clarified in our method section. Please note that we also performed high-resolution mass in the M12 metabolism studies and confirmed the HRMS value.

Reference

1 Nebert, D. W. & Vasiliou, V. Analysis of the glutathione S-transferase (GST) gene family. *Hum Genomics* **1**, 460-464 (2004). https://doi.org/10.1186/1479-7364-1-6-460

2 Shami, P. J. *et al.* JS-K, a glutathione/glutathione S-transferase-activated nitric oxide donor of the diazeniumdiolate class with potent antineoplastic activity. *Mol Cancer Ther* **2**, 409-417 (2003).

3 Stoelting, M. S. & Tjeerdema, R. S. Glutathione-dependent biotransformation of 1-chloro-2,4-dinitrobenzene in arterial and venous blood of the striped bass (Morone saxitilis). *Aquat Toxicol* **50**, 177-187 (2000). https://doi.org/10.1016/s0166-445x(99)00107-1

4 Eklund, B. I., Moberg, M., Bergquist, J. & Mannervik, B. Divergent activities of human glutathione transferases in the bioactivation of azathioprine. *Mol Pharmacol* **70**, 747-754 (2006). https://doi.org/10.1124/mol.106.025288

5 Dalle-Donne, I., Rossi, R., Colombo, G., Giustarini, D. & Milzani, A. Protein S-glutathionylation: a regulatory device from bacteria to humans. *Trends Biochem Sci* **34**, 85-96 (2009). https://doi.org/10.1016/j.tibs.2008.11.002

6 Ichikawa, S. *et al.* The E3 ligase adapter cereblon targets the C-terminal cyclic imide degron. *Nature* **610**, 775-782 (2022). https://doi.org/10.1038/s41586-022-05333-5

7 Muhar, M. F. *et al.* C-terminal amides mark proteins for degradation via SCF-FBXO31. *Nature* **638**, 519-527 (2025). https://doi.org/10.1038/s41586-024-08475-w

8 Baek, K. *et al.* Unveiling the hidden interactome of CRBN molecular glues. *Nat Commun* **16**, 6831 (2025). https://doi.org/10.1038/s41467-025-62099-w

9 Bushman, J. W. *et al.* Proteomics-Based Identification of DUB Substrates Using Selective Inhibitors. *Cell Chem Biol* **28**, 78-87 e73 (2021). https://doi.org/10.1016/j.chembiol.2020.09.005

Referees' comments:

Referee #1 (Remarks to the Author):

The authors have carefully addressed comments from the first round of review. I would like to acknowledge the considerable effort that has gone into revising the manuscript. The additional experiments, thoughtful rewriting, and improved presentation have strengthened the work and made the main conclusions more compelling. Given that both xenobiotic compounds and endogenous metabolites can engage DCAF11 through glutathionylation, these findings suggest that DCAF11-dependent molecular glue mechanisms may be more widespread than currently recognized. Congratulations to this impressive study and the important discoveries reported here.

Author Response: We thank the reviewer for the careful evaluation of our manuscript and for the insightful comments and constructive feedback provided throughout the revision process.

I have only a few minor points to consider.

1. The authors demonstrate that DCAF11 binds GSH. It would be interesting to know whether the GSH-binding pocket is playing a regulatory role in DCAF11 ubiquitination activity or contributing primarily to substrate recognition, or both. A brief discussion of this point would be helpful.

Author Response: This is a great question for further investigation. At present, our biochemical and structural data support a role for the GSH-binding pocket in substrate recognition, as GSH moiety serves as the key molecular feature mediating DCAF11 engagement with glutathionylated species. Whether this binding pocket also regulates the ubiquitination activity of DCAF11 is an intriguing possibility that will require further investigation.

2. Considering the high levels of GSH in tumor cells and its ability to bind DCAF11, how feasible would it be to employ DCAF11 as an E3 ligase for oncology therapeutics?

Author Response: Our data suggest that cellular GSH does not preclude therapeutic use of DCAF11, though we agree that a therapeutic agent may compete for binding with GSH since our data show that excess free GSH can compete with binding of the M12-GSH conjugate to DCAF11.

In contrast, endogenous GSH levels may be leveraged as part of the activation mechanism for a novel therapeutic. In the case of M12, GSH conjugation is required to generate the active DCAF11-binding species, and the resulting GSH–compound adduct engages a defined pocket in DCAF11 to promote neosubstrate recruitment. Thus, abundant intracellular GSH could support formation of the active glue species rather than simply competing with a therapeutic agent for DCAF11 binding.

Therefore, the efficacy of this mechanism is likely determined not only by GSH abundance itself, but also by cellular redox homeostasis, including the balance between reduced and oxidized GSH (GSH/GSSG), glutathione turnover, and related redox metabolic states. Tumor cells often maintain altered redox state and distinct glutathione dynamics compared with normal cells, which may create an opportunity for preferential activation in certain cancer contexts. We speculate that

specific tumor types or cellular contexts with favorable GSH redox states could be leveraged for this mechanism while maintaining a useful therapeutic window. Further studies of the interplay between GSH metabolism, redox regulation, and DCAF11-mediated neosubstrate recognition will be important for future drug development.

Referee #2 (Remarks to the Author):

The authors have adequately addressed the concerns from this reviewer. Congratulations to the authors again for the exciting story.

Author Response: We thank the reviewer for the positive evaluation of our manuscript and for the valuable feedback provided during the review process.

Referee #3 (Remarks to the Author):

I co-reviewed this manuscript with one of the reviewers who provided the listed reports.

Referee #4 (Remarks to the Author):

The authors have comprehensively addressed most of my concerns. The added data significantly strengthen the narrative, and the changes to the abstract as well as the added paragraph in the Discussion highlighting the implication of the finding that DCAF11 may bind specifically to diverse glutathionylated species via a highly conserved GSH binding site better highlight broader implications.

Author Response: We thank the reviewer for the positive assessment of our revised manuscript and for the constructive feedback provided throughout the review process. The reviewer's insightful comments helped us improve the manuscript, and we are pleased that the additional experiments and textual revisions have addressed the reviewer's concerns and strengthened the study.

Minor remaining concerns:

- I still find the focus on multiplexing in the initial part of the Results distracting. The authors seem to agree that any advantages of multiplexing may be minor and diminishing as MS costs come down, so there's really no reason to elaborate much on this. Further, the critical use of cell lysate in the assay is not highlighted, which, while not unique, distinguishes the screen in this paper from others.

Author Response: We agree that the cost advantage of multiplexing may diminish as mass spectrometry technologies continue to advance and become more accessible. However, our primary intention was not to emphasize cost reduction, but rather to demonstrate the feasibility and utility of a multiplexed screening platform for molecular glue discovery. Because multiplexing approaches are often perceived as challenging to implement in this context, we aimed to

demonstrate that such a platform can be successfully established and applied to molecular glue discovery. We have minimized the emphasis on multiplexing.

We agree that the importance of using cellular lysates was not sufficiently emphasized in the original manuscript. To address this point, we have revised the text to explicitly highlight the role of cell lysates in the assay.

Line 67-75: *"In this study, we developed a high-throughput workflow for the unbiased discovery of molecular glues by mass spectrometry. Multiplexing of ligases in combination with pooled compounds and cell lysates enables unbiased screening across diverse chemical libraries, facilitating the identification of new E3 ligases exploitable for targeted protein degradation. Importantly, the use of cell lysates allows the screen to retain endogenous enzymatic and metabolic activities that may be required for compound activation or target engagement. This approach led to the identification of a novel molecular glue degrader that reprograms the CUL4-RBX1-DDB1-DCAF11 (CRL4^{DCAF11}) ubiquitin ligase. Notably, the identified molecular glue requires an enzyme-mediated gain of function activity, which is unlikely to be identified by other approaches."*

- Figure 3f does not show the synthesis of GSH-M12, only its structure.

Author Response: We thank the reviewer for pointing this out. To clarify this, we have revised the figure legend to explicitly indicate that HY-05 corresponds to synthetic GSH-M12 and to direct readers to the synthetic route provided in the Supplementary Information.

:Extended Data Fig.3f legend: Chemical structure of HY-05 (Synthetic GSH-M12, see Supplementary Information for the synthetic route)

- Assay concentrations of M12 and GSH-M12 are missing in many Figures, e.g., AR Figure 4.

Author Response: We thank the reviewer for this comment. We have ensured that the assay concentrations of M12 and GSH-M12 are specified in all relevant figure legends (e.g. Extended Data Fig. 3i).
